# Intranasal GSK2245035, a Toll-like receptor 7 agonist, does not attenuate the allergen-induced asthmatic response in a randomized, double-blind, placebo-controlled experimental medicine study

**DOI:** 10.1371/journal.pone.0240964

**Published:** 2020-11-09

**Authors:** Hilary Siddall, Diana Quint, Hitesh Pandya, Will Powley, Shaila Shabbir, Jens M. Hohlfeld, Dave Singh, Laurie Lee

**Affiliations:** 1 Research and development, GSK, Stevenage, Hertfordshire, United Kingdom; 2 Respiratory Therapeutic Area, GSK, Stevenage, Hertfordshire, United Kingdom; 3 Biostatistics, GSK, Stevenage, Hertfordshire, United Kingdom; 4 Immuno-Inflammation Global Clinical Sciences & Delivery, GSK, Stevenage, Hertfordshire, United Kingdom; 5 Airway Research, Fraunhofer Institute for Toxicology and Experimental Medicine (ITEM), Hannover, Germany; 6 Member of the German Center for Lung Research, Hannover, Germany; 7 Department of Respiratory Medicine, Hannover Medical School, Hannover, Germany; 8 Respiratory, Medicines Evaluation Unit, Manchester, United Kingdom; 9 Clinical development, GSK, Philadelphia, Pennsylvania, United States of America; Tongji Hospital of Tongji Medical College of Huazhong University of Science and Technology, CHINA

## Abstract

**Background:**

Allergic asthma is a heterogenous disorder predominantly driven by a type 2 inflammatory response to aeroallergens. Therapeutic modulation to rebalance these type 2 responses may offer clinical benefit for allergic respiratory inflammatory diseases, with the potential for disease modification. GSK2245035, a selective toll-like receptor-7 agonist, preferentially stimulates the induction of type 1 interferon alpha, reducing type 2 responses.

**Objective:**

This study investigated whether intranasal GSK2245035 reduced allergen-induced bronchial reactivity in mild allergic asthma.

**Methods:**

This double-blind, placebo-controlled, parallel-group Phase IIa trial randomized (1:1) participants with mild allergic asthma to intranasal GSK2245035 20 ng or placebo once weekly for 8 weeks; follow-up was conducted 1, 4, and 12 weeks after treatment. Allergen-induced late asthmatic response 1 week after treatment was measured as minimum and weighted mean forced expiratory volume in 1 second (FEV_1_) 4–10 hours following bronchial allergen challenge (primary endpoint). Pharmacodynamic and allergic biomarkers, and adverse events, were assessed. A Bayesian analysis framework was used; a posterior probability >0.7 denoted primary endpoint success.

**Results:**

Thirty-six participants were randomized (GSK2245035, n = 22; placebo, n = 14). The percentage attenuation in late asthmatic response was –4.6% (posterior probability: 0.385) and –10.5% (posterior probability: 0.303) for minimum and weighted mean FEV_1_, respectively. Type 2 responses were confirmed by changes in lung function, eosinophils (blood and sputum), interleukin-5 (sputum) and fractional exhaled nitric oxide biomarkers pre- and post-bronchial allergen challenge. However, no treatment effect was observed. Adverse events were reported by 10/14 (71%) and 21/22 (95%) participants in the placebo and GSK2245035 groups, respectively; headache was the most common.

**Conclusions and clinical relevance:**

Although target engagement was observed, weekly intranasal GSK2245035 20 ng for 8 weeks did not substantially attenuate the late asthmatic response in participants with mild allergic asthma. Overall, treatment was well tolerated.

## Introduction

Allergic asthma is a heterogenous disorder characterized by type 2 (T2) predominant airway inflammation [1–4]. Inhaled corticosteroids (which are considered controller therapies) and injectable biologics (such as monoclonal antibodies) target components of T2 inflammation, benefitting individuals with persistent disease by alleviating symptoms and reducing exacerbations; however, evidence of disease modification with inhaled corticosteroids is lacking, and injectable biologics are generally restricted to individuals with more severe asthma [4–6]. Current treatments causing disease remission are lacking; consequently, there is a need for therapies that reduce allergic inflammation across the spectrum of asthma severity, particularly those able to modify airway inflammation over the long term without the need for daily medication.

Toll-like receptors (TLRs) are a family of transmembrane pattern recognition receptors that play a key role in mucosal innate immunity and respiratory allergies [7,8]. TLR7 is activated by single-stranded (ss) RNA [7]. In humans, TLR7 is primarily found in the endosomal compartment of plasmacytoid dendritic cells (pDC) and B cells [7,9], and upon activation by ssRNA, induce the type 1 IFN pathway [8,10]. In animal models of asthma, TLR7 agonism downregulates T2 airway inflammation [11,12].

GSK2245035 is a highly selective TLR7 agonist that preferentially stimulates the induction of interferon-alpha (IFNα) [8]. In an experimental study involving participants with allergic rhinitis, Ellis et al. [13] found that, when compared with placebo therapy, intranasal treatment with GSK2245035 (20 ng or 80 ng) once weekly for 8 weeks reduced nasal symptom scores and allergic biomarkers following nasal allergen challenge. These effects were sustained for up to 3 weeks post treatment [13]. Participants who received GSK2245035 20 ng were eligible to participate in a 1-year follow-up study; trends for reductions in allergic biomarkers in response to nasal challenge were also observed 1 year post treatment [13]. These findings suggested that GSK2245035 has disease-modifying activity in allergic rhinitis.

The present study investigated whether intranasal administration of GSK2245035 20 ng once weekly for 8 weeks reduced allergen-induced bronchial reactivity by rebalancing T2 inflammation in mild allergic asthma. We hypothesized that administering GSK2245035 via the intranasal route would expose local TLR7-expressing dendritic cells to drug and allergens concomitantly and maximally, thereby eliciting a long-lasting modification of the adaptive immune response to allergen in both upper and lower airways. This proof-of-concept experimental medicine study investigated this hypothesis, aiming to provide insight into the efficacy and tolerability of intranasally administered GSK2245035.

## Materials and methods

### Study design

**Fig 1:**
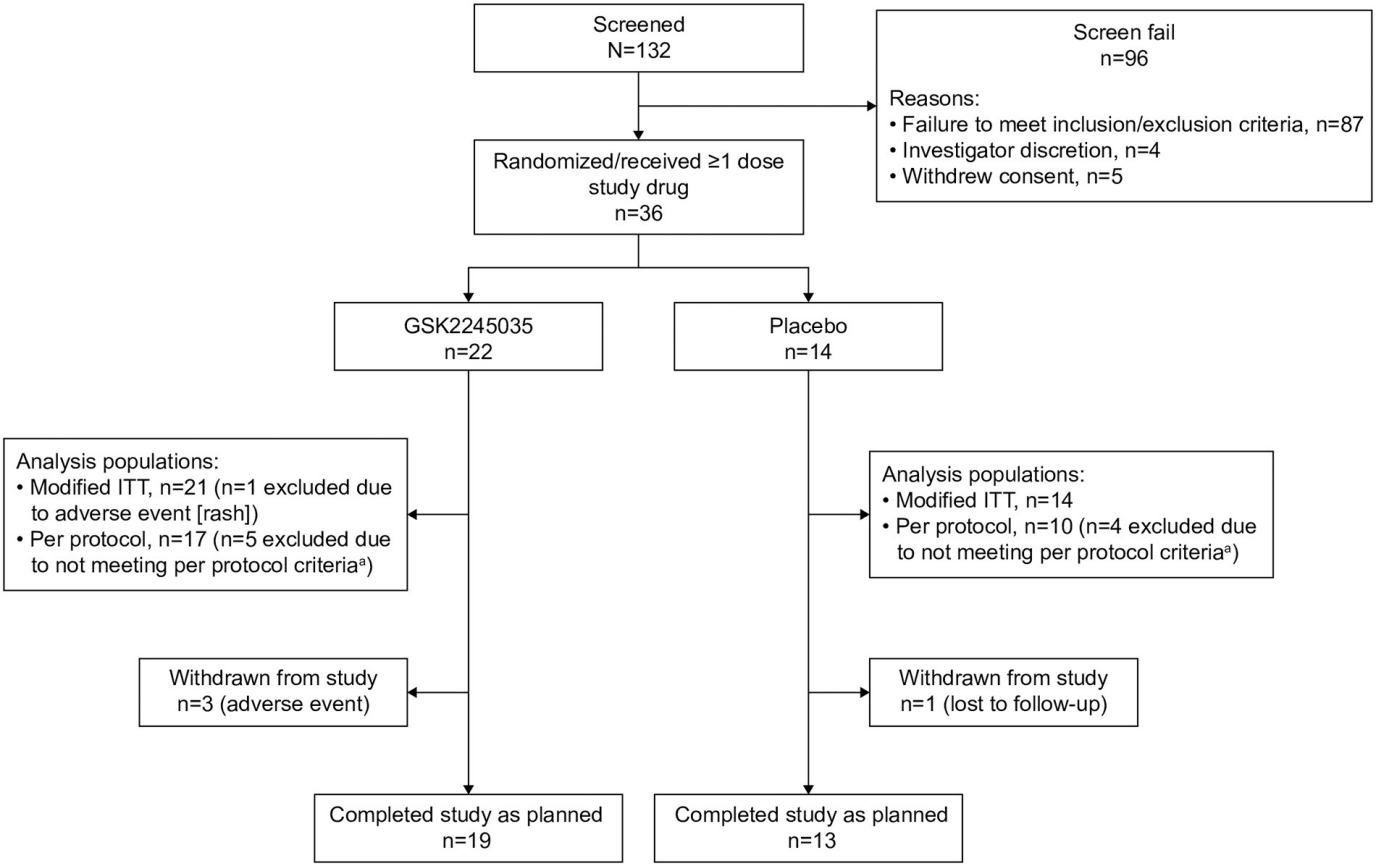
Summary of participant disposition (all participants population). ^a^Reasons for exclusion in placebo arm: incorrect dose of allergen administered at BAC (n = 2; minor difference of 5 SBU/mL), missed one of the planned doses (n = 1), missed first follow-up visit for personal reasons (n = 1). Reasons for exclusion in GSK2245035 arm: missed one of the planned doses (n = 2), challenge not performed for safety (n = 2; FEV_1_ value was too low to permit the challenge to proceed), exceeded average short-acting ß_2_-agonist usage (>2 days per week) during on-treatment period to follow-up visit 3 (n = 1) ITT, intent-to-treat.

**Fig 2:**
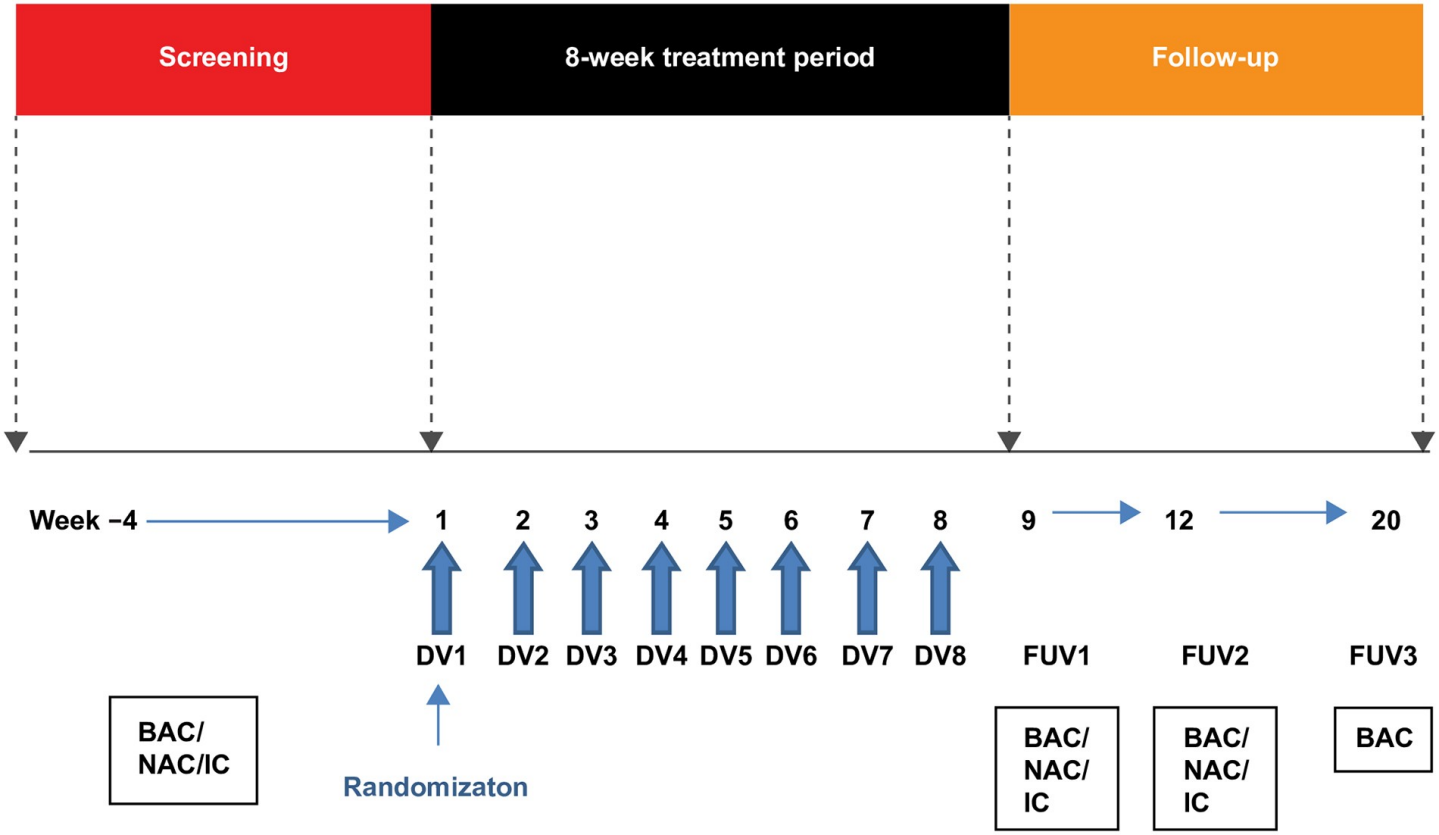
Study design. BAC, bronchial allergen challenge; IC, intradermal challenge; NAC, nasal allergen challenge; DV, dosing visit; FUV, follow-up visit.

This randomized, double-blind (sponsor unblinded), placebo-controlled, parallel-group Phase IIa experimental medicine study enrolled 36 participants with mild allergic asthma from six study sites, all respiratory care and research facilities in the United Kingdom and Germany (GSK study number: 205540; ClinicalTrials.gov: NCT02833974) (Fig 1). The study consisted of a screening period, an 8-week treatment period, and a 3-month follow-up period (Fig 2).

Steroid-naive participants with mild asthma who demonstrated an early and late asthmatic response when challenged with one of four common perennial or seasonal inhaled aeroallergens (cat dander, birch pollen, house dust mite, or grass-mix) were administered intranasal GSK2245035 20 ng or placebo once weekly for 8 weeks. Planned enrolment was approximately 48 participants (24 per treatment arm to obtain 20 evaluable per arm at follow-up visit 1). Participants were challenged for bronchial reactivity with a single allergen based on skin prick test responses. Randomization was stratified by center, and also according to ‘presumed’ or ‘unknown’ allergen exposure during the treatment period. For example, a participant who was challenged with grass pollen and whose treatment was expected within the grass pollen season, or a participant who was sensitized to animal dander who had ongoing household or occupational exposure may be ‘presumed’ to have exposure. Exposure to certain perennial allergens, such as dust mites, could not be presumed and was considered ‘unknown’. If participants were polysensitized, an allergen to which they had presumed exposure was selected in order to maximize concomitant allergen exposure. The same allergen was used for both bronchial allergen challenge and nasal allergen challenges. Allergens were provided by Allegropharma [14] and ALK Abelló, Denmark [15].

The randomization schedule was generated prior to study commencement by the GSK Clinical Statistics Department (Stevenage, Herts, UK) using validated internal software, RandAll NG. Interactive Response Technology was used to assign randomization and drug containers. After treatment was assigned to a participant, the randomization number could not be reused. The study was reviewed and approved by an institutional review board and local research ethics committees prior to commencement and conducted in accordance with the International Council for Harmonisation of Technical Requirements for Registration of Pharmaceuticals for Human Use Good Clinical Practice ethical principles, and as outlined in the Declaration of Helsinki [16]. Written informed consent was provided by each participant prior to study commencement. The study has been reported according to the Consolidated Standards of Reporting Trials (CONSORT) guidelines (S1 Checklist) [17]. The approved study protocol (S1 File) and details of the research ethics committees (S2 File) can be found in Supporting information.

### Participant population

Participants with mild allergic asthma treated with short-acting ß_2_-agonist (SABA) alone ≤2 days per week (ie, did not require treatment with inhaled corticosteroid) were enrolled in the study. Males or females (of non-childbearing potential) aged 18–65 years who met the following key inclusion criteria were eligible: diagnosis of asthma; positive skin prick test (wheal ≥3 mm over negative control) to perennial (cat or house dust mite) or seasonal (grass or birch) aeroallergen(s) at screening; pre-bronchodilator forced expiratory volume in second (FEV_1_) >70% predicted normal at screening. Additionally, to be eligible for study participation the following had to be obtained during bronchial allergen challenge at screening: early asthmatic response with ≥20% FEV_1_ decrease 5–30 minutes following the final allergen concentration inducing the early asthmatic response and late asthmatic response with three FEV_1_ decreases of ≥15% 4–10 hours after the final allergen concentration (with two FEV_1_ decreases at consecutive time points). Full study inclusion and exclusion criteria are detailed in S3 File.

### Treatments

Participants received either GSK2245035 20 ng formulated as a solution (10 ng/actuation) or visually matched placebo, administered intranasally (one spray per nostril) once weekly. Investigators and participants were blinded to treatment allocation and the study sponsor was unblinded. The study investigator could unblind treatment allocation in case of an emergency or where knowledge of the study treatment was essential for the welfare of the participant.

### Endpoints

#### Primary endpoint

The primary endpoint was allergen-induced late asthmatic response one week after treatment (at follow-up visit [FUV] 1). Late asthmatic response was measured as minimum FEV_1_ and weighted mean (WM) FEV_1_ 4–10 hours following allergen challenge.

#### Secondary endpoints

Secondary endpoints were allergen-induced early asthmatic response (minimum and WM FEV_1_ 0–2 hours following allergen challenge) 1 week after treatment (at FUV1), and safety. Safety endpoints included incidence of adverse events, serious adverse events, and adverse events of special interest.

#### Exploratory endpoints

Exploratory endpoints were late asthmatic response and early asthmatic response (minimum and WM FEV_1_) 4 and 12 weeks after treatment (FUV2 and FUV3, respectively), pharmacodynamic (PD) and allergic biomarkers, change in fractional exhaled nitric oxide (FeNO), total nasal symptom score (TNSS), and pharmacokinetics following bronchial allergen challenge and nasal allergen challenge.

### Assessments

#### Efficacy

At screening, participants were administered nebulized allergen solutions in increasing concentrations until an early asthmatic response was elicited, and bronchial allergen challenge was performed using the incremental dose method by dosimeter [18]. Incremental doses of allergen were administered based on the post-allergen FEV_1_ value achieved. The total dose of allergen that induced a reduction in early and late asthmatic response was used to calculate the bolus dose for use at subsequent bronchial allergen challenges (S4 File). The same allergen was used for each participant throughout the duration of the study.

Nasal allergen challenge was performed approximately 24 hours following bronchial allergen challenge at screening, and at FUV1 and FUV2 (Fig 2) using the same allergen as for bronchial allergen challenge (dose consistent throughout). The Aptar Pharma Bidose nasal delivery device was used to deliver 100 μL of a fixed allergen dose to each nostril. Nasal lavage and nasal filter eluate samples were collected before and after nasal allergen challenge at screening, FUV1, and FUV2. Intradermal challenge at screening was done using incremental allergen doses of the applicable allergen as for bronchial allergen challenge. Subsequent intradermal challenges were performed using the dose that induced ≥3 mm wheal compared with negative control. For nasal scrapes and intradermal challenges, there were insufficient samples to allow meaningful data review.

#### Safety

Safety assessments were monitoring of adverse events, clinical laboratory tests, vital signs, electrocardiograms, physical examinations, pregnancy, and peak expiratory flow. Adverse events of special interest were cytokine release syndrome (CRS)-related events, such as headache, fever, chills/rigor, nausea, arthralgia, myalgia, vomiting, diarrhea, and hypotension; severity was graded from 0–4 (0 = none; 1 = mild; 2 = moderate; 3 = severe; 4 = disabling/life-threatening).

#### Biomarkers

PD biomarkers: Interferon inducible protein-10 [IP-10] was measured in serum and nasal lavage samples to confirm target engagement. Additionally, monocyte chemoattractant protein 1 (MCP-1), IFNα, IFNβ, interleukin (IL)-1β, IL-6, and tumor necrosis factor alpha (TNFα) levels were measured in serum. The biomarker sampling schedule is detailed in S5 File and biomarker assays detailed in S6 File.

T2 inflammatory biomarkers: To assess T2 inflammation in the lung, IL-5, IL-13, and eosinophils were measured in induced sputum, and to assess T2 inflammation in blood, eosinophil numbers counted. Nasal T2 inflammation was assessed by measuring the following in nasal lavage: allergen specific immunoglobulin A (sIgA), eosinophil cationic protein (ECP), histamine and mast cell tryptase; the following were measured in nasal filter eluate: IFNγ, IL-5, IL-10, IL-13, IL-16, eotaxin, macrophage derived chemokine (MDC) and thymus and activation regulated chemokine (TARC).

#### Fractional exhaled nitric oxide

FeNO was measured using a handheld electronic device in accordance with American Thoracic Society/European Respiratory Society recommendations [19].

#### Total nasal symptom score

Nasal symptom (nasal congestion, rhinorrhea, nasal itch, and sneezing) scores were assessed daily (morning and evening) by participants throughout the 8-week treatment phase up until FUV2. They were documented in participant diary cards and graded as 0 = none, 1 = mild, 2 = moderate, 3 = severe, with a maximum TNSS score of 12.

#### Pharmacokinetics

Blood samples for pharmacokinetic analysis were collected pre-dose and 20 minutes and 1 hour post dose at dosing visits (DV)1, DV4, and DV8.

### Statistical analyses

The study sample size was determined using a simulation-based approach that enumerated the probability of meeting the study success criteria (posterior probability [PP]) of any percentage attenuation >0.7) for ≥1 of the efficacy endpoints under a variety of assumed treatment effects (percentage attenuations) and number of participants per treatment arm. The sample size calculation assumed that the placebo response and the variability of the endpoints in this study were similar to previous studies conducted by GSK (assumptions consistent with the observed data), that the true treatment effect of GSK2245035 was no worse than that in a study of the TLR7 agonist AZD8848 (based on congress presentation data [20]), and that 20 patients had evaluable data at the first the follow-up assessment. Under those assumptions the probability of achieving the study success criteria was approximately 80%.

A pre-planned interim analysis was conducted to assess the number of participants receiving placebo who lost their late asthmatic response post enrolment, as these participants reduced the chances of establishing the primary objective; it was determined that an increase in sample size was not required. However, the study was subsequently terminated due to operational futility in reaching the original recruitment target; this was largely due to challenges with the availability of allergen and study drug.

Study population, safety and biomarker analyses were based on the all participants population (all participants who received ≥1 dose of the study treatment). Both early and late asthmatic response analyses were based on the per-protocol population (all randomized participants who received ≥1 dose of the study treatment and commenced a bronchial allergen challenge at follow-up, and complied with the protocol). Statistical models were fitted separately to each endpoint. These were analyzed using a Bayesian implementation of analysis of covariance with treatment, baseline and allergen exposure (‘presumed’/‘unknown’) in the model; covariates such as center and allergen exposure by treatment interaction were investigated; however, these were not included in the final models (all model parameters used non-informative priors).

To facilitate clinical interpretation, comparisons of GSK2245035 versus placebo for bronchial allergen challenge endpoints were expressed as a percentage of attenuation and its posterior distribution was used to evaluate the study success criteria. Given the exploratory phase of development, the predefined level of certainty required to declare primary endpoint success was PP>0.7 (a type 1 error rate of 30%). The PP of any percentage attenuation was obtained along with 95% credible intervals (95% CrI). For the purposes of sensitivity analyses, minimum and WM late asthmatic response absolute change from baseline at FUV1 were analyzed using blood eosinophil levels as an additional (continuous) covariate and making predictions for percentage attenuation at two predefined levels (100 and 200 cells/μL).

## Results

### Participant disposition and clinical characteristics

A total of 132 participants were screened for eligibility; 96 participants failed screening and 36 were enrolled and randomized (GSK2245035 20 ng, n = 22; placebo, n = 14) (Fig 1). The most common reasons for screen failure were no or insufficient late asthmatic response at screening. Enrollment took place between December 5, 2016 and November 27, 2017; last participant last visit was May 4, 2018. In the placebo arm, 13/14 (93.0%) participants completed the study and 1/14 (7.0%) was withdrawn as lost to follow-up. In the GSK2245035 arm, 19/22 (86.0%) completed the study and 3/22 (14.0%) were withdrawn due to adverse events. Nine participants were excluded due to not meeting per protocol criteria (Fig 1); these participants were included in the all-participants population but excluded from the per protocol population. Baseline demographics are shown in Table 1. Most participants were challenged with ‘unknown’ allergen exposure (placebo, 12/14 [86%]; GSK2245035, 19/22 [86%]).

**Table 1:** Participant demographics (all participants population).

Demographics	Placebo (N = 14)	GSK2245035 20 ng (N = 22)
**Age (years), mean (SD)**	36.6 (12.26)	36.0 (11.65)
**Sex, n (%)**		
Female	2 (14)	1 (5)
Male	12 (86)	21 (95)
**Ethnicity, n (%)**		
Not Hispanic or Latino	14 (100)	22 (100)
**Race, n (%)**		
African American/African Heritage	0	2 (9)
Asian (Central/South Asian) Heritage	0	1 (5)
White	14 (100)	19 (86)
**BMI (kg/m**^**2**^**), mean (SD)**	28.2 (2.85)	26.6 (5.34)
**Height (cm), mean (SD)**	176.0 (10.58)	179.1 (6.14)
**Weight (kg), mean (SD)**	87.1 (9.33)	85.6 (17.61)
**Allergen exposure stratum**		
Presumed allergen	2 (14)	3 (14)
Unknown allergen	12 (86)	19 (86)

BMI, body mass index; SD, standard deviation.

### Late asthmatic response

**Fig 3:**
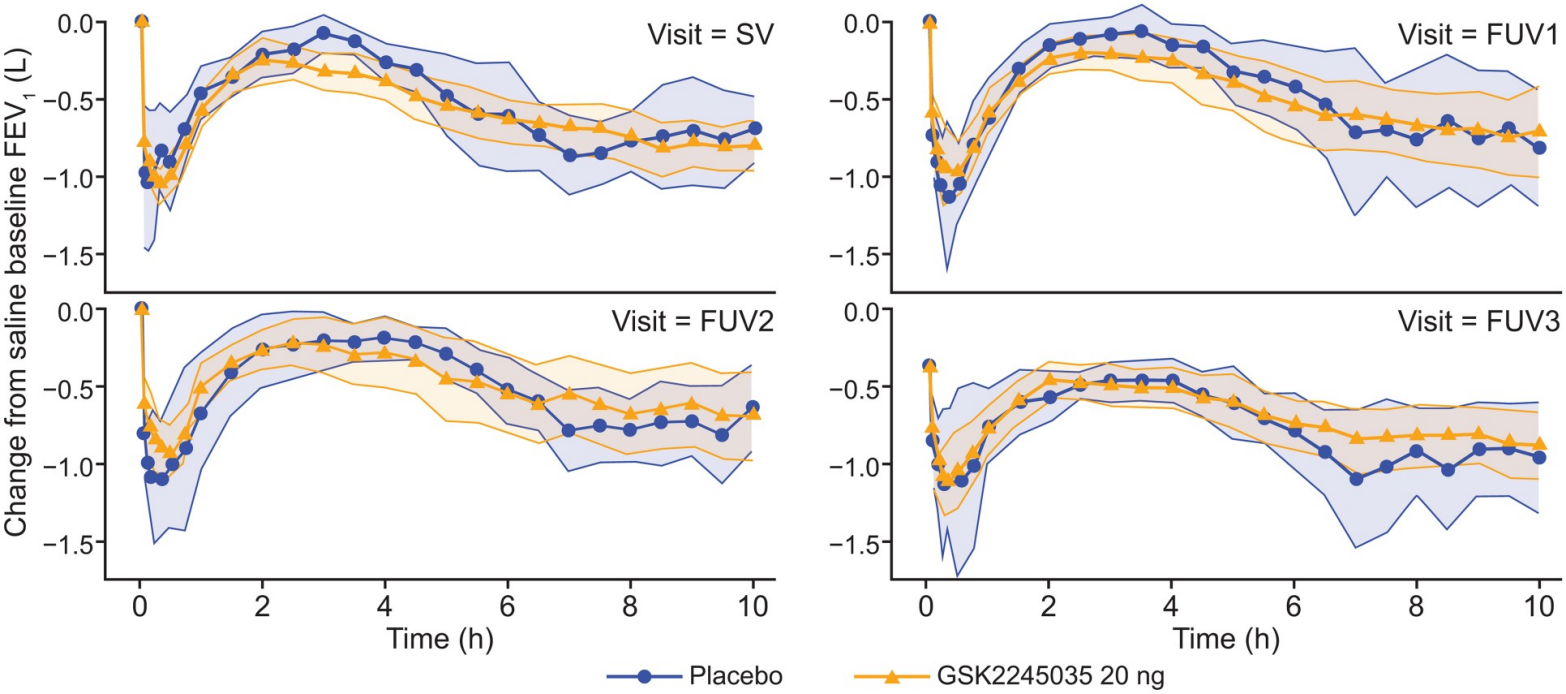
Mean FEV_1_, change from baseline FEV_1_ (following saline inhalation) per time point (per-protocol population). 95% CIs were computed separately for each treatment arm per time point from raw data values. Shaded areas are 95% CIs from each time point merged together FEV_1_, forced expiratory volume in 1 second, FUV, follow-up visit; h, hours; SV, screening visit.

The percentage attenuation (95% CrI; PP) for GSK2245035 versus placebo from baseline to Week 9 (FUV1) in minimum FEV_1_ was –4.6% (–46.50, 23.13; 0.385) and in WM FEV_1_ was –10.5% (–72.62, 24.97; 0.303). Mean FEV_1_ change from baseline over time in both treatment groups is shown in Fig 3. Overall, stratification by eosinophil level in the sensitivity analyses did not have a significant effect, with the exception of the 100 cells/μL strata minimum FEV_1_ (S1 Table). Thus, the predetermined study success criteria were not met for either of the primary endpoints in the per-protocol population. A similar response was observed at FUV2 and FUV3.

### Early asthmatic response

The percentage attenuation (95% CrI; PP) for GSK2245035 versus placebo from baseline to Week 9 (FUV1) was 1.3% (–26.65, 21.63; 0.545) and 4.2% (–37.62, 31.36; 0.603) for minimum FEV_1_ and WM FEV_1_, respectively. Mean FEV_1_ change from baseline over time in both treatment groups is shown in Fig 3. Stratification by eosinophil level in the sensitivity analyses did not have a significant effect, with the exception of the 100 cells/μL strata minimum FEV_1_ (S1 Table). Hence, the early asthmatic response endpoint results were consistent with late asthmatic response endpoints. A similar response was observed at FUV2 and FUV3.

### Safety

Overall, 10/14 (71%) and 21/22 (95%) participants reported an adverse event in the placebo and GSK2245035 groups, respectively; headache was the most common (placebo: 8/14 [57.0%]; GSK2245035: 9/22 [41.0%]), followed by nasopharyngitis (placebo: 3/14 [21.0%]; GSK2245035: 6/22 [27.0%]). Oropharyngeal pain was reported by 7/22 (32%) participants in the GSK2245035 arm and no participants in the placebo arm. Treatment-related adverse events were reported in 4/14 (29%) participants in the placebo group and 14/22 (64%) participants in the GSK2245035 group; headache was the most common (placebo: 2/14 [14%]; GSK2245035, 6/22 [27%]) (Table 2). All treatment-related adverse events were mild or moderate in intensity and the majority were resolved by FUV1. CRS-related events (which were considered adverse events of special interest) were reported by 1/14 (7%) participant in the placebo arm and 5/22 (23%) participants in the GSK2245035 arm; these were self-limiting and the highest severity reported in each treatment arm was grade 2. Three participants withdrew from the study due to adverse events, all in the GSK2245035 arm, due to either “flu-like” symptoms, rash or cough; only rash was considered related to study treatment by the investigator. No deaths or serious adverse events were reported.

**Table 2:** Adverse events (reported by ≥2 participants in any treatment group) and treatment related adverse events (reported by ≥1 participant in any treatment group) (all participants population).

**Adverse event, n (%)**	**Placebo (N = 14)**	**GSK2245035 20 ng (N = 22)**
Any adverse event	10 (71)	21 (95)
Headache	8 (57)	9 (41)
Nasopharyngitis	3 (21)	6 (27)
Upper respiratory tract infection	0	3 (14)
Back pain	0	4 (18)
Oropharyngeal pain	0	7 (32)
Epistaxis	1 (7)	2 (9)
Nasal congestions	0	2 (9)
Nasal dryness	0	2 (9)
Wheezing	0	2 (9)
**Treatment-related adverse events, n (%)**	**Placebo (N = 14)**	**GSK2245035 20 ng (N = 22)**
Any adverse event	4 (29)	14 (64)
Headache	2 (14)	6 (27)
Epistaxis	1 (7)	1 (5)
Nasal dryness	0	2 (9)
Nasal discomfort	0	1 (5)
Nasal inflammation	1 (7)	0
Nasal edema	0	1 (5)
Oropharyngeal pain	0	1 (5)
Rhinorrhea	0	1 (5)
Upper abdominal pain	0	1 (5)
Nausea	0	1 (5)
Odynophagia	0	1 (5)
Nasopharyngitis	0	1 (5)
Rhinitis	0	1 (5)
Upper respiratory tract infection	0	1 (5)
Rash	0	1 (5)
Skin exfoliation	0	1 (5)
Flushing	0	1 (5)
Hypertension	0	1 (5)
Vertigo	1 (7)	0
Malaise	0	1 (5)
Back pain	0	1 (5)
Renal pain	0	1 (5)

### TLR7-induced pharmacodynamic biomarkers

Changes in serum and nasal IP-10 are detailed in Table 3. There was a consistently high PP of increases in mean IP-10, confirming target engagement.

**Table 3:** Summary of serum and nasal lavage interferon inducible protein-10 fold changes (treatment comparison: GSK2245035 20 ng vs placebo) (all participants population).

Sample	Time point	Median fold change	95% CrI	PP^a^
**Serum**	DV1 + 24 h	1.5	1.11, 2.06	0.995
	DV8 + 24 h	2.9	1.67, 5.04	1.000
**Nasal lavage**	DV1 + 24 h	4.8	2.64, 8.85	1.000
	DV4 + 24 h	7.2	2.74, 19.00	1.000
	DV8 + 24 h	14.4	5.92, 35.36	1.000

^a^Any increase in mean response relative to placebo.

CrI, credible interval; DV, dosing visit; h, hours; PP, posterior probability.

### Allergic inflammatory biomarkers

**Fig 4:**
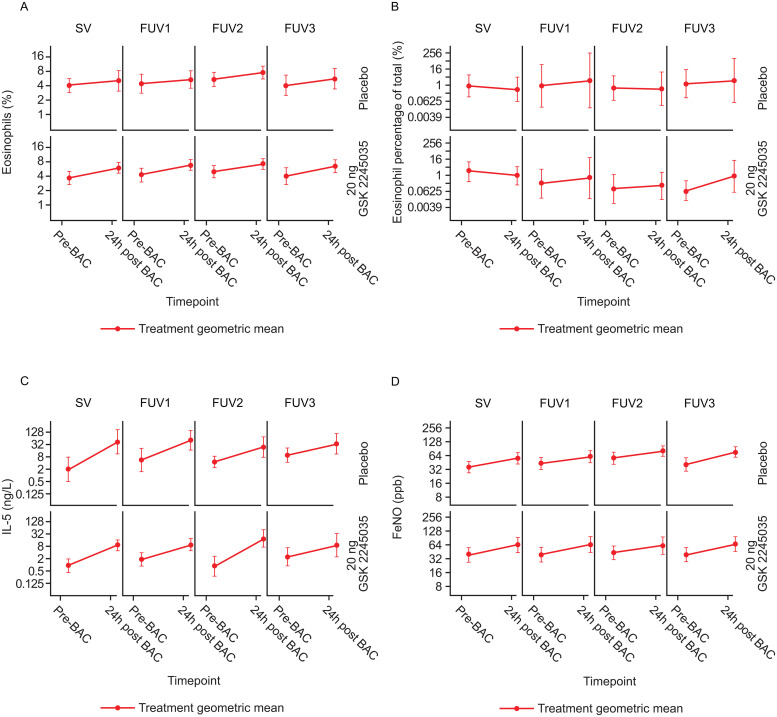
Summary profiles of allergen-induced changes in T2 inflammatory biomarkers pre- and post-bronchial allergen challenge at each time point: A) blood eosinophils; B) sputum eosinophils; C) sputum IL-5^a^; D) FeNO (all participants population; B shows data from sputum-producers only). ^a^The LLQ for IL-5 was 0.19 ng/L. All IL-13 levels were below LLQ (2.22 ng/L), with the exception of one sample. 24h, 24 hours; BAC, bronchial allergen challenge; FeNO, fractional exhaled nitric oxide; IL-5, interleukin-5; FUV, follow-up visit; LLQ, lower limit of quantification; ppb, parts per billion; SV, screening visit.

Blood and sputum eosinophil counts were elevated post-bronchial allergen challenge, consistent with a T2 response, in both groups (Fig 4A and 4B), but no treatment effect was observed. Matched pre- and post-bronchial allergen challenge sputum samples showed an increase in IL-5 levels in both groups (statistical modeling was not attempted due to insufficient data); no treatment effect was observed (Fig 4C).

Nasal allergen challenge-induced change in nasal lavage and nasal filter eluate showed no evidence of a GSK2245035 treatment effect overall on allergic mediators. However, fold changes adjusted for screening were noted in the following mediators: tryptase 0.28 (95% CrI 0.10, 0.78; 0.991 PP decrease), eotaxin 0.54 (95% CrI 0.18, 1.63; 0.867 PP decrease), IFNƴ 2.11 (95% Crl 0.69, 6.47; 0.910 PP increase), sIgA 1.55 (95% Crl 0.65, 3.74; 0.845 PP increase) at FUV1; and sIgA 1.39 (95% Crl 0.65, 2.93; 0.808 PP increase) at FUV2 (S2 Table).

### Fractional exhaled nitric oxide

There were increases in FeNO in both groups at all FUVs, consistent with bronchial allergen challenges (Fig 4D). No treatment effect was observed.

### Total nasal symptom score

Mean weekly WM TNSS was <1 in the placebo group and <1.5 in the GSK2245035 group throughout treatment; the PP of any reduction was <0.9 at all time points. No difference in TNSS was observed between treatment groups (S1 Fig).

### Pharmacokinetics

GSK2245035 was not quantifiable in any plasma sample (lower limit of quantification: 2 pg/mL).

## Discussion

This proof-of-concept study was designed to determine whether treatment with an intranasal TLR7 agonist, GSK2245035, could attenuate the late asthmatic response of the lower airways by reducing T2 inflammation. Target engagement post administration of GSK2245035 was clearly demonstrated by increases in serum and nasal IP-10. Nonetheless, once-weekly intranasal administration of GSK2245035 20 ng over 8 weeks did not sufficiently attenuate the late asthmatic response to meet the predetermined study success criteria. The analyses of early asthmatic response were consistent with this result.

The bronchial allergen challenge model is commonly used to assess novel anti-T2 drugs for the treatment of asthma [18,21–23]. In the present study, clinical and allergen-induced T2 activation responses to bronchial allergen challenge were confirmed by changes in biomarkers in blood (eosinophils) and sputum (eosinophils and IL-5), and by increases in FeNO. However, there was no evidence of a treatment effect of GSK2245035 systemically or in the lung, as demonstrated by the lack of GSK2245035-mediated effects on these biomarkers. Whilst the sensitivity analysis stratified according to baseline eosinophil levels suggested an attenuation of the early and late asthmatic response in participants with cell counts of 100 cells/μL, participants with higher eosinophil levels (200 cells/μL) did not show an attenuation of early or late asthmatic response as would be expected if GSK2245035 had significant anti-T2 activity. Although fold changes in IP-10 in the present study were similar in magnitude to those observed in a previous study of GSK2245035 [13], demonstrating target engagement, no effect on suppression of the T2 response to allergen was observed.

The absence of an anti-T2 effect and impact on the late asthmatic response in this study contrast with those of previous studies with GSK2245035 in allergic rhinitis by Ellis et al. [13], and those by Leaker et al., assessing AZD8848 (another TLR7 agonist) in allergic asthma [20,24]. In the study by Ellis et al., a reduction of total nasal symptoms in response to nasal allergen challenge, and nasal allergic biomarker analyses, revealed trends supporting a response to GSK2245035, with reductions in T2-related cytokines [13]. Leaker et al., found that, compared with placebo, intranasal AZD8848 attenuated the late asthmatic response by approximately 27% at 1 week after dosing, but the results were not sustained at 4 weeks after dosing; however, AZD8848 had no significant effect on eosinophil counts or T2 cytokines [20,24]. TNSS score was evaluated daily, and although some nasal inflammatory mediators were impacted by GSK2245035, no clinically relevant treatment effect on TNSS was observed; however, this finding should be interpreted with caution given that a minimal TNSS score at study enrollment was not mandated (unlike the study by Ellis et al. [13]). Collectively, the present study and the study by Ellis et al. [13], suggest that TLR7 agonists administered via the intranasal route have some effect on allergen-induced T2 inflammation in the nose but minimal effect systemically, and do not substantially attenuate the late asthmatic response. The study was sufficiently sized to detect treatment effects of approximately 18% attenuation of the late asthmatic response (or larger); however, the observed data are consistent with there being no treatment effect.

The nasal allergen-induced release of tryptase, eotaxin, sIgA, and IFNγ with GSK2245035 compared with placebo are suggestive of a local GSK2245035-mediated treatment effect. However, although effective activation of T2 inflammatory responses to nasal allergen challenge were observed and target engagement confirmed, responses did not demonstrate the extent of evidence observed in the study by Ellis et al. [13]. This may be partly explained by the difference in nasal challenge technique (ie, bolus allergen doses in this study compared with symptomatic threshold doses in Ellis et al.) and the degree of participant eosinophil-dominated inflammation (ie, T2-driven allergic asthma phenotype) at baseline [25,26].

GSK2245035 20 ng administered intranasally once weekly for 8 weeks was well tolerated; the classification and frequency of adverse events was similar in the GSK2245035 and placebo groups. There was an excess of CRS-related adverse events in the GSK2245035 arm; however, these were mild or moderate in severity and self-limiting, and their incidence was similar to that previously reported at the 20 ng dose level [13]. The reporting of CRS-related adverse events of special interest is supportive of target engagement [13,27].

This study had several limitations. The relatively short dosing period of 8 weeks limited the ability to observe a potential treatment effect akin to that observed with subcutaneous allergen-specific immunotherapy, which, over a period of several months, modifies the T2 response in favor of type 1 T helper cell (Th1) and T regulatory cell generation [28]. However, there is no evidence to suggest that therapy with a TLR7 agonist for more extended periods and/or at higher doses may have greater impact on T2 inflammation, and previous data indicate that participants treated with doses of GSK2245035 greater than 20 ng report more CRS-related adverse events [13,27]. Additionally, due to early termination of the study, the number of participants generating secondary endpoint data was relatively low; therefore, drawing firm conclusions regarding these data are challenging. The proportion of female patients enrolled in the study was low, likely due to the exclusion criterion of women of childbearing potential. The previous study by Ellis et al. permitted the inclusion of women of childbearing potential, provided highly effective birth control was used [13], which may account for some of the differences between studies (due to extra precautions from GSK on the recruitment of women of childbearing potential since the Ellis study).

Another potential limitation was that some participants received their baseline bronchial allergen challenge outside of grass/birch pollen season and their post treatment bronchial allergen challenge within grass/birch pollen season; therefore, a seasonality effect caused by a variability in allergen exposure cannot be ruled out, and may have affected the treatment and allergen response. A potential limitation may be associated with polysensitization status, as participants who were polysensitized to multiple allergens were challenged with only a single selected allergen for all provocation challenges. It cannot be ruled out that a participant’s overall sensitization status may have impacted the treatment response; however, an associated strength is that the conditions throughout the study permitted natural exposure to allergen, potentially increasing the relevance of the results to the intended clinical setting.

These limitations may be compounded by the unequal treatment allocation in this study. The intended treatment allocation was 1:1; however, by a chance effect, this was closer to 2:1 (GSK2245035:placebo). A separate block of balanced randomization numbers was assigned to each combination of site and allergen exposure strata, but incomplete filling of these blocks with participants led to the chance overall imbalance when the data were combined for the analyses; different recruitment patterns and/or ordering of treatment assignment within each block would result in the expected 1:1 allocation if the study were repeated many times using the same randomization process. Compared to a 1:1 treatment allocation, such an imbalance between treatment arms may have affected the validity of results, although exposing a higher number of participants to treatment arguably may have beneficial effects, such as optimizing the collection of safety data by maximizing exposure to the treatment under study. Finally, it is worth noting that the recent update to the Global Initiative for Asthma guidelines recommend bronchodilator and inhaled corticosteroid as first-line asthma therapy [29]; therefore, the specific participant population used in this study is unlikely to be available in future clinical studies.

GSK2245035 20 ng administered intranasally once weekly over an 8-week treatment period did not substantially attenuate the early or late asthmatic response in participants with mild allergic asthma, and no changes in allergic reactivity was observed in the lower airways. Nasal and systemic TLR7 target engagement was observed and levels of IP-10 increased with treatment to the same magnitude observed in an earlier study. Repeated administration of GSK2245035 20 ng was well tolerated and had a similar adverse event profile to a previous study of the same agent in participants with allergic rhinitis, supporting its potential clinical utility in mild asthma. However, a treatment effect in the lung was not observed. Although no further studies are currently planned with GSK2245035, future development of a TLR7 modulator may be beneficial via an alternative route and or therapeutic indication, such as inhaled route for asthma and intranasal route for immunotherapy adjuvant.

## Supporting information

S1 Checklist CONSORT checklist. (DOC)

S1 Fig Mean total nasal symptom scores (all participants population). Mean total nasal symptom score weighted mean (all participants): A) nasal congestion; b) rhinorrhea; C) nasal itching; D) sneezing. If lower/upper limits for approximate 95% confidence interval are <0 or >3, respectively, they have been replaced with 0 or 3.
(DOCX)

S1 Table Sensitivity analyses. Summary of posterior distributions and posterior probability (PP) for minimum and weighted mean FEV_1_ change from baseline (following saline inhalation) at Week 9 using blood eosinophil levels as a covariate. CrI, credible interval; EAR, early asthmatic response; FEV1, forced expiratory volume in 1 second; LAR, late asthmatic response; PP, posterior probability; WM, weighted mean.
(DOCX)

S2 Table Nasal lavage and nasal filter eluate. Summary of nasal allergen challenge-associated fold change in biomarkers (GSK2245035/Placebo; adjusting for the screening visit fold changes) (all participants population). ^a^Refer to S6 File for source of analyte (ie, nasal lavage or nasal filter eluate). ↑, increase; ↓, decrease; CrI, credible interval; ECP, eosinophil cationic protein; IFN, interferon; IL, interleukin; FUV, follow-up visit; MDC, macrophage derived chemokine; PP, posterior probability; sIgA, allergen-specific immunoglobulin A; TARC, thymus and activation regulated chemokine.
(DOCX)

S1 File Key inclusion/exclusion criteria. (DOCX)

S2 File Incremental and bolus bronchial allergen challenge procedure. (DOCX)

S3 File Biomarker sampling schedule. (DOCX)

S4 File Biomarker assay details. (DOCX)

S5 File Study protocol. (PDF)

S6 File Details of independent ethics committees/institutional review boards. (DOCX)
