# Supplementary material for: Intranasal GSK2245035, a Toll-like receptor 7 agonist, does not attenuate the allergen-induced asthmatic response in a randomized, double-blind, placebo-controlled experimental medicine study
Source: PLoS One. 2020 Nov 9;15(11):e0240964. doi: 10.1371/journal.pone.0240964 (PMC7652256; doi:10.1371/journal.pone.0240964)
Supplement: S1 Fig — Mean total nasal symptom score weighted mean (all participants): A) nasal congestion; b) rhinorrhea; C) nasal itching; D) sneezing. If lower/upper limits for approximate 95% confidence interval are <0 or >3, respectively, they have been replaced with 0 or 3. (DOCX) [file pone.0240964.s002.docx]

## S1 Figure. Mean total nasal symptom scores. Mean total nasal symptom score weighted mean (all participants): A) nasal congestion; b) rhinorrhea; C) nasal itching; D) sneezing





If lower/upper limits for approximate 95% confidence interval are <0 or >3, respectively, they have been replaced with 0 or 3
