# Supplementary material for: Intranasal GSK2245035, a Toll-like receptor 7 agonist, does not attenuate the allergen-induced asthmatic response in a randomized, double-blind, placebo-controlled experimental medicine study
Source: PLoS One. 2020 Nov 9;15(11):e0240964. doi: 10.1371/journal.pone.0240964 (PMC7652256; doi:10.1371/journal.pone.0240964)
Supplement: S1 Table — Summary of posterior distributions and posterior probability (PP) for minimum and weighted mean FEV1 change from baseline (following saline inhalation) at Week 9 using blood eosinophil levels as a covariate. CrI, credible interval; EAR, early asthmatic response; FEV1, forced expiratory volume in 1 second; LAR, late asthmatic response; PP, posterior probability; WM, weighted mean. (DOCX) [file pone.0240964.s003.docx]

**S1 Table.** **Sensitivity analyses.** Summary of posterior distributions and posterior probability (PP) for minimum and weighted mean FEV_1_ change from baseline (following saline inhalation) at Week 9 using blood eosinophil levels as a covariate

| Baseline blood eosinophils | Endpoint | Percentage attenuation, % (95% CrI) | PP statement |
| --- | --- | --- | --- |
| 100 cells/µL | LAR WM FEV_1_ | 3.9 (–112.12, 55.17) | 0.547 |
|  | LAR min. FEV_1_ | 22.8 (–27.68, 55.99) | 0.854 |
| 200 cells/µL | LAR WM FEV_1_ | –5.7 (–73.93, 31.87) | 0.396 |
|  | LAR min. FEV_1_ | 1.0 (–40.34, 28.85) | 0.525 |
| 100 cells/µL | EAR WM FEV_1_ | 16.6 (–39.93, 51.40) | 0.773 |
|  | EAR min. FEV_1_ | 15.8 (–20.0, 41.44) | 0.842 |
| 200 cells/µL | EAR WM FEV_1_ | 6.0 (–35.38, 34.10) | 0.642 |
|  | EAR min. FEV_1_ | 4.8 (–21.37, 24.71) | 0.667 |
| CrI, credible interval; EAR, early asthmatic response; FEV_1,_ forced expiratory volume in 1 second; LAR, late asthmatic response; PP, posterior probability; WM, weighted mean | | | |
