# Supplementary material for: Intranasal GSK2245035, a Toll-like receptor 7 agonist, does not attenuate the allergen-induced asthmatic response in a randomized, double-blind, placebo-controlled experimental medicine study
Source: PLoS One. 2020 Nov 9;15(11):e0240964. doi: 10.1371/journal.pone.0240964 (PMC7652256; doi:10.1371/journal.pone.0240964)
Supplement: S2 Table — Summary of nasal allergen challenge-associated fold change in biomarkers (GSK2245035/Placebo; adjusting for the screening visit fold changes) (all participants population). aRefer to S6 File for source of analyte (ie, nasal lavage or nasal filter eluate). ↑, increase; ↓, decrease; CrI, credible interval; ECP, eosinophil cationic protein; IFN, interferon; IL, interleukin; FUV, follow-up visit; MDC, macrophage derived chemokine; PP, posterior probability; sIgA, allergen-specific immunoglobulin A; TARC, thymus and activation regulated chemokine. (DOCX) [file pone.0240964.s004.docx]

## S2 Table. Nasal lavage and nasal filter eluate. Summary of nasal allergen challenge-associated fold change in biomarkers (GSK2245035/Placebo; adjusting for the screening visit fold changes) (all participants population)

| **Analyte^a^** | **Time point** | **Nasal lavage fluid, median fold change (95% CrI)** | **PP distributions** |
| --- | --- | --- | --- |
| **sIgA** | FUV1 | 1.55 (0.65, 3.74) | 0.845 (Any ↑) |
|  | FUV2 | 1.39 (0.65, 2.94) | 0.808 (Any ↑) |
| **Histamine** | FUV1 | 1.13 (0.68, 1.89) | 0.312 (Any ↓) |
|  | FUV2 | 1.26 (0.65, 2.40) | 0.245 (Any ↓) |
| **Tryptase** | FUV1 | 0.28 (0.10, 0.78) | 0.991 (Any ↓) |
|  | FUV2 | 0.95 (0.30, 2.94) | 0.536 (Any ↓) |
| **ECP** | FUV1 | 2.48 (0.71, 9.28) | 0.076 (Any ↓) |
|  | FUV2 | 1.51 (0.64, 3.62) | 0.169 (Any ↓) |
| **IFNγ** | FUV1 | 2.11 (0.69, 6.47) | 0.910 (Any ↑) |
|  | FUV2 | 0.56 (0.18, 1.72) | 0.149 (Any ↑) |
| **IL-10** | FUV1 | 1.07 (0.31, 3.67) | 0.546 (Any ↑) |
|  | FUV2 | 0.94 (0.29, 3.13) | 0.463 (Any ↑) |
| **IL-5** | FUV1 | 1.51 (0.38, 6.24) | 0.273 (Any ↓) |
|  | FUV 2 | 1.30 (0.36, 4.68) | 0.341 (Any ↓) |
| **IL-13** | FUV1 | 0.85 (0.34, 2.17) | 0.635 (Any ↓) |
|  | FUV2 | 1.28 (0.40, 4.17) | 0.340 (Any ↓) |
| **IL-16** | FUV1 | 0.90 (0.24, 3.34) | 0.565 (Any ↓) |
|  | FUV2 | 1.17 (0.34, 4.12) | 0.399 (Any ↓) |
| **Eotaxin** | FUV1 | 0.54 (0.18, 1.63) | 0.867 (Any ↓) |
|  | FUV2 | 0.95 (0.33, 2.81) | 0.538 (Any ↓) |
| **MDC** | FUV1 | 1.10 (0.40, 2.92) | 0.423 (Any ↓) |
|  | FUV 2 | 2.17 (0.84, 5.59) | 0.530 (Any ↓) |
| **TARC** | FUV1 | 1.82 (0.61, 5.45) | 0.551 (Any ↓) |
|  | FUV2 | 2.54 (0.88, 7.56) | 0.043 (Any ↓) |
| ^a^Refer to S4 supporting information for source of analyte (ie, nasal lavage or nasal filter eluate).  ↑, increase; ↓, decrease; CrI, credible interval; ECP, eosinophil cationic protein; IFN, interferon; IL, interleukin;  FUV, follow-up visit; MDC, macrophage derived chemokine; PP, posterior probability; sIgA, allergen-specific immunoglobulin A; TARC, thymus and activation regulated chemokine | | | |
