# Supplementary material for: Intranasal GSK2245035, a Toll-like receptor 7 agonist, does not attenuate the allergen-induced asthmatic response in a randomized, double-blind, placebo-controlled experimental medicine study
Source: PLoS One. 2020 Nov 9;15(11):e0240964. doi: 10.1371/journal.pone.0240964 (PMC7652256; doi:10.1371/journal.pone.0240964)
Supplement: S1 File — (DOCX) [file pone.0240964.s005.docx]

## S1 Supporting information. Key study inclusion/exclusion criteria.

| Inclusion criteria | Aged 18 to 65 years, inclusive. |
| --- | --- |
|  | Body weight ≥45 kg. |
|  | Male or female of non-reproductive potential. |
|  | Diagnosis of asthma (as per GINA guidelines)[1] defined as a history of respiratory symptoms, such as wheeze, shortness of breath, chest tightness and cough that vary over time and in intensity, together with variable expiratory airflow limitation ≥6 months prior to screening. |
|  | Current asthma therapy with intermittent SABA alone (on average for no more than 2 days per week). SABA as a rescue medication was permitted, so long as it was withheld ≥6 hours prior to any study-specific lung function assessment or bronchial allergen challenge. |
|  | Positive skin prick test (wheal ≥3 mm or greater than the negative control) to common perennial or seasonal aeroallergen(s) (ie, house dust mite, cat dander, grass pollen) at screening. |
|  | Pre-bronchodilator FEV_1_ >70% predicted normal at Screening Visit 1. |
|  | Early asthmatic response with ≥20% FEV_1_ decrease between 5 and 30 min after the final allergen concentration was obtained at the screening bronchial allergen challenge (decreases relative to saline). |
|  | Late asthmatic response with three FEV_1_ decreases of ≥15% between 4 and 10 h after the final allergen concentration was obtained at the screening bronchial allergen challenge, with two FEV_1_ decreases being at consecutive time points (decreases relative to the saline). |
|  | Female: not pregnant (as confirmed by a negative [serum or urine] hCG test), not lactating, and where the following conditions apply:  Non-reproductive potential defined as:   - Pre-menopausal females with one of the following: - Documented tubal ligation - Documented hysteroscopic tubal occlusion procedure with follow-up confirmation of bilateral tubal occlusion - Hysterectomy - Documented bilateral oophorectomy - Postmenopausal defined as 12 months of spontaneous amenorrhea in questionable cases a blood sample with simultaneous FSH and estradiol levels consistent with menopause (refer to laboratory reference ranges for confirmatory levels)]. Females on HRT and whose menopausal status is in doubt will be required to use one of the highly effective contraception methods if they wish to continue their HRT during the study. Otherwise, they must discontinue HRT to allow confirmation of postmenopausal status prior to study enrollment. |
|  | Capable of giving signed informed consent which includes compliance with the requirements and restrictions listed in the consent form and study protocol. |
| Exclusion criteria | ALT >2 times the ULN and bilirubin >1.5 times the ULN (isolated bilirubin >1.5x ULN was acceptable if bilirubin was fractionated and direct bilirubin <35%). |
|  | Current or chronic history of liver disease or known hepatic or biliary abnormalities (with the exception of Gilbert's syndrome or asymptomatic gallstones). |
|  | Heart rate corrected QT interval (QTc) >450 msec or QTc >480 msec in subjects with Bundle Branch Block. |
|  | Asthma exacerbation requiring treatment with oral corticosteroids or hospitalization within 3 months prior to screening. |
|  | History of life-threatening asthma. |
|  | Evidence of concurrent respiratory diseases. |
|  | Other concurrent diseases/abnormalities. Participants must not have any clinically significant, uncontrolled condition or disease state that, in the opinion of the investigator would put the safety of the participant at risk through study participation or would confound the interpretation of the efficacy results if the condition/disease exacerbated during the study. |
|  | Respiratory tract infection that was not resolved within 2 weeks prior to screening. |
|  | Treatment with intranasal steroid, ICS with or without LABA and treatment with non-ICS controller asthma medications (ie, leukotriene modifier, methylxanthines) within 4 weeks prior to screening. |
|  | Treatment with systemic corticosteroids within 6 weeks prior to screening. |
|  | Use of long-acting antihistamines within 7 days or short-acting antihistamines within 72 hours prior to the screening skin prick test. |
|  | Use of inhaled SABAs as rescue treatment on average for more than 2 days per week. |
|  | Presence of HBsAg, positive hepatitis C antibody test result at screening or within 3 months prior to first dose of study treatment. |
|  | History of regular alcohol consumption within 6 months of the study defined as:  An average weekly intake of >14 units for males and females. One unit is equivalent to 8 g of alcohol: a half-pint (~240 ml) of beer, 1 glass (125 mL) of wine or 1 (25 mL) measure of spirits. |
|  | Participant known to be intolerant to salbutamol or albuterol. |
|  | History of sensitivity to any of the study medications, or components thereof or a history of drug or other allergy that, in the opinion of the investigator or GSK Medical Monitor, contraindicates their participation. |
|  | A positive pre-study drug/alcohol screen. |
|  | A positive test for HIV antibody. |
|  | Where participation in the study would result in donation of blood or blood products in excess of 500 mL within a 56-day period. |
|  | Participated in a clinical trial and has received an investigational product within the following time period prior to the first dosing day in the current study: 30 days, 5 half-lives or twice the duration of the biological effect of the investigational product (whichever is longer). |
|  | Exposure to more than 4 investigational medicinal products within 12 months prior to the first dosing day. |
| ALT, alanine transaminase; FEV_1_, forced expiratory volume in 1 second; FSH, follicle-stimulating hormone; GINA, Global Initiative for Asthma; GSK, GlaxoSmithKline; HBsAg, hepatitis B surface antigen; hCG, human chorionic gonadotrophin; HIV, human immunodeficiency virus; HRT, hormone replacement therapy; ICS, inhaled corticosteroid; LABA, long-acting β_2_-agonist; SABA, short-acting β_2_-agonist; ULN, upper limit of normal | |

**REFERENCES**

1. Global Initiative for Asthma (GINA). Global strategy for asthma management and prevention: Updated 2015; 2015 August 11 [cited 2019 August 21]. [Internet]. Available from: <https://ginasthma.org/wp-content/uploads/2016/01/GINA_Report_2015_Aug11-1.pdf>.
