# Supplementary material for: Intranasal GSK2245035, a Toll-like receptor 7 agonist, does not attenuate the allergen-induced asthmatic response in a randomized, double-blind, placebo-controlled experimental medicine study
Source: PLoS One. 2020 Nov 9;15(11):e0240964. doi: 10.1371/journal.pone.0240964 (PMC7652256; doi:10.1371/journal.pone.0240964)
Supplement: S2 File — (DOCX) [file pone.0240964.s006.docx]

## S2 Supporting information. Incremental and bolus bronchial allergen challenge procedure.

| Incremental bronchial allergen challenge procedure | |
| --- | --- |
| **Stage 1** | **Baseline spirometry** |
|  | 1. Three technically acceptable and reproducible recordings of FEV_1_ and FVC were performed. 2. The participant would not progress if the participant demonstrated an FEV_1_ ≤70% predicted (per Global Lung Initiative) [1] or manifested significant asthma symptoms of wheeze, chest tightness or cough. If the FEV_1_ criterion was not met in the first instance, a repeat baseline spirometry session could be performed after 20–30 minutes rest. |
| **Stage 2** | **Diluent challenge** |
|  | 1. Participants inhaled the number of breaths (3–5 breaths, as applicable) required to receive 45 μL (±10%) of diluent by inspiring slowly from FRC to TLC over 3 seconds, and then hold their breath for 6 seconds. 2. The dosimeter fired at the start of the inhalation, and lasted for the calibrated inhalation time. Time zero of the diluent challenge was when the participant exhaled the last breath. 3. At +2, +3, and +4 minutes, single measurements of FEV_1_ (only) were performed, and the **maximum** FEV_1_ value was taken as the post-diluent FEV_1_. 4. If any time pointed FEV_1_ effort was deemed to be technically incorrect or a more robust FEV_1_/higher measurement could be obtained (eg, such as due to insufficient inhalation, poor effort/technique), a repeat FEV_1_ value may be recorded after 30 seconds for that time point. 5. To determine the next step, the following rules applied:    1. If the highest post-diluent FEV_1_ was <10% from baseline spirometry FEV_1_, the participant progressed to allergen administration.    2. If the highest post-diluent FEV_1_ was ≥10% from baseline spirometry FEV_1_, the participant was required to rest for 20–30 minutes prior to restarting the procedure, commencing with a repeat baseline spirometry, which had to be ≥90% of the original baseline spirometry FEV_1_ and >70% of predicted FEV_1_. If the participant again demonstrated a fall in FEV_1_ of ≥10% following the repeat diluent challenge, then the participant was excluded/withdrawn. |
| **Stage 3** | **Incremental allergen administration^a^** |
|  | 1. Allergen administration always started with the lowest concentration (eg, Abelló ALK allergens started at 250 SQ-U/mL). The interval between subsequent increments of inhaled allergen was a minimum of 10 minutes. 2. The participant inhaled allergen over the required number of breaths (as applicable to the device used), in the same manner as the diluent challenge. Time zero of each allergen administration was when the participant exhaled the last breath. 3. At +5 and +10 minutes, single measurements of FEV_1_ (only) were performed, and the **minimum** FEV_1_ value was taken as the post-allergen FEV_1_. 4. If any time pointed FEV_1_ effort was deemed to be technically incorrect or a more robust FEV_1_/higher measurement could be obtained (eg, such as due to insufficient inhalation, poor effort/technique), a repeat FEV_1_ value may be recorded after 1-minute rest for that time point. If it was deemed possible that the participant could achieve a higher FEV_1_ (or the result is borderline, eg, 15.1% fall), the measurement was repeated. 5. To determine the next step in the incremental BAC, rules appropriate to the allergen used were applied. For example, the following rules were applied for ALK Abelló allergens:    1. If the post-allergen FEV_1_ was ≤10% from post-diluent FEV_1_, the participant inhaled a concentration 4 times higher (quadruple) next.    2. If the post-allergen FEV_1_ was >10% but <15% from post-diluent FEV_1_, the participant inhaled a concentration 2 times higher (double) next.    3. If the post-allergen FEV_1_ was ≥15% but <20% from post-diluent FEV_1_, the participant continued to *coasting* (see below).    4. If the post-allergen FEV_1_ was ≥20% from post-diluent FEV_1_, the participant met the early asthmatic response criteria and performed single FEV_1_ measurements at +15, +20, +30, and +45 minutes, then progressed to serial spirometry. 6. *Coasting was* performed when the inhaled allergen concentration nearly caused an early asthmatic response at either the +5- or +10-minute time points. Further single FEV_1_ measurements were performed at +15, +20, and +30 minutes to see if the FEV_1_ continued to fall past 20% from the post-diluent FEV_1_ and meet the early asthmatic response criteria:    1. If any of the *coasting* FEV_1_ values were ≥20% from post-diluent FEV_1_, the participant met the early asthmatic response criteria and performed a single FEV_1_ measurement at +45 minutes, then progressed to serial spirometry.    2. If any of the *coasting* FEV_1_ values were <20% from post-diluent FEV_1_, the participant inhaled the same concentration again.    3. Once a participant performed the *coasting* procedure, no further increments in allergen concentration were allowed, only the last concentration could be repeated (a maximum of twice, 3 doses in total). 7. Once the early asthmatic response had been achieved or it had been decided that no further allergen would be administered:    1. Single FEV_1_ measurements at +5, +10, +20, +30, and +45 minutes were performed (some of these measurements may have already been performed).    2. Then serial spirometry was performed from +1 hour onwards.    3. If the early asthmatic response criterion has not been achieved, the participant could be rescued following the +2-hour time point, and excluded. |
| **Stage 4** | **Serial spirometry** |
|  | 1. Duplicate FEV_1_ (only) measurements were performed 1 minute apart starting from +1 hour following allergen administration (ie, exhalation of last breath) every 30 minutes, up to +10 hours. The **maximum** FEV_1_ value of the duplicate measurements was taken as the FEV_1_ for that time point. 2. If any time pointed FEV_1_ effort was deemed to be technically incorrect (eg, such as due to insufficient inhalation), a repeat FEV_1_ value could be recorded after 1 minute of rest for that time point. 3. The late asthmatic response criterion was achieved when the FEV_1_ was ≥15% from post-diluent FEV_1_, at a minimum of three time points, two of which must be consecutive, between 4 to 10 hours post allergen administration. Following the +10-hour FEV_1_ measurements, the participant received rescue salbutamol until the FEV_1_ returned to within 10% of the baseline spirometry value (first of the day). If a participant experienced serious discomfort, the test was discontinued and rescue medication administered, at the discretion of the attending physician. |
| **Bolus bronchial allergen challenge procedure** | |
| **Stage 1** | **Baseline spirometry** |
|  | Performed according to the methods described above for the incremental bronchial allergen challenge procedure. |
| **Stage 2** | **Diluent challenge** |
|  | Performed according to the methods described above for the incremental bronchial allergen challenge procedure. |
| **Stage 3** | **Bolus allergen administration^a^** |
|  | 1. A prepared bolus dose of allergen equating to the cumulative concentration of all the doses administered during the incremental bronchial allergen challenge. 2. Once the bolus dose had been administered, no further allergen administration took place. 3. The participant inhaled the bolus allergen dose over 5 breaths in the same manner as the diluent challenge. 4. At +5, +10, +20, +30, and +45 minutes, single FEV_1_ (only) were performed. |
| **Stage 4** | **Serial spirometry** |
|  | Performed according to steps 1 and 2 in the methods described above for the incremental bronchial allergen challenge. |

^a^Allergens were provided by ALK Abelló [2] and Allegropharma [3]

BAC, bronchial allergen challenge; FEV_1_, forced expiratory volume in one second; FRC, functional residual capacity; FVC, forced vital capacity; TLC, total lung capacity

**REFERENCES**

1. Quanjer PH, Stanojevic S, Cole TJ, Baur X, Hall GL, Culver BH, et al. Multi-ethnic reference values for spirometry for the 3-95-yr age range: the global lung function 2012 equations. Eur Respir J. 2012;40:1324-1343.

2. ALK. ALK allergy solutions for life; 2018 [cited 2019 July]. [Internet]. Available from: https://www.alk.net/

3. Allegropharma. Holistic approach for allergy sufferers; 2019 [cited 2019 August 14]. [Internet]. Available from: <https://www.allergopharma.com/home/>
