# Supplementary material for: Intranasal GSK2245035, a Toll-like receptor 7 agonist, does not attenuate the allergen-induced asthmatic response in a randomized, double-blind, placebo-controlled experimental medicine study
Source: PLoS One. 2020 Nov 9;15(11):e0240964. doi: 10.1371/journal.pone.0240964 (PMC7652256; doi:10.1371/journal.pone.0240964)
Supplement: S3 File — (DOCX) [file pone.0240964.s007.docx]

## S3 Supporting information. Biomarker sampling schedule.

| Sample | Time point | | |
| --- | --- | --- | --- |
|  | **Screening visits** | **Dosing visits** | **Follow-up visits** |
| Induced sputum | SV1  SV2+24 h | DV8**^a^** | FUV1+24 h**^b^**  FUV2 and FUV2+24 h**^b^**  FUV3 and FUV3+24 h**^b^** |
| Blood samples for hematology | SV2^b^ | DV1**^c^**  DV8**^c^** | FUV1, 2 and 3^b^ |
| Blood samples (serum) | Not performed | DV1^c,d^  DV8^c,d^ | Not performed |
| Nasal lavage | SV2+24 h**^e^** | DV1**^c,d^**  DV4**^c,d^**  DV8**^c,d^** | FUV1 + 24 h**^e^**  FUV2 + 24 h**^e^** |
| FeNO | SV1  SV2+24 h**^f^** | DV1–8**^g^** | FUV1, 2 and 3**^f^**  24 h post FUV 1, 2 and 3**^f^** |
| Intradermal challenge^h,i^ | SV2+24 h | Not performed | FUV1+24 h  FUV2+24 h |
| Nasal scrape^i,j^ | SV2+24 h | Not performed | FUV1  FUV2 |
| ^a^Pre-BAC sample for FUV1  ^b^pre- and 24 h post BAC  ^c^pre-dose and 24 h post dose  ^d^pharmacodynamic and IP-10 measurements  ^e^pre-NAC, 5 mins post NAC and ≥6 h post NAC  ^f^pre-sputum induction  ^g^pre-dose  ^h^post NAC  ^i^insufficient samples to permit data review  ^j^≥6 h post NAC  BAC, bronchial allergen challenge; DV, dosing visit; FeNO, fractional exhaled nitric oxide; FUV, follow-up visit; NAC, nasal allergen challenge; SV, screening visit | | | |
