# Supplementary material for: Intranasal GSK2245035, a Toll-like receptor 7 agonist, does not attenuate the allergen-induced asthmatic response in a randomized, double-blind, placebo-controlled experimental medicine study
Source: PLoS One. 2020 Nov 9;15(11):e0240964. doi: 10.1371/journal.pone.0240964 (PMC7652256; doi:10.1371/journal.pone.0240964)
Supplement: S4 File — (DOCX) [file pone.0240964.s008.docx]

## S4 Supporting information. Biomarker assay details.

| Sample | Biomarker | LLQ | Unit |
| --- | --- | --- | --- |
| Pharmacodynamic biomarker assays | | |  |
| Serum^a^ | IP-10 | 2.32 | ng/L |
|  | MCP-1 | 7.80 | ng/L |
|  | IFN-α | 12.5 | ng/L |
|  | IFN-β | 1.17 | ng/L |
|  | IL-1β | 1.08 | ng/L |
|  | IL-6 | 1.62 | ng/L |
|  | TNF-α | 0.68 | ng/L |
| Nasal lavage^b^ | IP-10 | 1.95 | ng/mL |
| T2 inflammatory mediators | | |  |
| Sputum | IL-5^a^ | 0.19 | ng/L |
|  | IL-13^a^ | 2.22 | ng/L |
| Nasal filter eluate^d^ | IL-5 | 0.22 | ng/mL |
|  | IL-10 | 0.09 | ng/mL |
|  | IL-13 | 4.21 | ng/mL |
|  | IL-16 | 0.83 | ng/mL |
|  | IFN-γ | 0.33 | ng/mL |
|  | TARC | 0.46 | ng/mL |
|  | Eotaxin | 7.11 | ng/mL |
|  | MDC | 2.81 | ng/mL |
| Nasal lavage | sIgA^e^ | 0.000625 | µg/mL |
|  | ECP^e^ | 0.125 | µg/mL |
|  | Tryptase^e^ | 0.0625 | µg/mL |
|  | Histamine^f^ | 0.391 | µg/mL |
| ^a^Analyzed by Q Squared Solutions (Quest) [1]  ^b^Measured at GSK using R&D Systemshuman IP-10 ELISA [2]  ^c^Analyzed using cytospins at the US Environmental Protection Agency (EPA) Human Studies Facility, University of North Carolina [3]  ^d^Assayed at GSK using MSD V-Plex Th1/Th2 panel assays [4]  ^e^Analyzed using Phadia ImmunoCAP [5]  ^f^Analyzed by ELISA (Enzo) [6]  ECP, eosinophil cationic protein; ELISA, enzyme-linked immunosorbent assay; GSK, GlaxoSmithKline; IFN, interferon; IL, interleukin; IP-10, interferon inducible protein-10; LLQ, lower limit of quantification; MCP-1, macrophage chemoattractant protein-1; MDC, macrophage derived chemokine; sIgA, allergen-specific immunoglobulin A; TARC, thymus and activation-regulated chemokine; Th1, type 1 T-helper cell; Th2, type 2 T-helper cell; TNF, tumor necrosis factor | | | |

**REFERENCES**

1. Q^2^ Solutions. Q^2^ Lab Solutions; 2019 [cited 2019 July 22]. [Internet]. Available from: https://www.q2labsolutions.com/

2. R & D Systems. R&D systems a biotechne brand; 2019 [cited 2019 July 22]. [Internet]. Available from: <https://www.rndsystems.com/>

3. UNC School of Medicine. Center for Environmental Medicine, Asthma and Lung Biology; 2019 [cited 2019 July 22]. [Internet]. Available from: <https://www.med.unc.edu/cemalb/about-the-center/>

4. MSD. MSD Multi-Spot Assay System: Proinflammatory Panel 1 (human) kits; 2018 [cited 2019 July 22]. [Internet].  https://www.mesoscale.com/~/media/files/product%20inserts/proinflammatory%20panel%201%20human%20insert.pdf

5. Thermo Scientific. ImmunoCAP Lab Tests; 2012 [cited 2019 July 22]. In: Phadia.com [Internet]. <http://www.phadia.com/Products/Allergy-testing-products/ImmunoCAP-Lab-Tests/>

6. Enzo. Immunoassays ELISA Kits; 2019 [cited 2019 July 22]. In: EnzoLifeSciences.com [Internet]. http://www.enzolifesciences.com/platforms/immunoassay-and-assay-development/immunoassays-elisa-kits/
