# Supplementary material for: Intranasal GSK2245035, a Toll-like receptor 7 agonist, does not attenuate the allergen-induced asthmatic response in a randomized, double-blind, placebo-controlled experimental medicine study
Source: PLoS One. 2020 Nov 9;15(11):e0240964. doi: 10.1371/journal.pone.0240964 (PMC7652256; doi:10.1371/journal.pone.0240964)
Supplement: S5 File — (PDF) [file pone.0240964.s009.pdf]

TITLE PAGE

Division: Worldwide Development
Information Type: Protocol Amendment

**Title:**	A randomised, double-blind (sponsor open) placebo-controlled, parallel group, 8-week treatment study to investigate the safety, pharmacodynamics, and effect of the TLR7 agonist, GSK2245035, on the allergen-induced asthmatic response in subjects with mild allergic asthma

Compound Number: GSK2245035

Development Phase: II

Effective Date: 17-AUG-2016

Protocol Amendment Number: 02

Author (s):

PPD

Revision Chronology

<table>
<tr><th>GlaxoSmithKline Document Number</th><th>Date</th><th>Version</th></tr>
<tr><td>2015N264314_00</td><td>2016-APR-06</td><td>Original</td></tr>
<tr><td>2015N264314_01</td><td>2016-JUN-15</td><td>Amendment No. 1</td></tr>
<tr><td colspan="3">Section 4.6.1 and Section 7.1.1 have been adjusted slightly to make it clearer that subjects will be provided with a thermometer to be able to record temperature as part of capturing adverse event (AE) information.

In Section 5.2, exclusion criterion 13 has been updated to reflect the revised recommended alcohol consumption guidelines.</td></tr>
<tr><td>2015N264314_02</td><td>2016-AUG-17</td><td>Amendment No. 2</td></tr>
<tr><td colspan="3">The protocol title and text have been amended to clarify that while the investigator and subjects are blinded the sponsor-is unblinded.

Details of the procedures required at early withdrawal visits have been added to the Time and Events Table, Section 7.1.

Details around the Safety Review Team have been added to include a consultant external to GSK to Section 6.3.

The inclusion and exclusion criteria have been updated in Section 5.1 and Section 5.2. and clarification around prohibited medications has been provided in Section 6.11.2. to align with updated exclusion criteria (Section 5.2).

Administrative changes have been made to the T&E table (Section 7.1) and text to ensure consistency throughout.</td></tr>
</table>

SPONSOR SIGNATORY

PPD

17 Aug 2016

Laurie A. Lee,
Physician Project Lead/EDL
Respiratory R&D

PPD

MEDICAL MONITOR/SPONSOR INFORMATION PAGE

Medical Monitor/SAE Contact Information:

**Role**	Name	Day Time Phone Number and email address	After-hours Phone/Cell/ Pager Number	Fax Number	Site Address
Primary Medical Monitor	PPD MD	PPD			5 Moore Drive, PO Box 13398, RTP, NC 27709-3398, United States
Secondary Medical Monitor	PPD PPD MD				Gunnels Wood Road Stevenage, SG1 2NY UK
SAE contact information	Medical monitor as above and Study Manager, PPD				Gunnels Wood Road Stevenage, SG1 2NY UK

Sponsor Legal Registered Address:

GlaxoSmithKline Research & Development Limited
980 Great West Road
Brentford
Middlesex, TW8 9GS
UK

In some countries, the clinical trial sponsor may be the local GlaxoSmithKline Affiliate Company (or designee). If applicable, the details of the alternative Sponsor and contact person in the territory will be provided to the relevant regulatory authority as part of the clinical trial application.

Regulatory Agency Identifying Number(s): 2015-005645-31

INVESTIGATOR PROTOCOL AGREEMENT PAGE

For protocol number 205540:

I confirm agreement to conduct the study in compliance with the protocol, as amended by this protocol amendment.

I acknowledge that I am responsible for overall study conduct. I agree to personally conduct or supervise the described study.

I agree to ensure that all associates, colleagues and employees assisting in the conduct of the study are informed about their obligations. Mechanisms are in place to ensure that site staff receives the appropriate information throughout the study.

Investigator Name:	
Investigator Address:	
Investigator Phone Number:	
Investigator Signature	Date

TABLE OF CONTENTS

PAGE

1. PROTOCOL SYNOPSIS FOR STUDY 205540 ... 9

2. INTRODUCTION ... 12
2.1. Study Rationale/Brief background ... 12

3. OBJECTIVE(S) AND ENDPOINT(S) ... 13

4. STUDY DESIGN ... 15
4.1. Overall Design ... 15
4.2. Treatment Arms and Duration ... 15
4.3. Type and Number of Subjects ... 15
4.4. Design Justification ... 16
4.5. Dose Justification ... 17
4.6. Benefit:Risk Assessment ... 18
4.6.1. Risk Assessment ... 19
4.6.2. Benefit Assessment ... 25
4.6.3. Overall Benefit:Risk Conclusion ... 25

5. SELECTION OF STUDY POPULATION AND WITHDRAWAL CRITERIA ... 25
5.1. Inclusion Criteria ... 25
5.2. Exclusion Criteria ... 28
5.3. Screening Failures/Re-screening ... 30
5.4. Withdrawal/Stopping Criteria ... 30
5.4.1. Liver Chemistry Stopping Criteria ... 31
5.4.1.1. Study Treatment Restart or Rechallenge ... 31
5.4.2. QTc Stopping Criteria ... 31
5.5. Subject and Study Completion ... 32

6. STUDY TREATMENT ... 32
6.1. Investigational Product and Other Study Treatment ... 32
6.2. Treatment Assignment ... 33
6.3. Blinding ... 34
6.4. Packaging and Labeling ... 35
6.5. Preparation/Handling/Storage/Accountability ... 35
6.6. Compliance with Study Treatment Administration ... 36
6.7. Treatment of Study Treatment Overdose ... 36
6.8. Treatment after the End of the Study ... 36
6.9. Challenge Agents ... 36
6.9.1. Bronchial, intranasal and intradermal allergen challenge/administration ... 36
6.10. Lifestyle and/or Dietary Restrictions ... 37
6.10.1. Meals and Dietary Restrictions ... 37
6.10.2. Caffeine, Alcohol, and Tobacco ... 37
6.10.3. Activity ... 37
6.11. Concomitant Medications and Non-Drug Therapies ... 37
6.11.1. Permitted Medications and Non-Drug Therapies ... 37
6.11.2. Prohibited Medications and Non-Drug Therapies ... 37

7. STUDY ASSESSMENTS AND PROCEDURES ... 38
7.1. Time and Events Tables ... 40

7.1.1. Time and Events Table – Screening Visit(s) ... 40
7.1.2. Time and Events Table – Dosing visit (DV) 1 (Day 1) – DV8 (Week 8) and Early Withdrawal Visit (EW) ... 42
7.1.3. Time and Events Table – Follow up visits (FUV1 to 3) (Weeks 9 to 20) ... 44
7.2. Screening and Critical Baseline Assessments ... 46
7.2.1. Skin prick testing ... 46
7.3. Efficacy ... 46
7.3.1. Bronchial allergen challenges and spirometry ... 46
7.3.2. Intranasal and intradermal allergen challenge ... 46
7.3.3. Sputum induction ... 46
7.3.4. TNSS ... 47
7.3.5. Nasal examination ... 47
7.3.6. Fractional Exhaled Nitric Oxide (FeNO) ... 47
7.4. Safety ... 47
7.4.1. Adverse Events (AE) and Serious Adverse Events (SAEs) ... 48
7.4.1.1. Time period and Frequency for collecting AE and SAE information ... 48
7.4.1.2. Method of Detecting AEs and SAEs ... 49
7.4.1.3. Follow-up of AEs and SAEs ... 49
7.4.1.4. Cardiovascular and Death Events ... 49
7.4.1.5. Disease-Related Events and/or Disease-Related Outcomes Not Qualifying as SAEs ... 50
7.4.1.6. Regulatory Reporting Requirements for SAEs ... 50
7.4.1.7. Cytokine Related Symptoms (CRS) - Symptoms Grading ... 51
7.4.2. Pregnancy ... 51
7.4.3. Physical Exams ... 51
7.4.4. Vital Signs ... 51
7.4.5. Electrocardiogram (ECG) ... 51
7.4.6. Clinical Safety Laboratory Assessments ... 52
7.4.7. PEF monitoring ... 54
7.5. Pharmacokinetics ... 54
7.5.1. Blood Sample Collection ... 54
7.5.2. Sample Analysis ... 54
7.6. Exploratory Biomarkers ... 54
7.6.1. Pharmacodynamic Markers ... 54
7.6.1.1. PD blood samples ... 54
7.6.1.2. Blood PAX collection ... 55
7.6.1.3. PD nasal lavage samples ... 55
7.6.1.4. Bronchial allergen challenge (BAC) associated biomarkers in induced sputum samples ... 55
7.6.1.5. Bronchial allergen challenge (BAC) associated biomarkers in blood samples ... 55
7.6.1.6. Nasal allergen challenge associated biomarkers in nasal fluid samples ... 55
7.6.1.7. Cellular profile of nasal mucosa ... 55
7.6.2. Novel Biomarkers ... 56
7.7. Genetics ... 56

8. DATA MANAGEMENT ... 56

9. STATISTICAL CONSIDERATIONS AND DATA ANALYSES ... 57
9.1. Hypotheses ... 57
9.2. Sample Size Considerations ... 57
9.2.1. Sample Size Assumptions ... 61
9.2.2. Sample Size Sensitivity ... 62
9.2.3. Sample Size Re-estimation or Adjustment ... 62
9.3. Data Analysis Considerations ... 62
9.3.1. Analysis Populations ... 62
9.3.2. Interim Analysis ... 63
9.3.2.1. Headline results for LAR endpoints at Follow Up 1 ... 65
9.4. Key Elements of Analysis Plan ... 66
9.4.1. Primary Analyses ... 66
9.4.2. Secondary Analyses ... 66
9.4.3. Safety Analyses ... 66
9.4.4. Exploratory Analyses ... 66

10. STUDY GOVERNANCE CONSIDERATIONS ... 66
10.1. Posting of Information on Publicly Available Clinical Trial Registers ... 66
10.2. Regulatory and Ethical Considerations, Including the Informed Consent Process ... 67
10.3. Quality Control (Study Monitoring) ... 67
10.4. Quality Assurance ... 68
10.5. Study and Site Closure ... 68
10.6. Records Retention ... 68
10.7. Provision of Study Results to Investigators, Posting of Information on Publically Available Clinical Trials Registers and Publication ... 69

11. REFERENCES ... 70

12. APPENDICES ... 71
12.1. Appendix 1 – Abbreviations and Trademarks ... 71
12.2. Appendix 2: Liver Safety Required Actions and Follow up Assessments ... 74
12.3. Appendix 3 - Genetic Research ... 76
12.4. Appendix 4: Definition of and Procedures for Recording, Evaluating, Follow-Up and Reporting of Adverse Events ... 79
12.4.1. Definition of Adverse Events ... 79
12.4.2. Definition and Grading of CRS-related Adverse Events ... 81
12.4.3. Definition of Serious Adverse Events ... 82
12.4.4. Definition of Cardiovascular Events ... 84
12.4.5. Recording of AEs and SAEs ... 84
12.4.6. Evaluating AEs and SAEs ... 85
12.4.7. Reporting of SAEs to GSK ... 86
12.5. Appendix 5: Modified List of Highly Effective Methods for Avoiding Pregnancy and Collection of Pregnancy Information ... 87
12.5.1. Modified List of Highly Effective Methods for Avoiding Pregnancy ... 87
12.5.2. Collection of Pregnancy Information ... 87
12.5.3. References ... 88
12.6. Appendix 6 - Country Specific Requirements ... 90
12.7. Appendix 7: Protocol Amendment Changes ... 91

1. PROTOCOL SYNOPSIS FOR STUDY 205540

Rationale

GSK2245035 is a highly selective Toll-like receptor 7 (TLR7) agonist that preferentially stimulates the induction of type I interferons. Intranasal (i.n.) administration of GSK2245035 in humans has been shown to cause immune changes in the upper airways milieu that may alter bystander immune responsiveness to aeroallergens and contribute to reduction of allergic reactivity in subjects with respiratory allergies.

In a 8-week repeat dose safety study (TL7116958) with i.n. GSK2245035 20 ng and 80 ng, target engagement was confirmed by dose-related increases in serum and nasal lavage interferon inducible protein-10 (IP-10) levels in subjects with symptomatic allergic rhinitis and mild asthma. Prolonged activation (up to 1 week in the serum and 3 weeks in nasal lavage) of the interferon (IFN) α pathway was noted at the higher dose with no associated adverse effects during this follow-up period. Eight weekly doses of both 20 ng and 80 ng reduced nasal symptoms and allergic biomarkers after nasal allergen challenge and those reductions were sustained up to 3 weeks post treatment.

This study will assess whether TLR7-mediated pharmacology, with i.n. GSK2245035 20 ng administered weekly for a period of 8 weeks, will lead to reduced allergic reactivity in the lower airways in subjects with mild allergic asthma.

Objective(s)/Endpoint(s)

Objectives	Endpoints
**Primary**	
• To evaluate the effect of treatment with i.n. GSK2245035 compared to placebo on the allergen-induced late asthmatic response (LAR) in subjects with allergic asthma.	• LAR: minimum forced expiratory volume in 1 second (FEV_1) between 4-10 hours following allergen challenge one week after treatment.<br>• LAR: weighted mean FEV_1 between 4-10 hours following allergen challenge one week after treatment.
**Secondary**	
To evaluate the effect of treatment with i.n. GSK2245035 compared to placebo on the allergen-induced early asthmatic response (EAR) in subjects with allergic asthma.	EAR: minimum FEV_1 between 0-2 hours following allergen challenge one week after treatment.<br>EAR: weighted mean FEV_1 between 0-2 hours following allergen challenge one week after treatment.

Objectives	Endpoints
**Safety**	
To evaluate the safety and tolerability of treatment with eight weekly doses of 20 ng i.n. GSK2245035	General safety endpoints including adverse events (AEs), peak expiratory flow (PEF), rescue salbutamol use, and clinical laboratory parameters
**Exploratory**	
To evaluate the duration of effect of treatment with i.n. GSK2245035 compared to placebo on the allergen-induced asthmatic responses (LAR and EAR) in subjects with allergic asthma.	LAR: minimum and weighted mean FEV_1 between 4-10 hours following allergen challenge approximately 4 and 12 weeks after treatment.<br>EAR: minimum and weighted mean FEV_1 between 0-2 hours following allergen challenge approximately 4 and 12 weeks after treatment.
To evaluate the induction of TLR7-associated pharmacodynamic (PD) biomarkers and exhaled nitric oxide (FeNO) following treatment with i.n. GSK2245035	TLR7-induced blood PD biomarkers.<br>TLR7 induced gene expression changes in blood<br>TLR7-induced nasal fluid PD biomarkers<br>Change in FeNO levels during the treatment phase
To evaluate the effect of treatment with i.n. GSK2245035 compared to placebo on Bronchial Allergen Challenge (BAC) associated allergic biomarkers	Allergic biomarkers in induced sputum<br>Allergic biomarkers in blood<br>Change in FeNO levels following BAC
To evaluate the effect of treatment with i.n. GSK2245035 compared to placebo on Nasal Allergen Challenge(NAC) associated allergic biomarkers	Allergic biomarkers in nasal fluids<br>Cellular profile of nasal mucosa
To evaluate the effect of treatment with i.n. GSK2245035 compared to placebo on Intradermal allergen challenge	Mean diameter early phase response<br>Mean diameter late phase response
To evaluate the effect of treatment with i.n. GSK2245035 compared to placebo on total nasal symptoms	Total nasal symptoms score (TNSS) assessed daily (AM and PM) throughout the study treatment phase
To evaluate systemic pharmacokinetics (PK) after administration of i.n. GSK2245035	Plasma GSK2245035 concentrations

Overall Design

This will be a randomised, double-blind (sponsor open), placebo-controlled, parallel group, 8-week treatment study. Study schematic below:

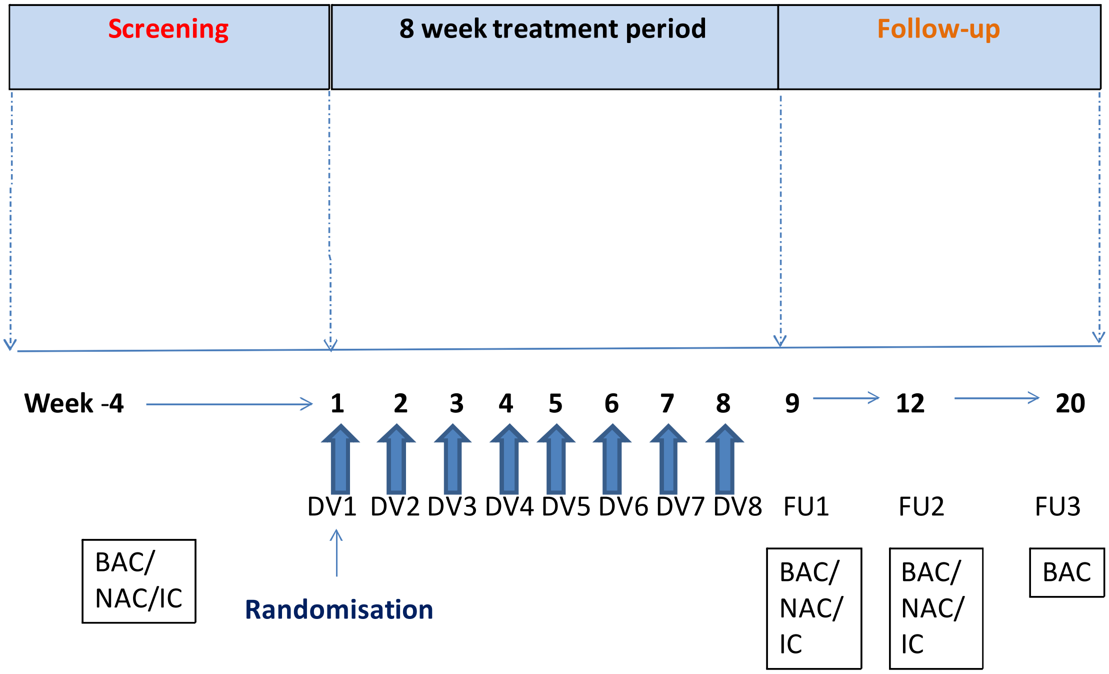

BAC -Bronchial allergen challenge; NAC – Nasal allergen challenge/administration
IC – Intradermal challenge; DV – Dosing Visit; FU – Follow-up

Treatment Arms and Duration

- The study will consist of a screening period of up to approximately 4 weeks (involving two screening visits), a blinded treatment period of 8 weeks, followed by a follow-up period of up to 3 months. The total duration of the study for each subject will therefore be a maximum of approximately 6 months. All doses will be administered in the clinic.
- Approximately 24 subjects per arm will be randomised in a 1:1 ratio to receive either i.n. 20ng GSK2245035 or placebo every week for the duration of 8 weeks.

Type and Number of Subjects

- Subjects with allergic asthma who are sensitized to perennial or seasonal allergen(s) will be enrolled in the study. Based on the restriction of concomitant medications as shown in the exclusion criteria, the target population includes subjects with mild asthma not requiring maintenance treatment with inhaled corticosteroid who are able to be treated with short acting beta$_2$-agonist (SABA) alone for the duration of the study (up until the final follow-up visit).

- A sufficient number of subjects will be enrolled to ensure that 20 per treatment arm complete the 8 week study treatment period and the BAC at the one week follow-up visit (one week after the 8th dose of study treatment).

Analysis

Statistical models will be fitted separately to each primary endpoint. These endpoints will be analysed using analysis of covariance (ANCOVA) with treatment and baseline in the model; other covariates such as centre, allergen exposure (‘presumed’/’unknown’) and allergen exposure by treatment interaction may be included in the model.

Treatment comparisons of GSK2245035 vs Placebo will be presented with results also expressed as percentage attenuations to aid clinical interpretation. The posterior distribution for GSK2245035 vs Placebo will be obtained and expressed as percentage attenuation. The posterior probability of any percentage attenuation will be obtained, along with appropriate summary statistics from the posterior distribution (e.g. 70% equi-tail Credible Intervals).

The principle inferences are expected to come from models with non-informative priors. Sensitivity analyses using informative priors based on AstraZeneca (AZ) data may be fitted [Leaker, 2012].

An interim analysis will be conducted to assess how many subjects on placebo did not experience an LAR as such subjects will reduce the chances of establishing the primary objective of this study. This interim analysis will determine whether i) further recruitment into the trial should continue (i.e. a futility analysis), or ii) additional subjects should be enrolled to counteract the impact.

2. INTRODUCTION

2.1. Study Rationale/Brief background

The immunological milieu of the airways plays a critical role in shaping the nature of host responsiveness to inhaled allergens with predominant induction of aberrant Th2 responses driving the development of respiratory allergies and asthma. The scientific hypothesis is that intranasal delivery of a TLR7 agonist will activate local dendritic cells, inducing a type 1 IFN response, and result in the modification of the nature of both B and T cell responses to allergen. Driving the development of Th1/Treg responses may reduce the magnitude of mucosal Th2 reactivity and reduce local IgE responses.

GSK2245035 is a highly selective Toll-like receptor 7 (TLR7) agonist that stimulates preferentially the induction of type I interferons. In a 8-week repeat dose safety study (TL7116958) [GlaxoSmithKline Document Number 2013N174200_00] with i.n. GSK2245035 20ng and 80 ng, target engagement was confirmed by dose-related increases in serum and nasal lavage IP-10, (interferon inducible protein 10) levels in subjects with symptomatic allergic rhinitis and mild asthma. Prolonged activation (up to 1 week in the serum and 3 weeks in nasal lavage) of the IFNα pathway was noted at the higher dose with no associated adverse effects during this follow-up period. Eight weekly doses of both 20 ng and 80 ng reduced nasal symptoms and allergic biomarkers

after nasal allergen challenge and those reductions were sustained up to 3 weeks post treatment.

Having demonstrated the effects i.n. GSK2245035 in patients with allergic rhinitis, this study will assess whether TLR7-mediated pharmacology, with i.n. GSK2245035 20 ng administered weekly for a period of 8 weeks, will lead to reduced allergic reactivity in the lower airways in subjects with mild allergic asthma using a bronchial allergen challenge model (BAC) at various time-points following treatment. For asthma patients who have allergic triggers for their disease, an i.n. treatment may be considered ideal to maximize co-localization with both its target, local dendritic cells, and aeroallergen. Clinical evidence to support the hypothesis that an intranasal TLR7 agonist can reduce the response to allergen in the lower airways using a BAC model has been reported (Leaker, 2012).

3. OBJECTIVE(S) AND ENDPOINT(S)

Objective(s)/Endpoint(s)

Objectives	Endpoints
**Primary**	
• To evaluate the effect of treatment with i.n. GSK2245035 compared to placebo on the allergen-induced late asthmatic response (LAR) in subjects with allergic asthma.	LAR: minimum FEV1 between 4-10 hours following allergen challenge one week after treatment.<br>LAR: weighted mean FEV1 between 4-10 hours following allergen challenge one week after treatment.
**Secondary**	
To evaluate the effect of treatment with i.n. GSK2245035 compared to placebo on the allergen-induced early asthmatic response (EAR) in subjects with allergic asthma.	EAR: minimum FEV1 between 0-2 hours following allergen challenge one week after treatment.<br>EAR: weighted mean FEV_1 between 0-2 hours following allergen challenge one week after treatment.
**Safety**	
To evaluate the safety and tolerability of treatment with eight weekly doses of 20 ng i.n. GSK2245035	General safety endpoints including AE, peak expiratory flow (PEF), rescue salbutamol use, and clinical laboratory parameters

Objectives	Endpoints
**Exploratory**	
To evaluate the duration of effect of treatment with i.n. GSK2245035 compared to placebo on the allergen-induced asthmatic responses (LAR and EAR) in subjects with allergic asthma.	LAR: minimum and weighted mean FEV_1 between 4-10 hours following allergen challenge approximately 4 and 12 weeks after treatment.<br>EAR: minimum and weighted mean FEV_1 between 0-2 hours following allergen challenge approximately 4 and 12 weeks after treatment.
To evaluate the induction of TLR7-associated PD biomarkers and FeNO following treatment with i.n. GSK2245035	TLR7-induced blood PD biomarkers.<br>TLR7 induced gene expression changes in blood<br>TLR7-induced nasal fluid PD biomarkers<br>Change in FeNO levels during the treatment period
To evaluate the effect of treatment with i.n. GSK2245035 compared to placebo on Bronchial Allergen Challenge (BAC) associated allergic biomarkers	Allergic biomarkers in induced sputum<br>Allergic biomarkers in blood<br>Change in FeNO levels following BAC
To evaluate the effect of treatment with i.n. GSK2245035 compared to placebo on Nasal Allergen Challenge (NAC) associated allergic biomarkers	Allergic biomarkers in nasal fluids<br>Cellular profile of nasal mucosa
To evaluate the effect of treatment with i.n. GSK2245035 compared to placebo on Intradermal allergen challenge	Mean diameter early phase response<br>Mean diameter late phase response
To evaluate the effect of treatment with i.n. GSK2245035 compared to placebo on total nasal symptoms	Total nasal symptoms score (TNSS) assessed daily (AM and PM) throughout the study treatment phase
To evaluate systemic pharmacokinetics (PK) after administration of i.n. GSK2245035	Plasma GSK2245035 concentrations

4. STUDY DESIGN

4.1. Overall Design

This will be a randomised, double-blind (sponsor open), placebo-controlled, parallel group, 8-week treatment study. Study schematic below:

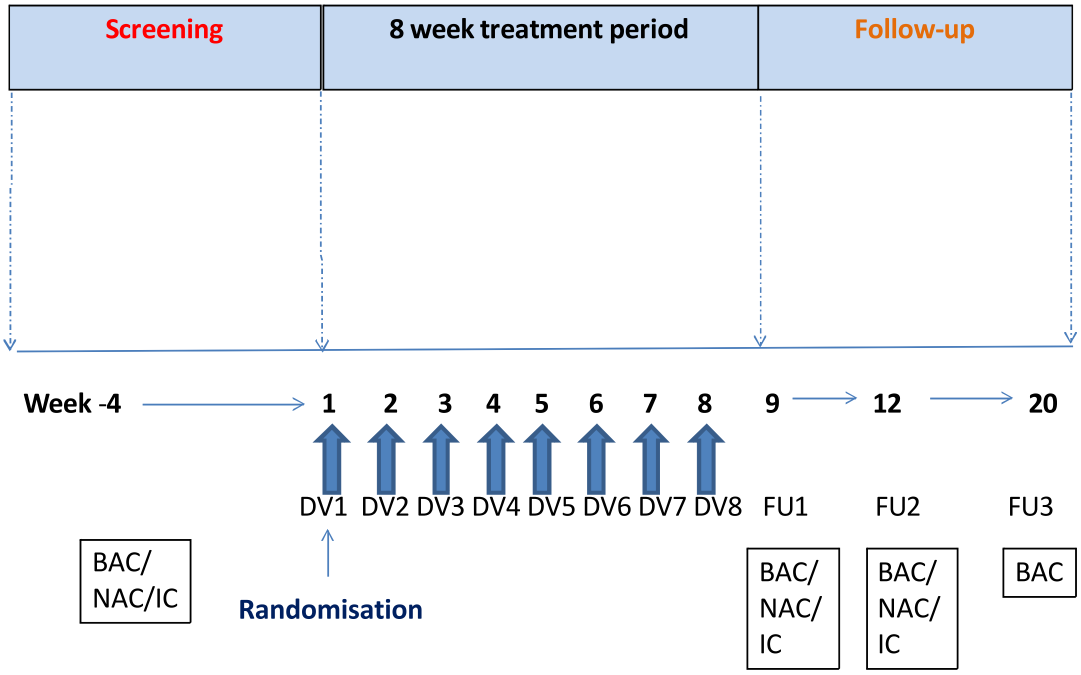

BAC -Bronchial allergen challenge; NAC – Nasal allergen challenge/administration
IC – Intradermal challenge; DV – Dosing Visit; FU – Follow-up

4.2. Treatment Arms and Duration

- The study will consist of a screening period of up to approximately 4 weeks (involving two screening visits), a blinded treatment period of 8 weeks, followed by a follow-up period of up to 3 months. The total duration of the study for each subject will therefore be a maximum of approximately 6 months. All doses will be administered in the clinic.
- Approximately 24 subjects per treatment arm will be randomised in a 1:1 ratio to receive either i.n. 20ng GSK2245035 or placebo every week for the duration of 8 weeks.

4.3. Type and Number of Subjects

- Subjects with allergic asthma who are sensitized to perennial or seasonal allergen(s) will be enrolled in the study. Based on the restriction of concomitant medications as shown in the exclusion criteria, the target population includes subjects with mild asthma not requiring maintenance treatment with inhaled corticosteroid who are able

to be treated with short acting beta$_2$-agonist (SABA) alone for the duration of the study (up until the final follow-up visit).

- A sufficient number of subjects will be enrolled to ensure that 20 per treatment arm complete the 8 week study treatment period and the BAC at the one week follow-up visit (one week after the 8th dose of study treatment).

If subjects prematurely discontinue the study, additional replacement subjects may be recruited and assigned the next available randomisation number, at the discretion of the Sponsor, in consultation with the investigator.

Following the interim analysis, additional subjects may also be enrolled to counteract the impact of individuals not exhibiting an LAR response on placebo.

Accounting for replacements and interim analysis recommendations, the total number of subjects to be randomised in this study is not expected to exceed 60.

4.4. Design Justification

- GSK2245035 is currently being developed for patients with allergic asthma. It has been studied in healthy volunteers (HVT) and subjects with allergic rhinitis with and without mild asthma.
- This study will assess the safety, target engagement, and efficacy of GSK2245035 in subjects with allergic asthma using a placebo-controlled, parallel group design and a BAC model. The BAC model is an established methodology and will be used in this study to determine whether 8 weekly doses of 20 ng i.n. GSK2245035 attenuates allergen-induced asthmatic responses compared to placebo. In addition to the assessment after BAC at the end of the 8-week treatment (1 week after the 8th dose), additional BACs will be performed at approximately 4 weeks and 12 weeks after the 8th dose to assess the durability of the effect.
- In an 8-week repeat dose safety study TL7116958 [GlaxoSmithKline Document Number 2013N174200_00], there was a reduction of TNSS at15 minutes post-NAC. This could indicate an effect of GSK2245035 treatment on mast cell activation and mediator release. In the current study, nasal lining fluid will be collected before and up to 5 minutes after nasal allergen challenges to evaluate the impact of GSK2245035 on mast cell mediator release. NACs will be performed approximately 24 hours after the BACs that are conducted at screening and at approximately 1 week and 4 weeks after the final dose.
- Interim analysis: Previous experience within GSK suggests some individuals (approximately 20%) fail to demonstrate a LAR response after the screening visit (i.e. they pass the screening criteria but do not demonstrate another LAR response when receiving placebo during the on-treatment phase). Those GSK studies were mainly crossover designs with a placebo in every sequence, so subjects who did not demonstrate a post screening LAR could be retrospectively identified and excluded from the statistical analyses. However this study has parallel groups and the profiles of a “perfect” drug effect and an inconsistent LAR subject are indistinguishable so retrospective exclusions cannot be made for the GSK2245035 arm. If the rate of “non-reproducible LAR” subjects is large it will reduce the probability of success.

The purpose of the interim analysis is to evaluate whether the non-reproducible LAR rate is reducing the expected probability of success by more than 10% and, if it is, to perform an unblinded review of the observed data to assess the impact and cease further recruitment into the study, or enrol additional subjects to counteract the effects. Simulations were used to quantify the expected reduction in probability of success, using an estimate of the non-responder rate from the placebo arm only. The timing of the interim is linked to the enrolment rates and will be triggered by whichever occurs first i) Approximately 1 year after the first subject successfully completes the Follow Up 1 assessments or, ii) When 28 subjects have successfully completed the Follow Up 1 assessments.

- It is hypothesized that TLR7-mediated immunomodulation requires allergen to be present and exposure to aeroallergens may fluctuate or remain relatively constant depending on the allergen, time of year, and a subject's household characteristics or activities. Further, exposure to certain aeroallergens may be presumed and, in other instances, is unknown unless outdoor air and household samples are analyzed. For example, allergen exposure may be presumed if a subject who is sensitized to and challenged with grass pollen enrolls during grass pollen season or whose treatment is expected to coincide with grass pollen season. A subject sensitized to animal epithelia who has that animal in his/her house or has occupational exposure may be presumed to have exposure. These subjects will potentially exhibit a different response to study drug to someone without similar exposure to the allergen. Exposure to certain allergens, particularly perennial allergens (i.e. house dust mite) cannot be presumed and will be considered unknown for the purposes of this protocol. Therefore, the allergen a subject is administered for the BAC will need to be considered according to whether the subject has exposure to that allergen outside of the challenge. Consequently, the randomization will be stratified by ‘presumed’ and ‘unknown’ exposure to allergen in addition to stratification by centre.

4.5. Dose Justification

The 20ng weekly i.n. dose of GSK2245035 was selected based on previous single and repeat dose studies in healthy volunteers (HVTs) and allergic rhinitis subjects with and without mild asthma.

- In the first time in human study TL7114450 (dose range 2 ng – 100 ng) [GlaxoSmithKline Document Number 2011N120740_00], single i.n. administration of doses up to 40 ng GSK2245035 was well tolerated in HVT and subjects with symptomatic seasonal allergic rhinitis.
- In the 4-week repeat dose study TL7116392 [GlaxoSmithKline Document Number 2011N126275_00], the repeat dose of i.n. GSK2245035 40 and 80 ng had an acceptable safety profile. Adverse events (AEs) likely related to TLR7-induced cytokines, such as headache, fever, chills/rigors and nausea were observed in one subject receiving placebo, 4 of 6 subjects receiving 40 ng GSK2245035 and one of 7 subjects receiving 80 ng GSK2245035.
- In a 8-week repeat dose safety study TL7116958 [GlaxoSmithKline Document Number 2013N174200_00] with i.n. GSK2245035 in subjects with symptomatic allergic rhinitis and mild asthma, 20 ng and 80 ng weekly doses produced sustained

reductions in nasal allergic reactivity based on the total nasal symptom score after nasal allergen challenge up to 3 weeks post treatment. The tolerability of 20 ng GSK2245035 was similar to placebo with no reports of cytokine-related fever. Cytokine release syndrome (CRS)-related symptoms were self-resolving and occurred infrequently over the 8-week study period in subjects receiving 20 ng GSK2245035. GSK2245035 80 ng was poorly tolerated. All but one subject receiving 80 ng experienced flu-like symptoms. Although CRS-related symptoms were also self-resolving at the 80 ng dose, subjects experienced them fairly consistently over the study period. TLR7-induced biomarkers indicated target engagement at both doses, with evidence for sustained activation of TLR7-mediated pathway intranasally up to 3 weeks post treatment only at the 80ng dose. Reviewing the totality of data obtained in these previous studies supports further study of a repeated weekly dose of 20 ng i.n. GSK2245035.

4.6. Benefit:Risk Assessment

Summaries of findings from both clinical and non-clinical studies conducted with GSK2245035 can be found in the Investigator’s Brochure [GlaxoSmithKline Document Number 2010N110564_04]. The following section outlines the risk assessment and mitigation strategy for this protocol:

4.6.1. Risk Assessment

Potential Risk of Clinical Significance	Summary of Data/Rationale for Risk	Mitigation Strategy
**Investigational Product (IP) [GSK2245035]**		
Events potentially related to cytokine induction.	The principal systemic effect seen with GSK2245035 in intranasal studies in animals (mice, rats, rabbits, and monkeys) up to 8 weeks in duration with weekly dosing was increases in the levels of specific cytokines; all other findings were considered to be secondary to this primary response (i.e., nasal irritancy, increased body temperature, increased heart rate, alterations in splenic and lymph node cellularity and minor changes in some clinical chemistry parameters). With the exception of the splenic and lymph node effects, all the observed changes would be readily monitorable in humans. The effects that were seen in the spleen and lymph node were of low severity and were shown to be fully reversible on withdrawal from treatment in the monkey. The observed cytokine response is consistent with the known TLR7 pharmacology of GSK2245035. Although a no effect level for cytokine response was not determined in the monkey, a No Observed Adverse Event Level (NOAEL) of 35.9 ng/kg/day was determined for the only secondary finding	The study will be undertaken at experienced research units. The sites have all necessary facilities to handle medical emergencies. All subjects will receive each dose in the unit and will be monitored closely after each dose (for approximately 1-2 hours). Each subject will receive a phone call approximately 12 hours following administration of at least the first two doses to assess tolerability. They will be provided with a thermometer to measure body temperature. Thorough monitoring of any AEs will be performed throughout the treatment period to allow early assessment and mitigation of risk.<br><br>Only subjects in good general health with a diagnosis of mild asthma will participate in this study (see Inclusion/Exclusion Criteria, Section 5.1 and Section 5.2). Subjects with history of severe asthma are excluded from the study.

Potential Risk of Clinical Significance	Summary of Data/Rationale for Risk	Mitigation Strategy
	considered to be adverse, nasal irritancy [GlaxoSmithKline Document Number 2010N110564_04].<br><br>GSK2245035 has been previously tested in subjects with allergic rhinitis with or without mild asthma at the same dose (20 ng) and the same i.n. regimen (8 weekly administrations) proposed for this study.<br><br>I.n. GSK2245035 induced transient dose related increases in body temperature (GSK2245035 80 ng maximum 1.37ºC; GSK2245035 20 ng group maximum 0.34ºC).<br><br>I.n. GSK2245035 induced dose related increases in the frequency and severity of AEs possibly related to cytokine induction. Headache was the most frequently reported cytokine related AE for all treatments (79% subjects in GSK2245035 80 ng, 29% subjects in GSK2245035 20 ng and 21% subjects in placebo group), including mild, moderate and severe reports.<br><br>Fever was a frequently reported cytokine related AE at GSK2245035 80 ng (58%) while no reports of cytokine related fever were noted at GSK2245035 20ng or	

Potential Risk of Clinical Significance	Summary of Data/Rationale for Risk	Mitigation Strategy
	placebo. Cytokine related fever mainly emerged after the second dosing visit; most cytokine related fever occurred within 12 hours of dosing.<br><br>One subject receiving GSK2245035 80ng was withdrawn due to severe myalgia occurring after the second dose. One GSK2245035 20ng subject reported myalgia and arthralgia of moderate severity.<br><br>At both doses cytokine related AEs were self resolving with a median duration of approximately ≤ 1 day.<br><br>No severe cytokine related events were reported at GSK2245035 20ng.<br><br>There were no reports of vomiting, diarrhea or hypotension in any treatment group.	
Nasal irritancy	Nasal irritancy was noted in rats and monkeys after repeat intranasal dosing; the effects in the rat were seen only at very high dose levels/concentrations (Section 4.5 of the Investigator Brochure (IB)) [GlaxoSmithKline Document Number 2010N110564_04].<br><br>The finding from the rat and monkey studies	Thorough monitoring of nasal AEs will be performed throughout the treatment period to allow early mitigation of risk. Nasal examinations will be performed pre-dose and 24 hours post-dose on dosing visits 1 and 8.

Potential Risk of Clinical Significance	Summary of Data/Rationale for Risk	Mitigation Strategy
	is considered to be clinically relevant, but it is unlikely to occur in humans at the proposed clinical doses; should unexpected irritancy be seen, it would be readily detectable and has been shown to be readily reversible on withdrawal from treatment in the monkey. The no observed adverse effect level (NOAEL) in the monkey for nasal irritancy (35.9 ng/kg/day) provides adequate cover for the proposed 20 ng weekly dose in this study. Therefore, GSK2245035 displays an acceptable safety profile for an intranasal medicine.<br><br>Nasal irritancy has been assessed in clinical studies up to 8 weeks' duration. I.n. GSK2245035 is well-tolerated locally as determined by nasal examinations, adverse events and nasal symptom scores.	
**Study Procedures**		
Anaphylaxis, including bronchospasm occurring after bronchial, intranasal, or intradermal administration of allergen.	Subjects will inhale (orally and intranasally) various concentrations of allergen to which they are sensitized. Bronchial allergen challenges will be performed at screening, and at the follow-up visits (1, 4 and 12 weeks post final dose).<br><br>Intranasal allergen and intradermal testing	Only sites that have a significant experience with administering bronchial, intranasal, and intradermal allergen challenges and in the management of anaphylaxis and acute bronchospasm will be used in this study. Careful monitoring of all symptoms will take place to assure safety. The responsible

Potential Risk of Clinical Significance	Summary of Data/Rationale for Risk	Mitigation Strategy
	will be conducted approximately 24 hours after the bronchial allergen challenges performed at screening and at the 1 and 4 week follow-up visits.<br><br>Allergen testing procedures may induce anaphylaxis, including acute bronchospasm.	physician will be appropriately trained to treat anaphylaxis and acute bronchospasm, including appropriate use of resuscitation equipment, which must be close enough to respond quickly to an emergency.<br><br>Subjects will be continually monitored at the study centre for at least 10 hours after each bronchial allergen challenge and 6 hours after intranasal and intradermal challenges. The procedure should be stopped immediately if signs/symptoms of anaphylaxis and/or acute bronchospam develop and rescue medication treatments will be readily available.<br><br>Subjects will be allowed to leave the unit only after the investigator has reviewed and assessed the available safety data.<br><br>Subjects will receive a Peak Expiratory Flow (PEF) meter to measure PEF at home from the start of dosing through to completion of the 4 week follow-up visit assessments. The subject will be instructed to contact the investigator if PEF measurements are outside the normal range for the subject. Subjects will also have their PEF levels checked in the unit after

Potential Risk of Clinical Significance	Summary of Data/Rationale for Risk	Mitigation Strategy
		administration of the final bronchial allergen challenge at the 12 week follow-up visit.
Sputum induction	Bronchoconstriction may occur following inhalation of saline.	In the event of bronchoconstriction, the procedure will be stopped immediately and the subject treated with a short-acting inhaled bronchodilator.

4.6.2. Benefit Assessment

Benefit considerations may include:

Potential benefit of receiving study drug for the study duration that may reduce allergic reactivity.

Contributing to the process of developing possible disease-modifying therapies for asthma.

Medical evaluations/assessments associated with study procedures [e.g. a physical examination, Electrocardiogram (ECG), vital signs, clinical laboratory assessments, to screen any potential health issues].

4.6.3. Overall Benefit:Risk Conclusion

Measures taken to minimise and manage potential risk to subjects participating in this study, coupled with the potential benefits justify the conduct of a Phase IIa study with i.n. GSK2245035 in mild asthmatics.

5. SELECTION OF STUDY POPULATION AND WITHDRAWAL CRITERIA

Specific information regarding warnings, precautions, contraindications, adverse events, and other pertinent information on the GSK investigational product or other study treatment that may impact subject eligibility is provided in the IB [GlaxoSmithKline Document Number 2010N110564_04].

Deviations from inclusion and exclusion criteria are not allowed because they can potentially jeopardize the scientific integrity of the study, regulatory acceptability or subject safety. Therefore, adherence to the criteria as specified in the protocol is essential.

5.1. Inclusion Criteria

A subject will be eligible for inclusion in this study only if all of the following criteria apply:

AGE
• Between 18 and 65 years of age inclusive, at the time of signing the informed consent.

TYPE OF SUBJECT AND DIAGNOSIS INCLUDING DISEASE SEVERITY
• Diagnosis of asthma, as defined by the history of respiratory symptoms such as wheeze, shortness of breath, chest tightness and cough that vary over time and in intensity, together with variable expiratory airflow limitation [GINA guideline, 2015] for at least 6 months prior to screening.<br><br>• Current asthma therapy. Intermittent short-acting beta-agonist (SABA) alone (on

average for no more than 2 days per week)

- Positive skin prick test (3mm or more greater than negative control) to common perennial or seasonal aeroallergen(s) (i.e., house dust mite, cat dander, grass pollen) at screening.

- Pre-bronchodilator $FEV_1 > 70$ % predicted normal at Screening Visit 1

NOTE: Predicted values will be based upon Quanjer, 2012.

- EAR with ≥ 20 % FEV_1 decrease between 5 and 30 minutes after the final allergen concentration is obtained at the screening BAC (decreases relative to the saline).

- LAR with three FEV_1 decreases of ≥ 15 % between 4 and 10 hours after the final allergen concentration is obtained at the screening bronchial allergen challenge, with two FEV_1 decreases being at consecutive time points (decreases relative to the saline).

- Subjects who are current non-smokers (defined as no use of any tobacco products in the 6-month period preceding the screening visit) and have a pack history of < 10 pack years

Number of pack years = (number of cigarettes per day/20) x number of years smoked

WEIGHT
• Bodyweight ≥ 45kg

SEX

- Male OR female of non-reproductive potential

Males:

Male subjects with female partners of child bearing potential must comply with one of the following contraception requirements from the time of first dose of study medication until the final follow-up visit.

a. Vasectomy with documentation of azoospermia.

b. Male condom plus partner use of one of the contraceptive options below:

   - Contraceptive subdermal implant
   - Intrauterine device or intrauterine system
   - Oral contraceptive, either combined or progestogen alone [Hatcher, 2007a] Injectable progestogen [Hatcher, 2007a]
   - Contraceptive vaginal ring [Hatcher, 2007a]
   - Percutaneous contraceptive patches [Hatcher, 2007a]

This is an all-inclusive list of those methods that meet the following GSK definition of highly effective: having a failure rate of less than 1% per year when used consistently and correctly and, when applicable, in accordance with the product label. For non-product methods (e.g., male sterility), the investigator determines what is consistent and correct use. The GSK definition is based on the definition provided by the International Conference on Harmonization of Technical Requirements for Registration of Pharmaceuticals for Human Use (ICH) [ICH M3 (R2) 2009]."

The investigator is responsible for ensuring that subjects understand how to properly use these methods of contraception.

Females:

A female subject is eligible to participate if she is not pregnant (as confirmed by a negative [serum or urine] human chorionic gonadotrophin (hCG) test), not lactating, and where the following condition applies:

Non-reproductive potential defined as:

- Pre-menopausal females with one of the following:
  - Documented tubal ligation
  - Documented hysteroscopic tubal occlusion procedure with follow-up confirmation of bilateral tubal occlusion
  - Hysterectomy
  - Documented Bilateral Oophorectomy
- Postmenopausal defined as 12 months of spontaneous amenorrhea [in questionable cases a blood sample with simultaneous follicle stimulating hormone (FSH) and estradiol levels consistent with menopause (refer to laboratory reference ranges for confirmatory levels)]. Females on hormone replacement therapy (HRT) and whose menopausal status is in doubt will be required to use one of the highly effective contraception methods if they wish to continue their HRT during the study. Otherwise, they must discontinue HRT to allow confirmation of post-menopausal status prior to study enrolment..

INFORMED CONSENT
• Capable of giving signed informed consent which includes compliance with the requirements and restrictions listed in the consent form and in this protocol.

5.2. Exclusion Criteria

A subject will not be eligible for inclusion in this study if any of the following criteria apply:

CONCURRENT CONDITIONS/MEDICAL HISTORY (INCLUDES LIVER FUNCTION AND QTc INTERVAL)

1. Alanine transaminase (ALT) >2xUpper Limit of Normal (ULN) and bilirubin >1.5xULN (isolated bilirubin >1.5xULN is acceptable if bilirubin is fractionated and direct bilirubin <35%).
2. Current or chronic history of liver disease, or known hepatic or biliary abnormalities (with the exception of Gilbert's syndrome or asymptomatic gallstones)
3. Heart rate corrected QT interval (QTc) > 450 msec or QTc > 480 msec in subjects with Bundle Branch Block

   NOTES:

   - The QTc is the QT interval corrected for heart rate according to Bazett's formula (QTcB), Fridericia's formula (QTcF), or another method, machine-read or manually over-read.
   - The specific formula that will be used to determine eligibility and discontinuation for an individual subject should be determined prior to initiation of the study. In other words, several different formulae cannot be used to calculate the QTc for an individual subject and then the lowest QTc value used to include or discontinue the subject from the trial.
   - For purposes of data analysis, QTcB, QTcF, another QT correction formula, or a composite of available values of QTc will be used as specified in the Reporting and Analysis Plan (RAP).

4. Asthma exacerbation requiring treatment with oral corticosteroids or hospitalization within 3 months prior to screening
5. History of life-threatening asthma, defined as an asthma episode that required intubation and/or was associated with hypercapnea, respiratory arrest or hypoxic seizures within the last 10 years.
6. Evidence of concurrent respiratory diseases such as pneumonia, pneumothroax, atelectasis, pulmonary fibrotic disease, allergic bronchopulmonary aspergillosis, cystic fibrosis, bronchopulmonary dysplasia, *or* other respiratory abnormalities other than asthma.
7. Other concurrent diseases/abnormalities: A subject must not have any clinically significant, uncontrolled condition or disease state that, in the opinion of the investigator, would put the safety of the subject at risk through study participation or would confound the interpretation of the efficacy results if the condition/disease exacerbated during the study.
8. Respiratory tract infection that is not resolved within 2 weeks prior to screening.

CONCOMITANT MEDICATIONS
9. Treatment with intranasal steroid, inhaled corticosteroid (ICS) with or without long-acting beta2-agonist (LABA), and treatment with non-ICS controller asthma medications (i.e., leukotriene modifier, methylxanthines) within 4 weeks prior to screening.<br>10. Use of long-acting antihistamines within 7 days' or short-acting antihistamines within 72 hours prior to the screening skin prick test.<br>11. Treatment with systemic corticosteroids within 6 weeks prior to screening.<br>12. Use of inhaled SABAs as rescue treatment on average for more than 2 days per week.

RELEVANT HABITS
13. History of regular alcohol consumption within 6 months of the study defined as:<br>An average weekly intake **of >14 units for males and females**. One unit is equivalent to 8 g of alcohol: a half-pint (~240 ml) of beer, 1 glass (125 ml) of wine or 1 (25 ml) measure of spirits.

CONTRAINDICATIONS
14. Patient known to be intolerant to salbutamol or albuterol<br>15. History of sensitivity to any of the study medications, or components thereof or a history of drug or other allergy that, in the opinion of the investigator or GSK Medical Monitor, contraindicates their participation.

DIAGNOSTIC ASSESSMENTS AND OTHER CRITERIA
16. Presence of hepatitis B surface antigen (HBsAg), positive hepatitis C antibody test result at screening or within 3 months prior to first dose of study treatment. For potent immunosuppressive agents, subjects with presence of hepatitis B core antibody (HBcAb) should also be excluded.<br>17. A positive pre-study drug/alcohol screen<br>18. A positive test for Human Immunodeficiency Virus (HIV) antibody<br>19. Where participation in the study would result in donation of blood or blood products in excess of 500 mL within a 56 day period.<br>20. The subject has participated in a clinical trial and has received an investigational product within the following time period prior to the first dosing day in the current study: 30 days, 5 half-lives or twice the duration of the biological effect of the investigational product (whichever is longer)<br>21. Exposure to more than 4 investigational medicinal products within 12 months prior to the first dosing day

5.3. Screening Failures/Re-screening

Screen failures are defined as subjects who consent to participate in the clinical trial but are never subsequently randomized. In order to ensure transparent reporting of screen failure subjects, meet the Consolidated Standards of Reporting Trials (CONSORT) publishing requirements, and respond to queries from Regulatory authorities, a minimal set of screen failure information is required including Demography, Screen Failure details, Eligibility Criteria, and Serious Adverse Events (see Section 7.4.1.6).

Re-screening will not be permitted in this study. Sites are encouraged to screen subjects who have previously undergone BAC and have demonstrated a LAR.

5.4. Withdrawal/Stopping Criteria

Reasons for withdrawal from the study may include:

Use of a prohibited medication that cannot be safely discontinued while maintaining the subject’s health.

Treatment code is unblinded.

Pregnancy: Any female subject who becomes pregnant.

The following actions must be taken in relation to a subject who fails to attend the clinic for a required study visit:

- The site must attempt to contact the subject and re-schedule the missed visit as soon as possible.
- The site must counsel the subject on the importance of maintaining the assigned visit schedule and ascertain whether or not the subject wishes to and/or should continue in the study.
- In cases where the subject is deemed ‘lost to follow up’, the investigator or designee must make every effort to regain contact with the subject (where possible, 3 telephone calls and if necessary a certified letter to the subject’s last known mailing address or local equivalent methods). These contact attempts should be documented in the subject’s medical record.
- Should the subject continue to be unreachable, only then will he/she be considered to have withdrawn from the study with a primary reason of “Lost to Follow-up”.

A subject may withdraw from study treatment at any time at his/her own request, or may be withdrawn at any time at the discretion of the investigator for safety, behavioural or administrative reasons. If a subject withdraws from the study, he/she may request destruction of any samples taken, and the investigator must document this in the site study records.

Subjects withdrawn from the study while already exposed to IP will be asked to complete all safety assessments as outlined in the Time and Events Table (Section 7.1.2). Relevant pages of the case report form (CRF) should be completed by the investigator.

The replacement of withdrawn subjects will be at the discretion of the sponsor. If a subject misses one treatment dose, the investigator will consult with the GSK medical monitor as to whether the subject can receive further treatment. If more than one treatment dose is missed, then the subject may be withdrawn from the study, at the discretion of the Sponsor, in consultation with the investigator.

If subjects prematurely discontinue the study, additional replacement subjects may be recruited and assigned the next available randomisation number, at the discretion of the Sponsor, in consultation with the investigator.

5.4.1. Liver Chemistry Stopping Criteria

Liver chemistry stopping and increased monitoring criteria have been designed to assure subject safety and evaluate liver event etiology (in alignment with the Food and Drug Administration (FDA) premarketing clinical liver safety guidance). http://www.fda.gov/downloads/Drugs/GuidanceComplianceRegulatoryInformation/Guidances/UCM174090.pdf

Liver Safety Required Actions and Follow up Assessments Section can be found in Appendix 2.

5.4.1.1. Study Treatment Restart or Rechallenge

Study treatment restart or rechallenge after liver chemistry stopping criteria are met by any subject participating in this study is not allowed.

5.4.2. QTc Stopping Criteria

The *same* QT correction formula *must* be used for *each individual subject* to determine eligibility for and discontinuation from the study. This formula may not be changed or substituted once the subject has been enrolled.

- For example, if a subject is eligible for the protocol based on QTcB, then QTcB must be used for discontinuation of this individual subject as well.
- Once the QT correction formula has been chosen for a subject's eligibility, the *same formula* must continue to be used for that subject *for all QTc data being collected for data analysis*. Safety ECGs and other non-protocol specified ECGs are an exception.

The QTc should be based on single or averaged QTc values of triplicate electrocardiograms obtained over a brief (e.g., 5-10 minute) recording period.

A subject who meets either of the bulleted criteria below will be withdrawn from the study:

- QTc > 500 msec OR Uncorrected QT > 600 msec
- Change from baseline of QTc > 60 msec

For subjects with underlying **bundle branch block**, follow the discontinuation criteria listed below:

Baseline QTc with Bundle Branch Block	Discontinuation QTc with Bundle Branch Block
< 450 msec	> 500 msec
450 – 480 msec	≥ 530 msec

5.5. Subject and Study Completion

A completed subject is one who has completed all phases of the study including all of the follow-up visits.

The date of the end of the study is defined as the date of the last subject's last follow-up visit.

6. STUDY TREATMENT

6.1. Investigational Product and Other Study Treatment

The term 'study treatment' is used throughout the protocol to describe any combination of products received by the subject as per the protocol design. Study treatment may therefore refer to the individual study treatments or the combination of those study treatments.

	Study Treatment	
**Product name:**	GSK2245035 Nasal Spray Solution 0.2 mcg/mL	Placebo
**Formulation description:**	A solution formulation in saline preserved with Benzalkonium Chloride and Disodium Edetate	As for GSK2245035 nasal spray solution except for omission of the active ingredient
**Dosage form:**	Nasal spray solution	Nasal spray solution
**Unit dose strength(s)/Dosage level(s):**	10 ng per actuation	Not applicable
**Route of Administration:**	Intranasal<br>Once weekly	Intranasal<br>Once weekly
**Dosing instructions:**	1 spray per nostril	1 spray per nostril
**Physical description:**	An amber glass bottle fitted with a screw fit atomizing pump	Visually matched to the active
**Device:**	Type 1 amber glass bottle fitted with a metered Valois VP7 pump	Type 1 amber glass bottle fitted with a metered Valois VP7 pump
**Manufacturer/ source of procurement:**	GSK	GSK
**Method for individualizing dosage:**	N/A	N/A

6.2. Treatment Assignment

Subjects will be assigned to one of two treatments (either 20 ng GSK2245035 or placebo), in accordance with the randomization schedule generated by Clinical Statistics, prior to the start of the study, using validated internal GSK software RandAll NG. The randomisation will be stratified by centre and also by exposure state during the treatment phase to the allergen selected for BAC (two strata – "Presumed" and "Unknown"). See Section 4.4 for further details regarding allergen exposure.

It is planned that an Interactive Response Technology (IRT) will be used in this study to assign randomization and drug containers. It can also be used to emergency unblind subjects if required (see Section 6.3).

Once assigned to a subject a randomization number may not be re-used.

More details of how and when a subject is allocated a randomization number and the subject numbering convention will be given in the Study Reference Manual (SRM).

6.3. Blinding

This will be a double-blind (sponsor open), study (investigators and subjects are blinded, and sponsor will be unblinded).

During the study, there will be an unblinded interim analysis. Some key GSK study team members may be unblinded as part of this analysis and review. Initially, only the study statistician(s) (and supporting programmers), project physician lead, and medical monitor will be unblinded, and if further data reviews are required then details of who were un-blinded to what data and when will be included in the clinical study report.

On-going safety reviews will be conducted during the course of the study in an unblinded manner. Key study team members may be unblinded as part of this review. An external consultant, who is not a GSK employee, may also be unblinded to enable independent review of emerging safety data.

If the GSK medical monitor deems it necessary to unblind data in order to enhance the evaluation of safety and tolerability this will be permitted but also documented and discussed in the clinical study report. The GSK medical monitor will also decide whether to un-blind the investigator(s)/site(s) at that time, or just the sponsor study staff. Details of who were un-blinded to what data and when would then be included in the clinical study report.

Unblinding would occur using the IRT system.

The following will apply.

The investigator or treating physician may unblind a subject's treatment assignment only in the case of an emergency OR in the event of a serious medical condition when knowledge of the study treatment is essential for the appropriate clinical management or welfare of the subject as judged by the investigator.

Investigators have direct access to the subject's individual study treatment.

It is preferred (but not required) that the investigator first contacts the Medical Monitor or appropriate GSK study personnel to discuss options before unblinding the subject's treatment assignment.

If GSK personnel are not contacted before the unblinding, the investigator must notify GSK as soon as possible after unblinding, but without revealing the treatment assignment of the unblinded subject, unless that information is important for the safety of subjects currently in the study.

The date and reason for the unblinding must be fully documented in the CRF.

A subject will be withdrawn if the subject's treatment code is unblinded by the investigator or treating physician. The primary reason for discontinuation (the event or condition which led to the unblinding) will be recorded in the CRF.

GSK's Global Clinical Safety and Pharmacovigilance (GCSP) staff may unblind the treatment assignment for any subject with a Serious Adverse Event (SAE). If the SAE requires that an expedited regulatory report be sent to one or more regulatory agencies, a copy of the report, identifying the subject's treatment assignment, may be sent to investigators in accordance with local regulations and/or GSK policy.

6.4. Packaging and Labeling

The contents of the label will be in accordance with all applicable regulatory requirements.

6.5. Preparation/Handling/Storage/Accountability

No special preparation of study treatment is required.

One device is dispensed at dosing visit 1 (to use for the first 4 dose administrations), and a second device will be dispensed at dosing visit 5 (to use for administration of the fifth to eighth doses). Both of these devices will remain in the clinic. All eight doses will be administered to the subjects in the clinic.

The investigator or designee must confirm appropriate temperature conditions have been maintained during transit for all study treatment received and any discrepancies are reported and resolved before use of the study treatment.

Only subjects enrolled in the study may receive study treatment and only authorized site staff may supply or administer study treatment. All study treatments must be stored in a secure environmentally controlled and monitored (manual or automated) area in accordance with the labelled storage conditions with access limited to the investigator and authorized site staff.

The investigator, institution, or the head of the medical institution (where applicable) is responsible for study treatment accountability, reconciliation, and record maintenance (i.e. receipt, reconciliation and final disposition records).

Further guidance and information for final disposition of unused study treatment are provided in the SRM.

Under normal conditions of handling and administration, study treatment is not expected to pose significant safety risks to site staff. Take adequate precautions to avoid direct eye or skin contact and the generation of aerosols or mists. In the case of unintentional occupational exposure notify the monitor, Medical Monitor and/or GSK study contact.

A Material Safety Data Sheet (MSDS)/equivalent document describing occupational hazards and recommended handling precautions either will be provided to the investigator, where this is required by local laws, or is available upon request from GSK.

6.6. Compliance with Study Treatment Administration

When subjects are dosed at the site, they will receive study treatment directly from the investigator or designee, under medical supervision. The date and time of each dose administered in the clinic will be recorded in the source documents. The dose of study treatment and study subject identification will be confirmed at the time of dosing by a member of the study site staff other than the person administering the study treatment.

6.7. Treatment of Study Treatment Overdose

For this study, any dose of GSK2245035 over the stated maximum of the study treatment will be considered an overdose.

GSK does not recommend specific treatment for an overdose. The investigator will use clinical judgment to treat any overdose and provide supportive care as appropriate. The investigator will inform the GSK Medical Monitor immediately, and will document the quantity of the excess dose as well as the duration of the overdosing in the CRF.

Decisions regarding dose interruptions or modifications will be made by the investigator in consultation with the Medical Monitor based on the clinical evaluation of the subject.

6.8. Treatment after the End of the Study

Subjects will not receive any additional treatment from GSK after completion of the study because the indication being studied is not life-threatening or seriously debilitating and other treatment options are available.

The investigator is responsible for ensuring that consideration has been given to the post-study care of the subject's medical condition, whether or not GSK is providing specific post-study treatment.

6.9. Challenge Agents

6.9.1. Bronchial, intranasal and intradermal allergen challenge/administration

Appropriate allergen extracts will be purchased by the clinical unit(s) or provided.

The specific allergen extract that will be used for the BAC, NAC and ID challenge will be selected on the basis of each subject's skin prick test. The same allergen will be used for each method of testing. The NAC and ID challenge are not required for eligibility.

A detailed description of how all of the allergen extracts will be selected, the preparation of the allergen extracts and details of the bronchial, intranasal and intradermal allergen challenge techniques can be found in the SRM.

6.10. Lifestyle and/or Dietary Restrictions

6.10.1. Meals and Dietary Restrictions

Dietary restrictions as outlined below will be observed during the time periods specified prior to entry to the clinic and dosing.

6.10.2. Caffeine, Alcohol, and Tobacco

Subjects will abstain from ingesting excessive amounts (more than one cup) of caffeine- or xanthine-containing products (e.g. coffee, tea, cola drinks, chocolate) for 6 hours prior to each dosing.

During each visit in the clinic, subjects will abstain from alcohol for 24 hours prior to entry to the clinic until collection of all assessment is completed.

Subjects will be reminded that all forms of smoking should be avoided during the course of the study.

6.10.3. Activity

Subjects will abstain from strenuous exercise for 48 hours prior to each blood collection for clinical laboratory tests.

Subjects should refrain from all recreational drugs from screening until the final follow-up visit.

6.11. Concomitant Medications and Non-Drug Therapies

6.11.1. Permitted Medications and Non-Drug Therapies

Any concomitant medications not specifically permitted will be considered on a case by case basis by the investigator and GSK medical monitor.

Subjects are permitted to take inhaled short-acting β2-agonists (SABA) as rescue treatment, but must withhold them for at least 6 hours prior to any study visits with lung function assessment or BAC (screening and follow-up visits 1-3).

Medication to control hypertension is permitted, providing it is anticipated to remain stable from screening to follow up. Beta blockers are not permitted.

Short term use of anti-histamines is permitted with some limitations as outlined in Section 6.11.2; the use of paracetamol and NSAIDS for short-term pain relief is also permitted.

6.11.2. Prohibited Medications and Non-Drug Therapies

Subjects may not receive the following treatments from 4 weeks prior to screening until the final follow-up visit:

Intranasal steroids

Inhaled corticosteroid (ICS) with or without a long acting beta-2-agonist (LABA)

Non-ICS controller asthma medications (i.e. leukotriene modifier, methylxanthines).

Use of long-acting antihistamines within 7 days' or short-acting antihistamines within 72 hours prior to BAC, NAC or ID challenge is prohibited.

Subjects are prohibited from taking systemic corticosteroids within 6 weeks prior to screening.

Subjects must abstain from taking prescription or non-prescription drugs (including vitamins and dietary or herbal supplements) except as described in Section 6.11.1 within 7 days prior to the first dose of study medication until completion of the follow-up visits, unless in the opinion of the investigator and sponsor the medication will not interfere with the study.

7. STUDY ASSESSMENTS AND PROCEDURES

Protocol waivers or exemptions are not allowed with the exception of immediate safety concerns. Therefore, adherence to the study design requirements, including those specified in the Time and Events Table, are essential and required for study conduct.

This section lists the procedures and parameters of each planned study assessment. The exact timing of each assessment is listed in the Time and Events Table Section 7.1

The following points must be noted:

- If assessments are scheduled for the same nominal time, THEN the assessments should occur in the following order:

    1. 12-lead ECG
    2. vital signs
    3. blood draws.

    Note: The timing of the assessments should allow the blood draw to occur at the exact nominal time.

- The timing and number of planned study assessments, including safety, pharmacokinetic, pharmacodynamic/biomarker assessments may be altered during the course of the study based on newly available data to ensure appropriate monitoring. Similarly the timing of the post-treatment allergen challenges may change based on new information acquired during the course of the study.

- Details of the order of planned assessments for each visit and the time windows around these visits and assessments will be given in the SRM.

- The change in timing or addition of time points for any planned study assessments must be documented in a Note to File which is approved by the relevant GSK study team member and then archived in the study sponsor and site study files, but this will not constitute a protocol amendment.

- The Institutional Review Board (IRB)/Independent Ethics Committee (IEC) will be informed of any safety issues that require alteration of the safety monitoring scheme or amendment of the Informed Consent Form.

- No more than 500 mL of blood will be collected over the duration of the study, including any extra assessments that may be required.

7.1. Time and Events Tables

7.1.1. Time and Events Table – Screening Visit(s)

Procedure	Screening Visit 1 (Day -28 to Day -22)	Screening Visit 2 (Day -21 to Day -3)	24 hours after Screening Visit 2 (Day -20 to Day -2)	Notes
Informed consent signing	X			Includes genetics consent
Inclusion and exclusion criteria/eligibility review	X	X		
Demographics	X			
Medical history (including substance usage and family history of premature CV disease)	X			
Past and current medical conditions including cardiovascular medical history	X			
Concomitant medication review	X			
Full physical exam including height and weight	X			
Urine drug/alcohol screen	X			
Urine or serum pregnancy test (all females)	X			No women of childbearing potential are being enrolled. However this test is needed to confirm pregnancy status at screening.
Serum FSH and estradiol for post-menopausal females	X			
HIV, Hep B and Hep C screen	X			
Laboratory assessments (including liver chemistries, haematology, clinical chemistry and urinalysis)	X			
Screening Spirometry assessment	X			To confirm FEV_1 > 70% predicted
SAE Collection	X	X	X	
12-lead ECG	X			
Vital signs	X			
Skin prick test	X			To confirm eligibility
FeNO measurement	X		X	Collected prior to sputum induction
Saline and incremental BAC challenge plus associated spirometry measurements		X		Including pre-procedure FEV_1 assessment and repeat FEV_1 measurements up to 10 hours post BAC
Sputum induction and processing for BAC associated sample collection	X		X	Sputum inductions must be separated by at least 3 days

Procedure	Screening Visit 1 (Day -28 to Day -22)	Screening Visit 2 (Day -21 to Day -3)	24 hours after Screening Visit 2 (Day -20 to Day - 2)	Notes
Laboratory assessments (haematology only)		X	X	Blood samples should be taken pre-BAC on SV2 and on the following day at 24hrs post BAC
Blood samples for PBMC preparations		X		Blood samples for PBMC preparations (pre-BAC and 6 hours after BAC at screening visit 2)
Provide diary card, PEF meters, rescue medication and thermometers to subjects		X		Only given out to subjects who are eligible following the BAC. Thermometers are to be given to record temperature as part of capturing AE information.
Nasal lavage			X	Collect nasal lavage to provide a background sample. Repeat nasal lavage pre-NAC, then again 5 minutes and at least 6 hours post-NAC.
Nasal filter collection			X	Collect nasal filter sample to provide a background pre-wash sample, after nasal wash repeat nasal filter sampling pre-NAC, then again 5 minutes and at least 6 hours post-NAC.
NAC			X	Commence after the 24-hour BAC sputum and blood biomarker samples have been taken.<br>Only to be done once subjects have completed a successful BAC.
ID challenge			X	ID challenge to be administered after NAC. Measurements to be made 15 minutes and 6 hours post ID challenge.
Nasal scrape			X	Nasal mucosa sample to be collected at least 6 hours post-NAC.

7.1.2. Time and Events Table – Dosing visit (DV) 1 (Day 1) – DV8 (Week 8) and Early Withdrawal Visit (EW)

<table>
<tr><th rowspan="2">Procedure</th><th rowspan="2">Pre-dose</th><th>Dose</th><th colspan="4">Post-dose</th><th rowspan="2">Early-Withdrawal</th><th rowspan="2">Notes</th></tr>
<tr><th>0 hour</th><th>20 mins</th><th>1 hour</th><th>12 hours</th><th>24 hours</th></tr>
<tr><td>Outpatient visit</td><td colspan="4">X</td><td></td><td>X</td><td>X</td><td>Subjects get discharged 1-2 hours after dosing.</td></tr>
<tr><td>Inclusion and exclusion criteria/eligibility review</td><td>X</td><td></td><td></td><td></td><td></td><td></td><td></td><td>Only on DV1</td></tr>
<tr><td>Diary card collection and review</td><td colspan="6">X</td><td>X</td><td>Collect and review diary card at every dosing visit</td></tr>
<tr><td>AE/SAE review</td><td colspan="6">X</td><td>X</td><td></td></tr>
<tr><td>Concomitant medication review</td><td colspan="6">X</td><td>X</td><td></td></tr>
<tr><td>12-lead ECG</td><td>X</td><td></td><td></td><td>X</td><td></td><td>X</td><td>X</td><td>Perform on DV1 and DV8 only.
Not required at EW if this occurs during FU</td></tr>
<tr><td>Vital signs (including temperature)</td><td>X[1]</td><td></td><td></td><td>X[1]</td><td></td><td>X[2]</td><td>X</td><td>[1] Perform on DV1-8
[2] Perform on DV1, 4 and DV8 only.
Not required at EW if this occurs during FU</td></tr>
<tr><td>Laboratory assessments (include liver chemistries, haematology, clinical chemistry and urinalysis)</td><td>X</td><td></td><td></td><td></td><td></td><td>X</td><td>X</td><td>Collect samples for laboratory assessments on DV1 and DV8,
Not required at EW if this occurs during FU</td></tr>
<tr><td>Blood sample for PK analysis</td><td>X</td><td></td><td>X</td><td>X</td><td></td><td></td><td></td><td>Collect samples on DV1, DV4 and DV8</td></tr>
<tr><td>Genetic sample</td><td colspan="6">X</td><td></td><td>Take one sample on any of the dosing visits after genetics consent.</td></tr>
<tr><td>FeNO measurement</td><td>X</td><td></td><td></td><td></td><td></td><td></td><td></td><td>Perform FeNO measurements pre-dose on all dosing visits 1-8</td></tr>
<tr><td>Nasal examination</td><td>X</td><td></td><td></td><td></td><td></td><td>X</td><td></td><td>Conducted pre-dose at all study visits and 24 hours post-dose on DV1, DV4 and DV8</td></tr>
</table>

Procedure	Pre-dose	Dose	Post-dose				Early-Withdrawal	Notes
		0 hour	20 mins	1 hour	12 hours	24 hours		
Nasal lavage	X					X		Collect nasal lavage samples pre-dose and 24hrs post-dose on DV1, DV4 and DV8
Blood sample for biomarker analysis	X					X		Collect blood for serum samples and PBMC preparations pre-dose and 24 hrs post-dose on DV1 and DV8
Blood sample for RNA analysis	X(3)					X(4)		PAX-gene tubes for TLR7/AFFY chip gene expression in blood collected pre-dose on DV1(3) and 24 hrs after DV8(4)
Call RAMOS	X							Randomisation will occur is at Visit 1 via the IRT. Sites should also call RAMOS at DV5.
Dispensing of study medication	X							One device will be assigned on DV1. The second device will be assigned on DV5. All devices remain in the unit.
Study drug administration		X						All doses are administered in the unit.
Sputum induction and processing for BAC associated sample collection						X		Sputum sample is conditional on having obtained a sputum sample from the subject at screening. Perform sputum induction after collection of 24 hour post-dose DV8 blood and nasal samples (as pre BAC sample for FUV1).
Follow up phone call					X[5]	X[6]		[5] Following at least the first two doses of study treatment to assess tolerability<br>[6] On DV 2, 3, and 5-7, a follow-up phone call will be made approximately 24 hours post-dose to check if any CRS events have occurred, and if they have, that they have been accurately recorded in the diary card and they will then be transcribed into the eCRF.<br>For DV1, 4 and 8 the subjects shall be assessed on their return to the unit the following day.

7.1.3. Time and Events Table – Follow up visits (FUV1 to 3) (Weeks 9 to 20)

Procedure	Follow up Visit 1 (FUV1)		Follow-up Visit 2 (FUV2)			2 month follow-up phone call (Week 16)	Follow up Visit 3 (FUV3)			Notes
	Week 9 (1 week after DV8)	24 hours later	Week 11 (3 weeks after DV8)	Week 12 (4 weeks after DV8)	24 hours later		Week 19 (11 weeks after DV8)	Week 20 (12 weeks after DV8)	24 hours later	
AE/SAE review	←====================================→					X[3]	←======================→			
Concomitant medication review	←====================================→					X[3]	←======================→			[3] Phone call made to the subject to check AEs and concomitant medications and confirm availability for FUV3
Diarycard collection, review and dispensing	X									Review diary card at every visit until the end of FUV 2
FeNO measurement	X	X		X	X			X	X	Collect prior to sputum induction
Blood sample for RNA analysis				X						PAX tubes for /AFFY chip gene expression in blood. Collected prior to the bronchial allergen challenge at FUV2
Saline and bolus BAC challenge plus associated spirometry measurements	X			X				X		Including pre-procedure FEV_1 assessment and repeat FEV_1 measurements up to 10 hours post BAC
Sputum induction and processing for BAC associated sample collection		X	X		X		X		X	Sputum induction pre-BAC and 24 hours after BAC
Laboratory assessments (haematology only)	X	X		X	X			X[1]	X	Blood samples should be taken pre-BAC and on the following day at 24hrs post BAC
Blood samples for PBMC preparations	X			X				X		Blood samples for PBMC preparations (pre-BAC and 6 hours after BAC)

Procedure	Follow up Visit 1 (FUV1)		Follow-up Visit 2 (FUV2)			2 month follow-up phone call (Week 16)	Follow up Visit 3 (FUV3)			Notes
	Week 9 (1 week after DV8)	24 hours later	Week 11 (3 weeks after DV8)	Week 12 (4 weeks after DV8)	24 hours later		Week 19 (11 weeks after DV8)	Week 20 (12 weeks after DV8)	24 hours later	
Nasal lavage		X			X					Collect nasal lavage to provide a background sample. Repeat nasal lavage pre-NAC, then again 5 minutes and at least 6 hours post-NAC.
Nasal filter collection		X			X					Collect nasal filter sample to provide a background pre-wash sample, after nasal wash repeat nasal filter sampling pre-NAC, then again 5 minutes and at least 6 hours post-NA.
NAC		X			X					Commence after the 24-hour BAC sputum and blood biomarker samples have been taken at FUV1 and FUV2
ID challenge		X			X					ID challenge to be administered after NAC.<br>Measurements to be made at 15 minutes and 6 hours post ID challenge.
Nasal scrape		X			X					Nasal mucosa sample to be collected at least 6 hours post-NAC
Return diary card and thermometers					X					
Return PEF meters and rescue medication									X	

7.2. Screening and Critical Baseline Assessments

Cardiovascular medical history/risk factors (as detailed in the CRF) will be assessed at screening.

The following demographic parameters will be captured: year of birth, sex, race and ethnicity.

Medical/medication/family history will be assessed as related to the inclusion/exclusion criteria listed in Section 5.

7.2.1. Skin prick testing

Seasonal allergen or perennial allergen-specific skin prick tests will be performed at screening to confirm eligibility, and to determine the allergen to be used for each subject for subsequent BAC, NAC and ID challenge.

7.3. Efficacy

7.3.1. Bronchial allergen challenges and spirometry

BAC will be conducted at screening, and at three follow-up visits (see Time and Events Tables in Section 7.1). At the Screening Visit incremental doses of allergen will be administered and the total dose inducing an EAR and LAR used to calculate a bolus dose for use at the 3 FU visits. A detailed description of the BAC technique and assessments can be found in the SRM. All sites participating in the study will be trained in the same methodology to ensure consistency.

Following the final FEV_1 measurement, subjects will receive rescue salbutamol until the FEV_1 returns to within 10% of the pre-saline value. If a subject experiences serious discomfort the test will be discontinued and rescue medication administered.

7.3.2. Intranasal and intradermal allergen challenge

NAC and ID challenge will be conducted approximately 24 hours after the bronchial allergen challenge at screening and at follow-up visits 1 and 2.

A detailed description of the NAC and ID challenges can be found in the SRM.

In some regions/countries specific challenges agents may not be approved or available for use in ID challenge, in which case this assessment shall be omitted.

7.3.3. Sputum induction

Hypertonic saline induced sputum samples will be collected. The sputum induction process will follow a standard procedure and GSK will ensure all sites are trained prior to study start in order to provide consistency.

At screening, the weight of the sputum sample will be measured to confirm that the subject is able to produce an adequate induced sputum sample (must be at least 100 mg).

If a subject did not induce a successful sputum sample at screening then they will not be required to try at the follow up visits.

Further information on induction, collection, processing, storage and shipping procedures are provided in the SRM.

7.3.4. TNSS

Four nasal symptoms: nasal congestion, rhinorrhea, nasal itch and sneezing will be evaluated on the morning and evening of each day of the study treatment period, from DV1 up until follow-up visit 2 (4 weeks after the end of treatment). These will be captured on a diary card every morning and evening. On dosing days, the morning TNSS assessment will be captured pre-dose. The subjects will be asked to rate each of these symptoms on a 4-point scale:

0 = None: No symptom whatsoever; Absent

1 = Mild: Symptom is present, noticeable but not bothersome

2 = Moderate: Symptom is bothersome, but tolerable

3 = Severe: Symptom which are bothersome, harder to tolerate, desiring relief/treatment

The individual symptom scores will be combined to produce a TNSS.

All data will be transcribed from the diary booklet into the CRF.

7.3.5. Nasal examination

A brief visual nasal examination will be conducted by a trained physician at the time-points outlined in the Time and Events Tables (Section 7.1). The physician will examine the nostrils using appropriate instrumentation (e.g. a small rounded plastic nasal speculum and an auroscope fitted with a wide bore examination probe) for any signs of irritancy. Any abnormalities will be documented in the CRF.

7.3.6. Fractional Exhaled Nitric Oxide (FeNO)

FeNO will be measured using a handheld electronic device. Measurements will be obtained in accordance with the [ATS/ERS Recommendations for Standardized Procedures for the Online and Offline Measurement of Exhaled Lower Respiratory Nitric Oxide and Nasal Nitric Oxide, 2005]. FeNO measurements will be interpreted in accordance with the [Official ATS Clinical Practice Guideline: Interpretation of Exhaled Nitric Oxide Levels (FeNO) for Clinical Applications, 2011].

Further details will be provided in the SRM.

7.4. Safety

Planned time points for all safety assessments are listed in the Time and Events Table (Section 7.1). Additional time points for safety tests (such as vital signs, physical exams

and laboratory safety tests) may be added during the course of the study based on newly available data to ensure appropriate safety monitoring.

The Safety Review Team (SRT) is a GSK cross-functional team reviewing all available safety data related to the project, including in-stream data from this study, in an ongoing manner. The SRT is an internal GSK requirement put in place to ensure holistic evaluation of the safety profile of an investigational product with systematic, periodic and documented reviews of available safety data, with the appropriate communication and escalation of new findings that have the potential to impact patient safety.

The SRT for this project will review data from this study in collaboration with an independent external (to GSK) expert reviewer.

7.4.1. Adverse Events (AE) and Serious Adverse Events (SAEs)

The definitions of an AE or SAE can be found in Appendix 4.

The investigator and their designees are responsible for detecting, documenting and reporting events that meet the definition of an AE or SAE.

7.4.1.1. Time period and Frequency for collecting AE and SAE information

Any SAEs assessed as related to study participation (e.g., protocol-mandated procedures, invasive tests, or change in existing therapy) or related to a GSK product will be recorded from the time a subject consents to participate in the study up to and including the final follow-up visit.

AEs will be collected from the start of Study Treatment until the final follow-up visit (see Section 7.4.1.3), at the time-points specified in the Time and Events Table (Section 7.1).

Medical occurrences that begin prior to the start of study treatment but after obtaining informed consent may be recorded on the Medical History/Current Medical Conditions section of the CRF.

All SAEs will be recorded and reported to GSK within 24 hours, as indicated in Appendix 4.

Investigators are not obligated to actively seek AEs or SAEs in former study subjects. However, if the investigator learns of any SAE, including a death, at any time after a subject has been discharged from the study, and he/she considers the event reasonably related to the study treatment or study participation, the investigator must promptly notify GSK.

NOTE: The method of recording, evaluating and assessing causality of AEs and SAEs plus procedures for completing and transmitting SAE reports to GSK are provided in Appendix 4.

7.4.1.2. Method of Detecting AEs and SAEs

Care will be taken not to introduce bias when detecting AEs and/or SAEs. Open-ended and non-leading verbal questioning of the subject is the preferred method to inquire about AE occurrence. Appropriate questions include:

> "How are you feeling?"
>
> "Have you had any (other) medical problems since your last visit/contact?"
>
> "Have you taken any new medicines, other than those provided in this study, since your last visit/contact?

7.4.1.3. Follow-up of AEs and SAEs

After the initial AE/SAE report, the investigator is required to proactively follow each subject at subsequent visits/contacts. All SAEs, and non-serious AEs of special interest (as defined in Section 7.4.1.5) will be followed until resolution, until the condition stabilizes, until the event is otherwise explained, or until the subject is lost to follow-up (as defined in Section 5.4). Further information on follow-up procedures is given in Appendix 4.

7.4.1.4. Cardiovascular and Death Events

Investigators will be required to fill out event specific data collection tools for the following AEs and SAEs:

Myocardial infarction/unstable angina
Congestive heart failure
Arrhythmias
Valvulopathy
Pulmonary hypertension
Cerebrovascular events/stroke and transient ischemic attack
Peripheral arterial thrombosis
Deep Venous Thrombosis
Revascularization

For these cardiovascular events detailed above and for all deaths, whether or not they are considered SAEs, specific Cardiovascular (CV) and Death sections of the CRF will berequired to be completed. These sections include questions regarding cardiovascular (including sudden cardiac death) and non-cardiovascular death.

The CV CRFs are presented as queries in response to reporting of certain CV Medical Dictionary for Regulatory Activities (MedDRA) terms. The CV information should be recorded in the specific cardiovascular section of the CRF within one week of receipt of a CV Event data query prompting its completion.

The Death CRF is provided immediately after the occurrence or outcome of death is reported. Initial and follow-up reports regarding death must be completed within one week of when the death is reported.

7.4.1.5. Disease-Related Events and/or Disease-Related Outcomes Not Qualifying as SAEs

The following disease related events (DREs) are common in subjects with mild allergic asthma and can be serious/life threatening:

- Wheeziness
- Chest tightness or chest heaviness
- Coughing

Because these events are typically associated with the disease under study, they will not be reported according to the standard process for expedited reporting of SAEs to GSK (even though the event may meet the definition of a SAE). These events will be recorded on the DRE page in the subject's CRF within 5 working days.

NOTE: However, if either of the following conditions apply, then the event must be recorded and reported as an SAE (instead of a DRE):

The event is, in the investigator's opinion, of greater intensity, frequency, or duration than expected for the individual subject, or

The investigator considers that there is a reasonable possibility that the event was related to treatment with the investigational product.

7.4.1.6. Regulatory Reporting Requirements for SAEs

Prompt notification by the investigator(s) to GSK of SAEs related to study treatment (even for non- interventional post-marketing studies) is essential so that legal obligations and ethical responsibilities towards the safety of subjects and the safety of a product under clinical investigation are met.

GSK has a legal responsibility to notify both the local regulatory authority and other regulatory agencies about the safety of a product under clinical investigation. GSK will comply with country specific regulatory requirements relating to safety reporting to the regulatory authority, Institutional Review Board (IRB)/Independent Ethics Committee (IEC) and investigators.

Investigator safety reports are prepared for suspected unexpected serious adverse reactions according to local regulatory requirements and GSK policy and are forwarded to investigators as necessary.

An investigator who receives an investigator safety report describing a SAE(s) or other specific safety information (e.g., summary or listing of SAEs) from GSK will file it with the IB and will notify the IRB/IEC, if appropriate according to local requirements.

7.4.1.7. Cytokine Related Symptoms (CRS) - Symptoms Grading

In the study, symptoms potentially related to cytokine induction (headache, chills/rigors, fever, nausea, vomiting, diarrhoea, arthralgia, myalgia, hypotension) occurring within 24 hours of dosing will be individually reported as AEs and graded as described in Section 12.4.2 in Appendix 4.

7.4.2. Pregnancy

Details of all pregnancies in female subjects and female partners of male subjects will be collected after the start of dosing until the final follow-up visit.

If a pregnancy is reported then the investigator should inform GSK within 2 weeks of learning of the pregnancy and should follow the procedures outlined in Appendix 5.

7.4.3. Physical Exams

A complete physical examination will include, at a minimum, assessment of the Cardiovascular, Respiratory, Gastrointestinal and Neurological systems. Height and weight will also be measured and recorded.

A brief physical examination will include, at a minimum assessments of the skin, lungs, cardiovascular system, and abdomen (liver and spleen).

Investigators should pay special attention to clinical signs related to previous serious illnesses

7.4.4. Vital Signs

Vital signs will be measured in semi-supine position after 5 minutes rest and will include temperature, systolic and diastolic blood pressure, pulse rate and respiratory rate.

Measurements will be made at screening and various occasions throughout the study as outlined in the Time and Events Tables (Section 7.1). Measurements that deviate substantially from normal range, according to the opinion of the Investigator(s), will be repeated as soon as possible.

7.4.5. Electrocardiogram (ECG)

Triplicate 12-lead ECGs will be obtained at screening. Single ECGs will be obtained at the other determined time points during the study except if according to the opinion of the investigator(s) there are abnormal findings and therefore triplicate ECGs should be obtained. These measurements will be obtained at each timepoint during the study using an ECG machine that automatically calculates the heart rate and measures PR, QRS, QT, and QTc intervals. Refer to Section 5.4.2 for QTc withdrawal criteria and additional QTc readings that may be necessary.

7.4.6. Clinical Safety Laboratory Assessments

All protocol required laboratory assessments must be conducted in accordance with the Laboratory Manual, and Protocol Time and Events Schedule. Laboratory requisition forms must be completed and samples must be clearly labelled with the subject number, protocol number, site/centre number, and visit date. Details for the preparation and shipment of samples will be provided by the laboratory and are detailed in the laboratory manual. Reference ranges for all safety parameters will be provided to the site by the laboratory responsible for the assessments.

If additional non-protocol specified laboratory assessments are performed at the institution's local laboratory and result in a change in subject management or are considered clinically significant by the investigator (e.g., SAE or AE or dose modification) the results must be recorded in the CRF.

Refer to the SRM for appropriate processing and handling of samples to avoid duplicate and/or additional blood draws.

All study-required laboratory assessments will be performed by a central laboratory.

NOTE: Local laboratory results are only required in the event that the central laboratory results are not available in time for either a treatment and/or response evaluation to be performed. If a local sample is required it is important that the sample for central analysis is obtained at the same time. Additionally if the local laboratory results are used to make either a treatment or response evaluation, the results must be entered into the CRF.

Haematology, clinical chemistry, urinalysis and additional parameters to be tested are listed in Table 1.

Table 1 Protocol Required Safety Laboratory Assessments

Laboratory Assessments	Parameters			
Haematology	Platelet Count	*RBC Indices*:	*White Blood Cell (WBC) count with Differential:*	
	Red Blood Cell (RBC) Count	Mean Corpuscular Volume (MCV)	Neutrophils	
	Hemoglobin	Mean corpuscular hemoglobin (MCH)	Lymphocytes	
	Hematocrit		Monocytes	
			Eosinophils	
			Basophils	
Clinical Chemistry [1]	Blood Urea Nitrogen (BUN)	Potassium	AST (SGOT)	Total and direct bilirubin
	Creatinine	Sodium	ALT (SGPT)	Total Protein
	Glucose	Calcium	Alkaline phosphatise	Albumin
Routine Urinalysis	• Specific gravity<br>• pH, glucose, protein, blood and ketones by dipstick<br>• Microscopic examination (if blood or protein is abnormal)			
Other Screening Tests	• HIV<br>• Hepatitis B (HBsAg)<br>• Hepatitis C (Hep C antibody)<br>• FSH and estradiol (as needed in women of non-child bearing potential only)<br>• Alcohol and drug screen (to include at minimum: amphetamines, barbiturates, cocaine, opiates, cannabinoids and benzodiazepines)<br>• Urine hCG Pregnancy test [2]			

NOTES :

1. Details of Liver Chemistry Stopping Criteria and Required Actions and Follow-Up Assessments after liver stopping or monitoring event are given in Section 5.4.1 and Appendix 2.
2. Local urine testing will be standard for the protocol unless serum testing is required by local regulation or ethics committee.

All laboratory tests with values that are considered clinically significantly abnormal during participation in the study should be repeated until the values return to normal or baseline. If such values do not return to normal within a period judged reasonable by the investigator, the etiology should be identified and the sponsor notified.

7.4.7. PEF monitoring

Subjects will be given a PEF peak flow meter at screening, along with a diary card. This will be used for safety monitoring after the BACS. Subjects will record their PEF readings each morning and evening onto the diary card every day from the start of study treatment on DV 1 until the 4 week follow-up visit.

The subject will be instructed to contact the investigator if at any time during the study the PEF measurements go outside the normal range for the subject. These instructions will be clearly printed on the diary card.

All information from the diary card will be transcribed into the eCRF.

7.5. Pharmacokinetics

7.5.1. Blood Sample Collection

Blood samples for pharmacokinetic (PK) analysis of GSK2245035 will be collected at the time points indicated in Section 7.1, Time and Events Tables. The actual date and time of each blood sample collection will be recorded. The timing of PK samples may be altered and/or PK samples may be obtained at additional time points to ensure thorough PK monitoring.

Processing, storage and shipping procedures are provided in the SRM.

7.5.2. Sample Analysis

Plasma analysis will be performed under the control of Platform Technology Sciences (PTS)-DMPK/Scinovo, GlaxoSmithKline, the details of which will be included in the Study Reference Manual (SRM). Concentrations of GSK2245035 will be determined in plasma samples using the currently approved bioanalytical methodology. Raw data will be archived at the bioanalytical site (detailed in the SRM).

Once the plasma has been analyzed for GSK2245035, any remaining plasma may be analyzed for other compound-related metabolites and the results reported under a separate PTS-DMPK/Scinovo, GlaxoSmithKline protocol.

7.6. Exploratory Biomarkers

7.6.1. Pharmacodynamic Markers

Details for the collection of samples, processing, storage and shipping procedures for all samples are provided in the SRM. Time-points for all sample collections are shown in the Time and Events Tables (Section 7.1).

7.6.1.1. PD blood samples

Blood samples will be collected before and 24 hours post treatment on DV 1 and 8 for serum to measure blood PD biomarkers.

Blood will be collected before and 24 hours after treatment on DV 1 and 8 and prior to bronchial allergen challenges for preparation of peripheral blood mononuclear cells (PBMC). These samples may be used for CHIP cytometry for cellular subset phenotyping. If this biomarker analysis is performed it may be reported separately.

7.6.1.2. Blood PAX collection

Blood PAX samples for AFFYCHIP gene expression (mRNA transcriptome) will be collected before dosing on DV 1, 24 hours after DV 8 and prior to BAC on FUV2.

7.6.1.3. PD nasal lavage samples

Nasal lavage samples to measure PD biomarkers will be collected before and 24 hours post treatment on DV1, DV4 and DV8.

7.6.1.4. Bronchial allergen challenge (BAC) associated biomarkers in induced sputum samples

Sputum samples will be collected at the time points indicated in the time and events tables (see Section 7.1). Sputum sample collection at dosing/follow up visits is conditional on having obtained a sputum sample from the subject at screening.

Detailed information on collection, processing, storage and shipping procedures are provided in the SRM.

7.6.1.5. Bronchial allergen challenge (BAC) associated biomarkers in blood samples

Blood samples will be collected before, 6 hours and 24 hours after each BAC:

For haematology, (WBC with differential) for blood eosinophil counts (before and 24 hours after BAC)

To prepare PBMC that may be used for CHIP cytometry (before and 6 hours after BAC). If this biomarker analysis is performed it may be reported separately.

7.6.1.6. Nasal allergen challenge associated biomarkers in nasal fluid samples

Nasal filter and nasal lavage samples to measure exploratory biomarkers will be collected at the timepoints indicated in the Time and Events Tables, Section 7.1.

7.6.1.7. Cellular profile of nasal mucosa

Nasal scrapes may be collected after NAC following the completion of the collection of the 6 hour nasal lavage and nasal filter samples (at screening and at FUV1 and FUV2). Samples of suitable quality may be used for cellular analysis for example by CHIP cytometry or RNA-sequencing of single cells (if subjects have given informed consent for genetic analysis techniques). If this biomarker analysis is performed it may be reported separately.

7.6.2. Novel Biomarkers

With the subject's consent, nasal tissue, nasal fluid, sputum and blood samples will be collected during this study and may be used for the purposes of measuring novel biomarkers to identify factors that may influence the disease for study treatment, and/or medically related conditions, as well as the biological and clinical responses to GSK2245035. If relevant, this approach will be extended to include the identification of biomarkers associated with adverse events.

Samples will be collected at the time points indicated in the Time and Events Table Section 7.1. The timing of the collections may be adjusted on the basis of emerging pharmacokinetic or pharmacodynamic (PD) data from this study or other new information in order to ensure optimal evaluation of the PD endpoints.

Novel candidate biomarkers and subsequently discovered biomarkers of the biological response associated with asthma or medically related conditions and/or the action of GSK2245035 may be identified by application of:

Measurement of the levels of a subset of RNA species on blood and sputum samples.

Proteome analysis of plasma, nasal and sputum samples.

All samples will be retained for a maximum of 15 years after the last subject completes the trial.

7.7. Genetics

Information regarding genetic research is included in Appendix 3.

8. DATA MANAGEMENT

For this study, subject data will be entered into GSK defined CRFs, transmitted electronically to GSK or designee and combined with data provided from other sources in a validated data system.

Paper diary subject data will be collected and transcribed into GSK defined CRFs by the site personnel.

Management of clinical data will be performed in accordance with applicable GSK standards and data cleaning procedures to ensure the integrity of the data, e.g., removing errors and inconsistencies in the data.

Adverse events and concomitant medications terms will be coded using MedDRA (Medical Dictionary for Regulatory Activities) and an internal validated medication dictionary, GSKDrug.

CRFs (including queries and audit trails) will be retained by GSK, and copies will be sent to the investigator to maintain as the investigator copy. Subject initials will not be collected or transmitted to GSK according to GSK policy.

9. STATISTICAL CONSIDERATIONS AND DATA ANALYSES

9.1. Hypotheses

No formal hypotheses will be tested. The primary study objective will assess the effect of a weekly dose of 20ng GSK2245035 vs Placebo by precision estimation and credible intervals to estimate the magnitude of underlying effect sizes. A Bayesian approach will be utilised to obtain the posterior distribution for the mean treatment effects for the endpoints in the study (e.g. posterior distributions for percentage attenuation for the endpoint LAR: minimum FEV_1 between 4-10 hours following allergen challenge one week after treatment.).

Other objectives will assess other pharmacodynamic effects and safety and tolerability of GSK 2245035.

9.2. Sample Size Considerations

The following was performed for each of the proposed LAR primary endpoints:

Success criteria: declare endpoint success if the posterior probability of any treatment attenuation exceeds 0.7.

The probability of declaring a success has been computed for a range of true percent attenuations and sample sizes (n per arm equal allocation). To make the figures easier to interpret the “nuisance” parameters of the placebo response and the standard deviation have been accounted for as follows:

Eight previous GSK BAC studies (Crossover design) have been re-analysed to obtain estimates of the average placebo response and the associated estimate of residual variability applicable to the proposed Parallel Group design.

These estimates were combined in a random effects meta-analysis to obtain posterior distributions for the underlying parameter values (placebo average response, residual variability (for an individual subject) and a between study variance component). The future placebo response was computed as the sum of random draws from the posterior distributions of the placebo average response parameter and a normal distribution with mean zero and variance equal to the between study variance parameter. The future GSK2245035 response was derived using the simulation scenario’s true percentage attenuation. A pooled sample variance value was generated using a Chi-Square distribution with degrees of freedom appropriate to the simulation scenario and a sampled variance from the meta-analysis posterior distribution. Those numbers were entered into the standard formulae for power curves. Repeating this process 10,000 times and taking the median of the resulting power values constructs “Assurance” operational characteristics for the combination of true percent attenuation and sample size under evaluation.

These assurance operational curves have been summarised graphically. The x-axis is a hypothetical true drug effect that is assumed to be known for experimental design purposes.

The simulations can also be used to compute the minimum observed percentage attenuations that would result in a successful study outcome. The median (of the 10,000 simulations) have been derived for a variety of sample sizes and success criteria (probability thresholds 0.7, 0.75, 0.8, 0.85, 0.95) and displayed graphically. The y-axis is the observed effect from the study data.
Endpoint: LAR: minimum FEV_1 between 4-10 hours following allergen challenge

Figure 1 illustrates the operating characteristics for the proposed design (n=20 completers per arm) that have a reasonable chance of meeting the definition of success.

The best guess for the true treatment effect comes from the AZ study with AZD8848 in subjects with mild asthma, which dosed out of the allergen season and observed an approximately 18% effect size (Figure 2) [Leaker, 2012].

Figure 1 LAR: minimum FEV1 between 4-10 hours following allergen challenge: Probability of Success (Assurance) with Success threshold of 0.7 (akin to a risk of false positives of 30%)

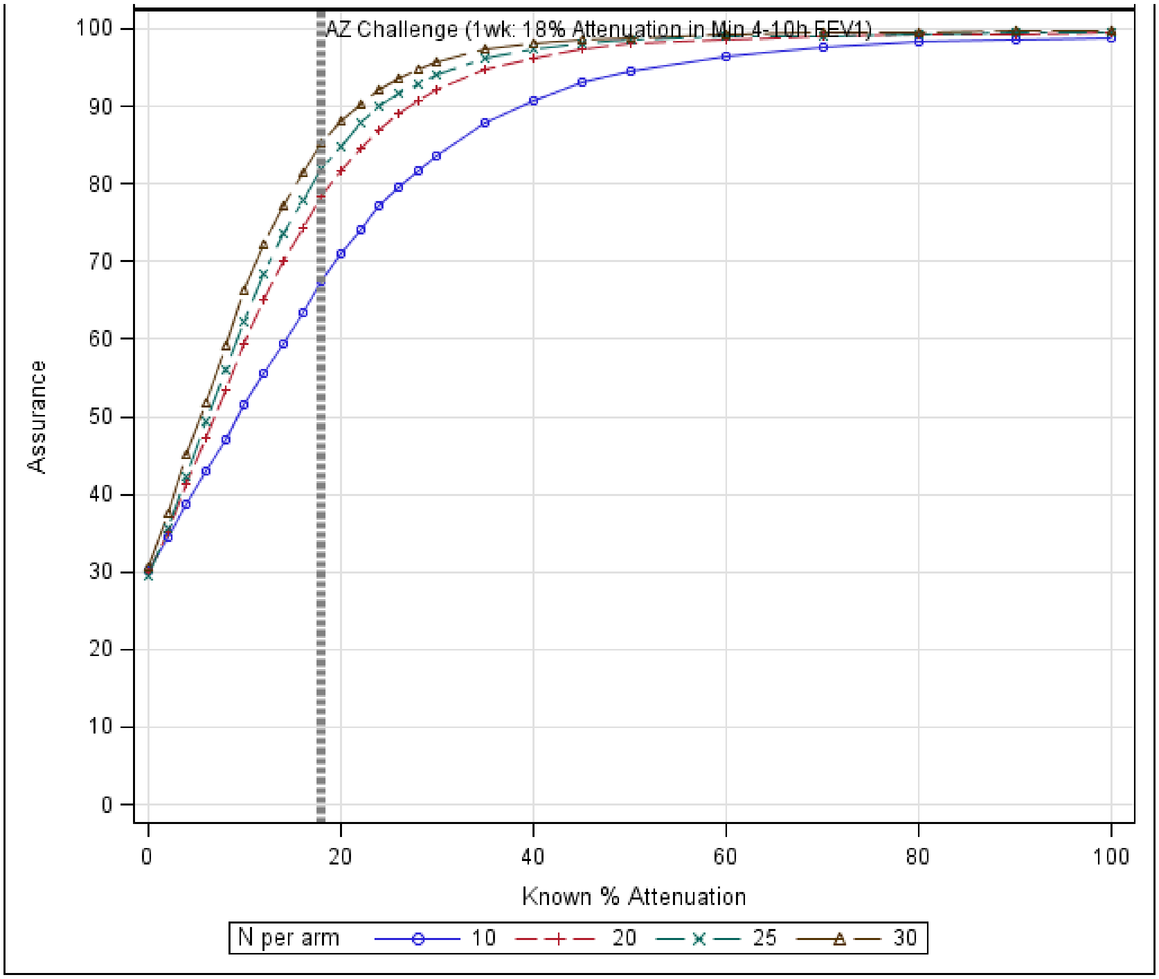

Figure 2 **LAR: minimum FEV1 between 4-10 hours following allergen challenge: Minimum observed % attenuations needed to declare success for a variety of sample sizes and Success thresholds (levels of false positive risk)**

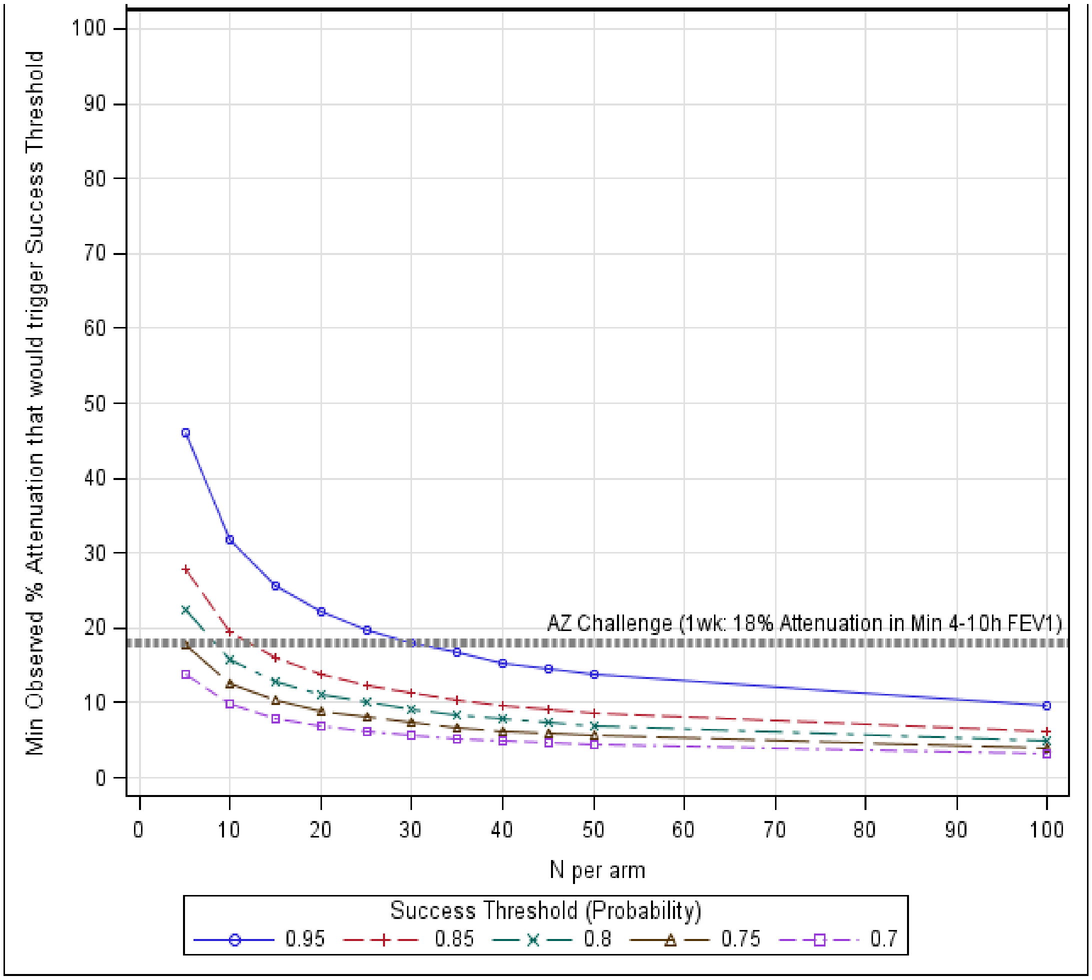

- Endpoint: LAR: weighted mean FEV_1 between 4-10 hours following allergen challenge

Figure 3 and Figure 4 are analogous to Figure 1 and Figure 2. The corresponding AZ point estimate was 27% attenuation. Similar conclusions can be drawn.

Figure 3 **<u>LAR: weighted mean FEV1 between 4-10 hours following allergen challenge</u>: Probability of Success (Assurance) with Success threshold of 0.7 (akin to a risk of false positives of 30%)**

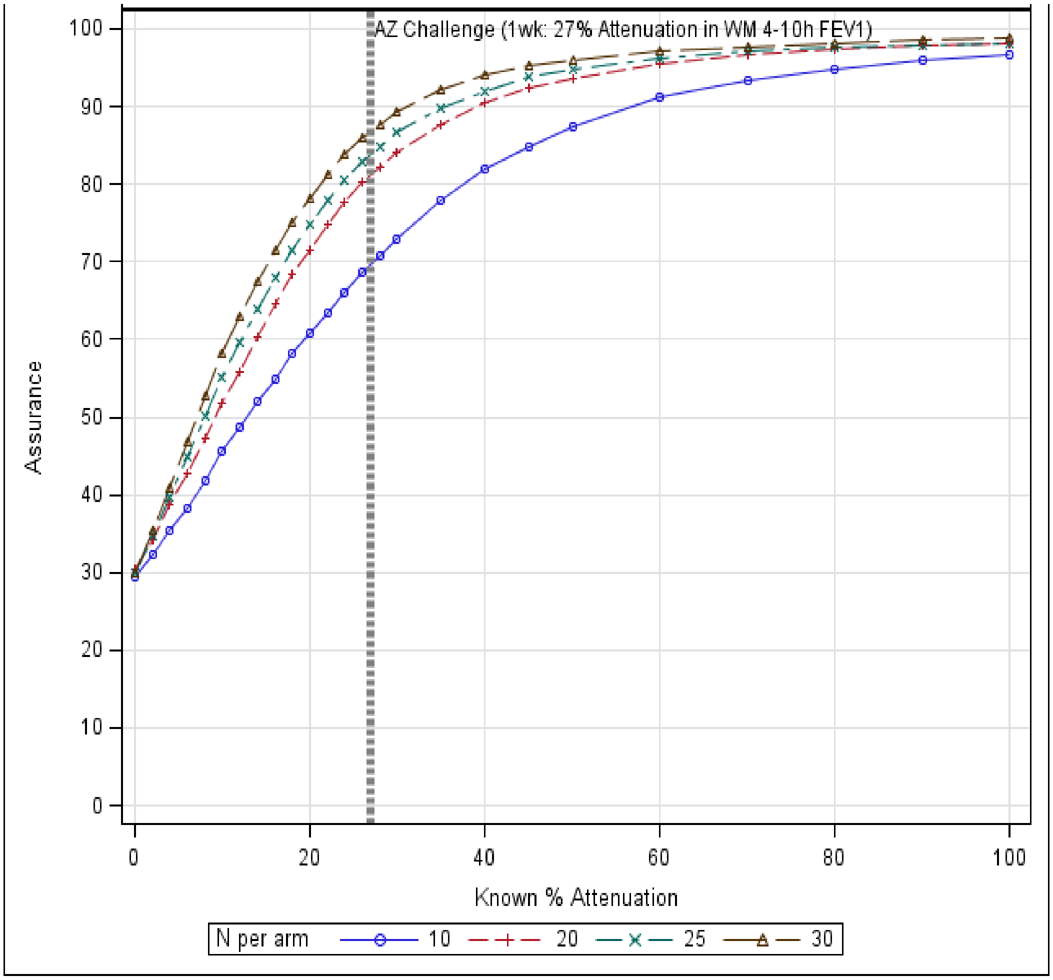

Figure 4 **LAR: weighted mean FEV1 between 4-10 hours following allergen challenge: Minimum observed % attenuations needed to declare success for a variety of sample sizes and Success thresholds (levels of false positive risk)**

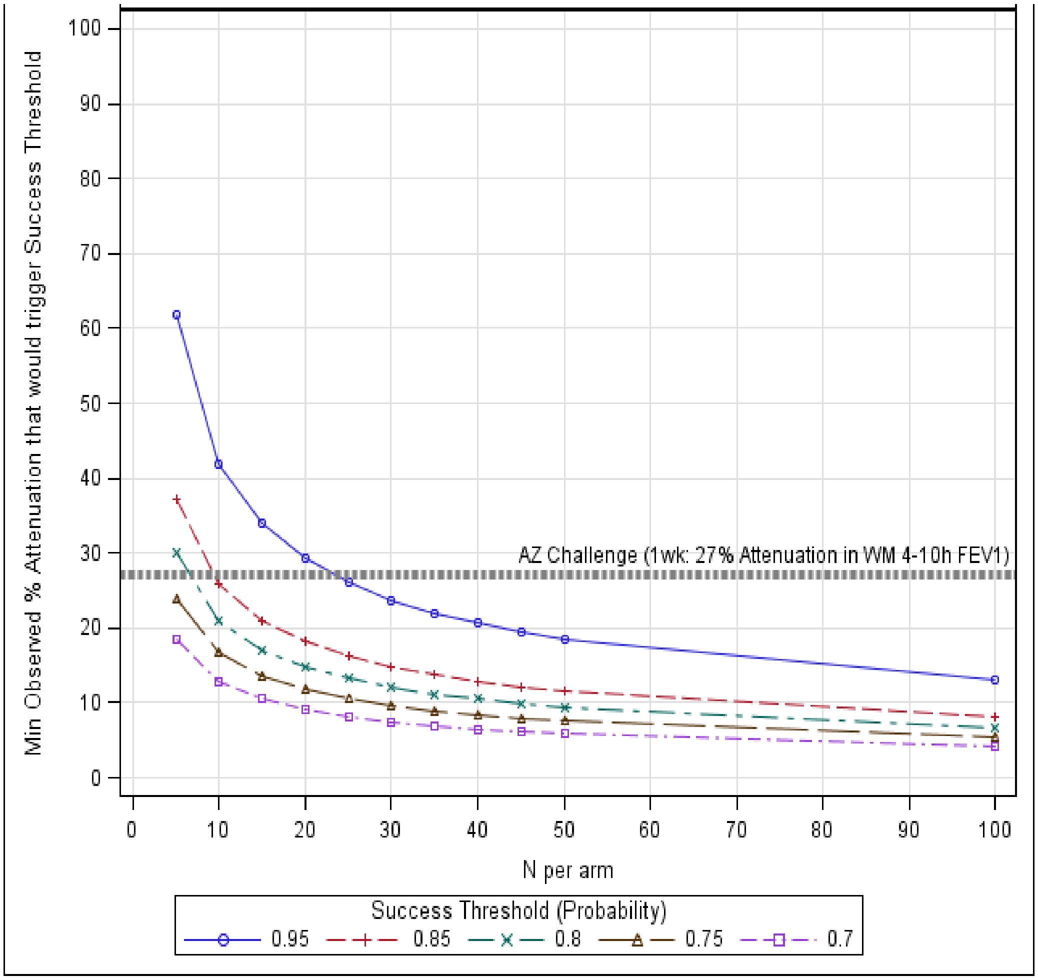

9.2.1. Sample Size Assumptions

The sample size assumes that the behaviour of the placebo response and the variability of the endpoints in this study are similar to the eight historical GSK studies (Table 2).

Table 2 Input data for Sample Size Work

	LAR Minimum FEV1 between 4-10h			LAR Weighted Mean FEV1 between 4-10h		
GSK Internal Study ID	Average Placebo Response	Degrees of Freedom	Residual Variability	Average Placebo Response	Degrees of Freedom	Residual Variability
el110002	-1.3975	33.5	0.40571	-0.9462	33.1	0.30417
ino102141	-1.0864	45.9	0.13063	-0.7093	42.3	0.11430
ipa101985	-1.1238	22.9	0.30521	-0.6916	22.8	0.18820
res104385	-0.9006	48.9	0.07344	-0.3749	47.9	0.02191
hza113126	-0.7309	85.3	0.10717	-0.4662	84.4	0.07528
sig110762	-0.9027	31.9	0.27740	-0.5502	34.0	0.20925
lpa111834	-1.4484	20.4	0.40528	-0.9817	20.3	0.31342
res114748	-0.8129	69.6	0.15866	-0.5260	71.8	0.08695

Assuming the true treatment effect of GSK2245035 is no worse than the results from the AstraZeneca study and 20 subjects complete follow up assessments the probability of meeting the success criteria for Min LAR and WM LAR are approximately 78% and 81% respectively. This is considered sufficient justification for requiring 20 completers.

9.2.2. Sample Size Sensitivity

Probability of success (Assurance) was determined for all combinations of these three factors: success thresholds of 0.95, 0.85, 0.8, 0.75 and 0.7, sample sizes (per arm) of 5, 10, 15, 20, 25, 30, 35, 40, 45, 50, 100 and Known % attenuations of 0% to 30% by 2% increments, 35 to 50% by 5% increments and 60% to 100% by 10% increments. Full results are available on file.

9.2.3. Sample Size Re-estimation or Adjustment

No sample size re-estimation is currently planned for this study beyond that described in Section 9.3.2. However, if during the course of the study, new information becomes available about clinically meaningful differences or variability estimates, a sample size re-estimation may be conducted.

9.3. Data Analysis Considerations

9.3.1. Analysis Populations

Subjects who receive at least one dose of study medication will be included in an ‘All Subjects’ population. Furthermore, subjects in the ‘All Subjects’ population who have a successful screening sputum sample will be included in the ‘Sputum Producers’ population.

Additionally, subjects from the All Subjects population who were not on Placebo and who provide GSK2245035 pharmacokinetic samples will be included in a ‘PK’ population.

Other populations, such as a Per-protocol population, may be defined in the RAP.

9.3.2. Interim Analysis

Recruitment into the study would continue whilst the interim analysis is taking place.

The interim analysis will require knowledge of which subjects are in the Placebo arm and determine how many of those Placebo subjects failed to meet the screening inclusion criteria for LAR in their 1 week follow up visit (i.e. did not show three FEV_1 decreases of ≥ 15 % relative to saline baseline between 4 and 10 hours, with two FEV_1 decreases being at consecutive time points at 1 week follow up). The number of subjects on placebo who do not exhibit an LAR (non-LAR responders) will be determined from the available interim data. This number will be checked against Table 3 to advise whether the study should continue or whether further review of the interim analysis data is required to assess the impact. For example, if at the time the interim analysis occurs 10 subjects were on Placebo and 4 of those 10 did not demonstrate an LAR response then the interim analysis would recommend reviewing the data further.

Table 3 Lookup table for Interim Analysis Decision Pathway

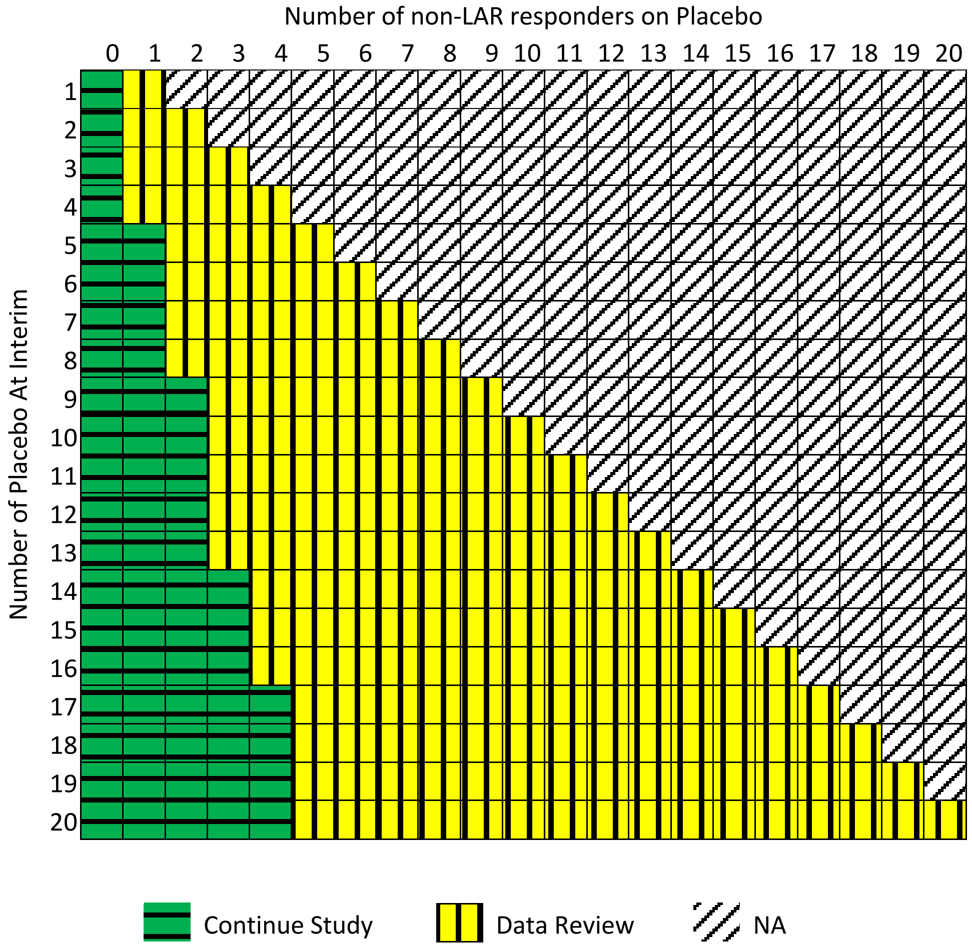

Assessing impact of non-LAR response rate: To evaluate whether recruiting additional subjects may counteract the impact of the non-LAR responder alternative version(s) of Table 3 would be constructed, but using final sample sizes that range from 20 to 30 subjects per arm. If any of the resulting estimates for probability of success are within 10% of the original operating characteristic the study may continue; but requiring additional subjects (the smallest number that satisfied the continuation criteria).
In addition posterior distributions for the percentage attenuation of the primary endpoints by GSK2245035 would be constructed from the available interim data.

Amending protocol / termination of further enrolment (existing subjects would complete the study as planned): If the median (point estimate) for the observed treatment effect is dramatically different to the assumed effect from the AZ study, the study team may wish to predict the probability of success at the end of the study from the interim information (details to be provided in the RAP). If this updated probability of success is low then the study team may review any of the other available data items at the interim prior to amending the protocol / terminating further enrolment (and share these outputs with appropriate internal GSK governance boards / senior management).

To determine the probability of success in the presence of non-LAR responders (for each of the primary endpoints) simulations were performed for each possible combination of number of available placebo subjects (n) and number showing no LAR response (r) using the AZ point estimates as the true percentage attenuation:

The rate of non-LAR response (p) was assumed to be the same for Placebo and GSK2245035 arms. A Beta(0.1, 0.1) prior distribution was assumed for the rate, which was updated using conjugate formulae as Beta(0.1 + r, 0.1 + n – r) and a value sampled from this posterior distribution. The number of future subjects on placebo with a non-LAR response was sampled from a Binomial distribution using the sampled value of p and (20 – n) observations (the total is a mixture of observed interim data and this sampled value). Similarly the number of non-LAR responders on the GSK2245035 arm was sampled from a Binomial distribution with the same value of p and 20 observations. Each non-responding individual was assigned a constant value for the response, and the collection of non-responders were combined with the collection of LAR responders (generated in a similar way using the random effects meta-analysis to obtain the expected responses). The probability of success was determined for each of the combined datasets and the median was derived. If this median was more than 10% smaller than the original operating characteristic the recommendation is to further review the data. The recommendations from the two endpoints were combined into a single table. Wherever there was disagreement between cells the review data decision was chosen.

Sensitivity analyses showed the outcome of this approach is highly dependent on the value of the constant used for the non-LAR responses (the further the choice from the range of plausible values for LAR responders the more likely the recommendation was to review data). The choice of value is arbitrary in nature, but was selected as -0.45 for Minimum Change From Saline and -0.2 for Weighted Mean Minimum Change From Saline, based upon applying the LAR criteria to seven historical studies and deriving the mean responses of all non-LAR responding subjects. Since the decision tables cover all interim study possibilities and is pre-specified the consequences of this arbitrary choice can be articulated.

9.3.2.1. Headline results for LAR endpoints at Follow Up 1

The following is conditional on the study not being stopped early by the interim analysis. To facilitate internal GSK decision making a data snapshot will be taken when the last subject's BAC FEV_1 data (and derived LAR endpoints) from Follow Up 1 are available. The associated planned efficacy outputs (to be provided in the RAP) will be produced from that (unblinded) snapshot and shared internally with the GSK study team and internal GSK governance boards / senior management. This data snapshot will not represent a cleaned and locked database and hence its results will not be formally reported as they will be superseded by the formal end of study results.

9.4. Key Elements of Analysis Plan

9.4.1. Primary Analyses

Statistical models will be fitted separately to each LAR endpoint (weighted mean and minimum FEV_1). These parameters will be analysed using analysis of covariance (ANCOVA) with treatment and baseline in the model; other covariates such as centre, allergen exposure ('presumed'/'unknown') (see Section 6.2) and allergen exposure by treatment interaction may be included in the model if the observed data support it (the default position is not to include the model terms involving the stratification factor). Treatment comparisons of GSK2245035 vs Placebo will be presented with results also expressed as percentage attenuations to aid clinical interpretation. The posterior probability of any percentage attenuation will be obtained along with appropriate summary statistics from the posterior distribution (e.g. 95% and 70% equi-tail Credible Intervals).

The principle inferences are expected to come from models with non-informative priors. Sensitivity analyses using informative priors based on AZ data may be fitted.

Full details of the BAC analyses will be provided in the RAP.

9.4.2. Secondary Analyses

Weighted mean and minimum for EAR will be analysed in a similar manner to that of the primary analysis described in Section 9.4.1.

9.4.3. Safety Analyses

Safety and tolerability data, such as rescue medication, PEF, AEs, will be displayed in graphical or summary format or listed as appropriate. Additional details will be provided in the RAP.

9.4.4. Exploratory Analyses

Pharmacokinetic data will be presented in graphical or tabular form and will be summarized descriptively.

Other exploratory analyses will be described in detail in the RAP.

10. STUDY GOVERNANCE CONSIDERATIONS

10.1. Posting of Information on Publicly Available Clinical Trial Registers

Study information from this protocol will be posted on publicly available clinical trial registers before enrollment of subjects begins.

10.2. Regulatory and Ethical Considerations, Including the Informed Consent Process

Prior to initiation of a site, GSK will obtain favourable opinion/approval from the appropriate regulatory agency to conduct the study in accordance with ICH Good Clinical Practice (GCP) and applicable country-specific regulatory requirements.

The study will be conducted in accordance with all applicable regulatory requirements, and with GSK policy.

The study will also be conducted in accordance with ICH Good Clinical Practice (GCP), all applicable subject privacy requirements, and the guiding principles of the current version of the Declaration of Helsinki. This includes, but is not limited to, the following:

IRB/IEC review and favorable opinion/approval of the study protocol and amendments as applicable

Obtaining signed informed consent

Investigator reporting requirements (e.g. reporting of AEs/SAEs/protocol deviations to IRB/IEC)

GSK will provide full details of the above procedures, either verbally, in writing, or both.

Signed informed consent must be obtained for each subject prior to participation in the study

The IEC/IRB, and where applicable the regulatory authority, approve the clinical protocol and all optional assessments, including genetic research.

Optional assessments (including those in a separate protocol and/or under separate informed consent) and the clinical protocol should be concurrently submitted for approval unless regulation requires separate submission.

Approval of the optional assessments may occur after approval is granted for the clinical protocol where required by regulatory authorities. In this situation, written approval of the clinical protocol should state that approval of optional assessments is being deferred and the study, with the exception of the optional assessments, can be initiated.

10.3. Quality Control (Study Monitoring)

In accordance with applicable regulations including GCP, and GSK procedures, GSK monitors will contact the site prior to the start of the study to review with the site staff the protocol, study requirements, and their responsibilities to satisfy regulatory, ethical, and GSK requirements.

When reviewing data collection procedures, the discussion will also include identification, agreement and documentation of data items for which the CRF will serve as the source document.

GSK will monitor the study and site activity to verify that the:

Data are authentic, accurate, and complete.

Safety and rights of subjects are being protected.

Study is conducted in accordance with the currently approved protocol and any other study agreements, GCP, and all applicable regulatory requirements.

The investigator and the head of the medical institution (where applicable) agrees to allow the monitor direct access to all relevant documents.

10.4. Quality Assurance

To ensure compliance with GCP and all applicable regulatory requirements, GSK may conduct a quality assurance assessment and/or audit of the site records, and the regulatory agencies may conduct a regulatory inspection at any time during or after completion of the study.

In the event of an assessment, audit or inspection, the investigator (and institution) must agree to grant the advisor(s), auditor(s) and inspector(s) direct access to all relevant documents and to allocate their time and the time of their staff to discuss the conduct of the study, any findings/relevant issues and to implement any corrective and/or preventative actions to address any findings/issues identified.

10.5. Study and Site Closure

Upon completion or premature discontinuation of the study, the GSK monitor will conduct site closure activities with the investigator or site staff, as appropriate, in accordance with applicable regulations including GCP, and GSK Standard Operating Procedures.

GSK reserves the right to temporarily suspend or prematurely discontinue this study at any time for reasons including, but not limited to, safety or ethical issues or severe non-compliance. For multicenter studies, this can occur at one or more or at all sites.

If GSK determines such action is needed, GSK will discuss the reasons for taking such action with the investigator or the head of the medical institution (where applicable). When feasible, GSK will provide advance notification to the investigator or the head of the medical institution, where applicable, of the impending action.

If the study is suspended or prematurely discontinued for safety reasons, GSK will promptly inform all investigators, heads of the medical institutions (where applicable) and/or institution(s) conducting the study. GSK will also promptly inform the relevant regulatory authorities of the suspension or premature discontinuation of the study and the reason(s) for the action.

If required by applicable regulations, the investigator or the head of the medical institution (where applicable) must inform the IRB/IEC promptly and provide the reason for the suspension or premature discontinuation.

10.6. Records Retention

Following closure of the study, the investigator or the head of the medical institution (where applicable) must maintain all site study records (except for those required by local regulations to be maintained elsewhere), in a safe and secure location.

The records must be maintained to allow easy and timely retrieval, when needed (e.g., for a GSK audit or regulatory inspection) and must be available for review in conjunction with assessment of the facility, supporting systems, and relevant site staff.

Where permitted by local laws/regulations or institutional policy, some or all of these records can be maintained in a format other than hard copy (e.g., microfiche, scanned, electronic); however, caution needs to be exercised before such action is taken.

The investigator must ensure that all reproductions are legible and are a true and accurate copy of the original and meet accessibility and retrieval standards, including re-generating a hard copy, if required. Furthermore, the investigator must ensure there is an acceptable back-up of these reproductions and that an acceptable quality control process exists for making these reproductions.

GSK will inform the investigator of the time period for retaining these records to comply with all applicable regulatory requirements. The minimum retention time will meet the strictest standard applicable to that site for the study, as dictated by any institutional requirements or local laws or regulations, GSK standards/procedures, and/or institutional requirements.

The investigator must notify GSK of any changes in the archival arrangements, including, but not limited to, archival at an off-site facility or transfer of ownership of the records in the event the investigator is no longer associated with the site.

10.7. Provision of Study Results to Investigators, Posting of Information on Publically Available Clinical Trials Registers and Publication

Where required by applicable regulatory requirements, an investigator signatory will be identified for the approval of the clinical study report. The investigator will be provided reasonable access to statistical tables, figures, and relevant reports and will have the opportunity to review the complete study results at a GSK site or other mutually-agreeable location.

GSK will also provide the investigator with the full summary of the study results. The investigator is encouraged to share the summary results with the study subjects, as appropriate.

GSK will provide the investigator with the randomization codes for their site only after completion of the full statistical analysis.

The procedures and timing for public disclosure of the results summary and for development of a manuscript for publication will be in accordance with GSK Policy.

11. REFERENCES

An Official ATS Clinical Practice Guideline: Interpretation of Exhaled Nitric Oxide Levels (FeNO) for Clinical Applications. Am J Respir Crit Care Med. 2011; 184: 602−615

ATS/ERS Recommendations for Standardized Procedures for the Online and Offline Measurement of Exhaled Lower Respiratory Nitric Oxide and Nasal Nitric Oxide. Am J Respir Crit Care Med. 2005; 171: 912–930

GlaxoSmithKline Document Number 2010N110564_04. GSK2245035 Investigator's Brochure. December 2015.

GlaxoSmithKline Document Number 2011N120740_00. A randomized, single-blind, placebo-controlled first time into human study to investigate the safety, tolerability, pharmacokinetics and pharmacodynamics of intranasal dosing with GSK2245035, a TLR7 agonist, in healthy volunteers and allergic rhinitics. July 2012.

GlaxoSmithKline Document Number 2011N126275_00. A randomised, double-blind, placebo-controlled study to investigate the safety, pharmacodynamics and efficacy against allergic reactivity of repeat intranasal administration of the TLR7 agonist GSK2245035 in subjects with respiratory allergies. January 2012.

GlaxoSmithKline Document Number 2013N174200_00. A randomized, double blind, placebo-controlled study to investigate the safety, pharmacodynamics and effect on allergic reactivity of repeat intranasal administration of the TLR7 agonist GSK2245035 in subjects with respiratory allergies. August 2015.

Global Initiative for Asthma (GINA). Global Strategy for Asthma management and prevention 2015. Available from www.ginasthma.org.

Hankinson J L, Odencrantz, JR; Feden, KB. Spirometric Reference Values from a Sample of the General U.S. Population. *AM J Respir Crit care Med.* 1999;159:179-187.

Hatcher RA, Trussell J, Nelson AL, Cates W Jr, Stewart F, Kowal D, editors. Contraceptive Technology. 19th edition. New York: Ardent Media, 2007(a): 24. Table 3-2.

Leaker B, Singh D, Lindgren S, et al., editors. The Effects Of The Novel Toll-Like Receptor 7 (TLR7) Agonist AZD8848 On Allergen-Induced Responses In Patients With Mild Asthma. American Thoracic Society International Conference; 2012; San Francisco, California, USA.

Quanjer PH, Stanojevic S, Cole TJ, Baur X, Hall GL, Culver BH, Enright PL, Hankinson JL, Ip MS, Zheng J, Stocks J; ERS Global Lung Function Initiative. Multi-ethnic reference values for spirometry for the 3-95-yr age range: the global lung function 2012 equations. Eur Respir J. 2012;40:1324-43.

12. APPENDICES

12.1. Appendix 1 – Abbreviations and Trademarks

Abbreviations

AC	Allergen challenge
AE	Adverse Event
ALT	Alanine aminotransferase (SGPT)
ANOVA	Analysis of Variance
AR	Allergic Rhinitis or Allergic Rhinitics
AST	Aspartate aminotransferase (SGOT)
AUC	Area under concentration-time curve
AUC(0-∞)	Area under the concentration-time curve from time zero (pre-dose) extrapolated to infinite time
%AUCex	Percentage of AUC(0-∞) obtained by extrapolation
AUC(0-x)	Area under the concentration-time curve from zero (pre-dose) to some fixed nominal time x
AUC(0-t)	Area under the concentration-time curve from time zero (pre-dose) to last time of quantifiable concentration within a subject across all treatments
AUC(0-τ)	Area under the concentration-time curve over the dosing interval
AZ	AstraZeneca
β-HCG	Beta-Human Chorionic Gonadotropin
BAC	Bronchial allergen challenge
BMI	Body mass index
BUN	Blood urea nitrogen
CI	Confidence Interval
CIB	Clinical Investigator's Brochure
CRF	Case Report Form
CRO	Contract Research Organization
CRS	Cytokine Release Syndrome
CV	Coefficient of variance
DMPK	Drug Metabolism and Pharmacokinetics
DNA	Deoxyribonucleic acid
DRE	Disease Related Event
DV	Dosing Visit
EAR	Early asthmatic response
ECG	Electrocardiogram
eNO	Exhaled Nitric Oxide
FDA	Food and Drug Administration
FEV_1	Forced expiratory volume in 1 second
FSH	Follicle Stimulating Hormone
FU	Follow-up
GCP	Good Clinical Practice
GCSP	Global Clinical Safety and Pharmacovigilence

GLP	Good Laboratory Practice
GSK	GlaxoSmithKline
HBsAg	Hepatitis B surface antigen
hCG	Human chorionic gonadotropin
HIV	Human Immunodeficiency Virus
h/hr	Hour(s)
HR	Heart rate
HRT	Hormone replacement therapy
HVT	Healthy volunteers
IB	Investigator's Brochure
IC	Intradermal challenge
ICH	International Conference on Harmonization of Technical Requirements for Registration of Pharmaceuticals for Human Use
ICS	Inhaled corticosteroids
IEC	Independent Ethics Committee
IFN	Interferon
Ig	Immunoglobulin
I.N.	Intranasal
IND	Investigational New Drug
INR	International normalized ratio
IP	Investigational Product
IP-10	Interferon inducible protein 10
IRB	Institutional Review Board
IRT	Interactive Response Technology
IU	International Unit
IV	Intravenous
Kg	Kilogram
L	Liter
LABA	Long-acting $beta_2$-agonist
LAR	Late asthmatic response
µg	Microgram
µL	Microliter
MCHC	Mean corpuscular hemoglobin concentration
MCV	Mean corpuscular volume
MedDRA	Medical Dictionary for Regulatory Activities
Mg	Milligrams
mL	Milliliter
mm	Millimeters
MSDS	Material Safety Data Sheet
msec	Milliseconds
NAC	Nasal allergen challenge
ng	nanogram
NO	Nitric Oxide
NOAEL	No observed adverse event level
OD	Once a day, once daily
PAX	Paired box gene

PBMC	Peripheral blood mononuclear cell
PBS	Phosphate buffered saline
PD	Pharmacodynamic
PEF	Peak Expiratory Flow
PK	Pharmacokinetic
PNIF	Peak Nasal Inspiratory Flow
PTS	Platform Technology Sciences
QC	Quality control
QTcB	QT duration corrected for heart rate by Bazett’s formula
QTcF	QT duration corrected for heart rate by Fridericia’s formula
RAP	Reporting and Analysis Plan
RBC	Red blood cells
RNA	Ribonucleic acid
SABA	Short-acting beta$_2$-agonist
SAE	Serious adverse event(s)
SAR	Seasonal allergic rhinitics
SAS	Statistical Analysis Software
SD	Standard deviation
SOP	Standard Operating Procedure
SRM	Study Reference Manual
T	Time of last observed quantifiable concentration
t½	Terminal phase half-life
Th	T helper cell
Tmax	Time of occurrence of Cmax
TNSS	Total nasal symptoms score
TLR7	Toll like receptor 7
Treg	Regulated T cells
ULN	Upper limit of normal
UK	United Kingdom
US	United States
WBC	White blood cells

Trademark Information

Trademarks of the GlaxoSmithKline group of companies
NONE

Trademarks not owned by the GlaxoSmithKline group of companies
None

12.2. Appendix 2: Liver Safety Required Actions and Follow up Assessments

Phase II Liver Chemistry Stopping and Increased Monitoring Algorithm

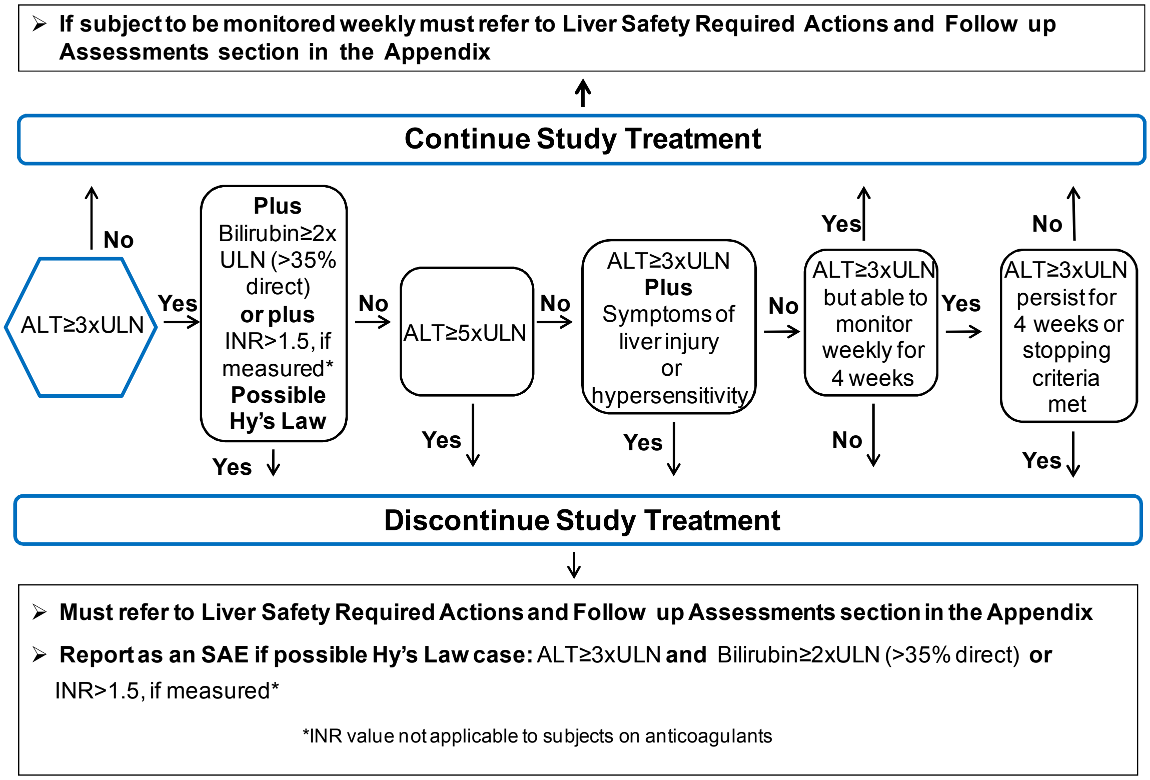

Phase II liver chemistry increased monitoring criteria with continued therapy

Liver Chemistry Increased Monitoring Criteria – Liver Monitoring Event	
**Criteria**	**Actions**
ALT ≥3xULN and <5xULN **and** bilirubin <2xULN, **without** symptoms believed to be related to liver injury or hypersensitivity, **and** who can be monitored weekly for 4 weeks	• Notify the GSK medical monitor **within 24 hours** of learning of the abnormality to discuss subject safety.<br>• Subject can continue study treatment<br>• Subject must return weekly for repeat liver chemistries (ALT, AST, alkaline phosphatase, bilirubin) until they resolve, stabilise or return to within baseline<br>• If at any time subject meets the liver chemistry stopping criteria, proceed as described above<br>• If, after 4 weeks of monitoring, ALT <3xULN and bilirubin <2xULN, monitor subjects twice monthly until liver chemistries normalize or return to within baseline.

References

James LP, Letzig L, Simpson PM, Capparelli E, Roberts DW, Hinson JA, Davern TJ, Lee WM. Pharmacokinetics of Acetaminophen-Adduct in Adults with Acetaminophen Overdose and Acute Liver Failure. Drug Metab Dispos 2009; 37:1779-1784.

12.3. Appendix 3 - Genetic Research

Genetics – Background

Naturally occurring genetic variation may contribute to inter-individual variability in response to medicines, as well as an individual's risk of developing specific diseases. Genetic factors associated with disease characteristics may also be associated with response to therapy, and could help to explain some clinical study outcomes. For example, genetic variants associated with age-related macular degeneration (AMD) are reported to account for much of the risk for the condition [Gorin, 2012] with certain variants reported to influence treatment response [Chen, 2012]. Thus, knowledge of the genetic etiology of disease may better inform understanding of disease and the development of medicines. Additionally, genetic variability may impact the pharmacokinetics (absorption, distribution, metabolism, and elimination), or pharmacodynamics (relationship between concentration and pharmacologic effects or the time course of pharmacologic effects) of a specific medicine and/or clinical outcomes (efficacy and/or safety) observed in a clinical study.

Genetic Research Objectives and Analyses

The objectives of the genetic research are to investigate the relationship between genetic variants and:

Response to medicine, including any treatment regimens under investigation in this study or any concomitant medicines;

Asthma susceptibility, severity, and progression and related conditions

Genetic data may be generated while the study is underway or following completion of the study. Genetic evaluations may include focused candidate gene approaches and/or examination of a large number of genetic variants throughout the genome (whole genome analyses). Genetic analyses will utilize data collected in the study and will be limited to understanding the objectives highlighted above. Analyses may be performed using data from multiple clinical studies to investigate these research objectives.

Appropriate descriptive and/or statistical analysis methods will be used. A detailed description of any planned analyses will be documented in a Reporting and Analysis Plan (RAP) prior to initiation of the analysis. Planned analyses and results of genetic investigations will be reported either as part of the clinical RAP and study report, or in a separate genetics RAP and report, as appropriate.

Study Population

Any subject who is enrolled in the study can participate in genetic research. Any subject who has received an allogeneic bone marrow transplant must be excluded from the genetic research.

Study Assessments and Procedures

A key component of successful genetic research is the collection of samples during clinical studies. Collection of samples, even when no *a priori* hypothesis has been identified, may enable future genetic analyses to be conducted to help understand variability in disease and medicine response.

A 6 ml blood sample will be taken for Deoxyribonucleic acid (DNA) extraction. A Blood sample is collected at the baseline visit, after the subject has been randomized and provided informed consent for genetic research. Instructions for collection and shipping of the genetic sample are described in the laboratory manual. The DNA from the blood sample may undergo quality control analyses to confirm the integrity of the sample. If there are concerns regarding the quality of the sample, then the sample may be destroyed. The blood sample is taken on a single occasion unless a duplicate sample is required due to an inability to utilize the original sample.

The genetic sample is labelled (or "coded") with the same study specific number used to label other samples and data in the study. This number can be traced or linked back to the subject by the investigator or site staff. Coded samples do not carry personal identifiers (such as name or social security number).

Samples will be stored securely and may be kept for up to 15 years after the last subject completes the study, or GSK may destroy the samples sooner. GSK or those working with GSK (for example, other researchers) will only use samples collected from the study for the purpose stated in this protocol and in the informed consent form. Samples may be used as part of the development of a companion diagnostic to support the GSK medicinal product.

Subjects can request their sample to be destroyed at any time.

Informed Consent

Subjects who do not wish to participate in the genetic research may still participate in the study. Genetic informed consent must be obtained prior to any blood being taken.

Subject Withdrawal from Study

If a subject who has consented to participate in genetic research withdraws from the clinical study for any reason other than being lost to follow-up, the subject will be given a choice of one of the following options concerning the genetic sample, if already collected:

Continue to participate in the genetic research in which case the genetic DNA sample is retained

Discontinue participation in the genetic research and destroy the genetic DNA sample

If a subject withdraws consent for genetic research or requests sample destruction for any reason, the investigator must complete the appropriate documentation to request sample

destruction within the timeframe specified by GSK and maintain the documentation in the site study records.

Genotype data may be generated during the study or after completion of the study and may be analyzed during the study or stored for future analysis.

If a subject withdraws consent for genetic research and genotype data has not been analyzed, it will not be analyzed or used for future research.

Genetic data that has been analyzed at the time of withdrawn consent will continue to be stored and used, as appropriate.

Screen Failures

If a sample for genetic research has been collected and it is determined that the subject does not meet the entry criteria for participation in the study, then the investigator should instruct the subject that their genetic sample will be destroyed. No forms are required to complete this process as it will be completed as part of the consent and sample reconciliation process. In this instance a sample destruction form will not be available to include in the site files.

Provision of Study Results and Confidentiality of Subject’s Genetic Data

GSK may summarize the genetic research results in the clinical study report, or separately and may publish the results in scientific journals.

GSK may share genetic research data with other scientists to further scientific understanding in alignment with the informed consent. GSK does not inform the subject, family members, insurers, or employers of individual genotyping results that are not known to be relevant to the subject’s medical care at the time of the study, unless required by law. This is due to the fact that the information generated from genetic studies is generally preliminary in nature, and therefore the significance and scientific validity of the results are undetermined. Further, data generated in a research laboratory may not meet regulatory requirements for inclusion in clinical care.

References

Chen H, Yu KD, Xu GZ. Association between Variant Y402H in Age-Related Macular Degeneration (AMD) Susceptibility Gene CFH and Treatment Response of AMD: A Meta-Analysis. PloS ONE 2012; 7: e42464

Gorin MB. Genetic insights into age-related macular degeneration: Controversies addressing risk, causality, and therapeutics. Mol. Asp. Med. 2012; 33: 467-486.

12.4. Appendix 4: Definition of and Procedures for Recording, Evaluating, Follow-Up and Reporting of Adverse Events

12.4.1. Definition of Adverse Events

**Adverse Event Definition:**
An AE is any untoward medical occurrence in a patient or clinical investigation subject, temporally associated with the use of a medicinal product, whether or not considered related to the medicinal product.<br>NOTE: An AE can therefore be any unfavorable and unintended sign (including an abnormal laboratory finding), symptom, or disease (new or exacerbated) temporally associated with the use of a medicinal product.

**Events meeting AE definition include:**
Any abnormal laboratory test results (hematology, clinical chemistry, or urinalysis) or other safety assessments (e.g., ECGs, radiological scans, vital signs measurements), including those that worsen from baseline, and felt to be clinically significant in the medical and scientific judgement of the investigator.<br>Exacerbation of a chronic or intermittent pre-existing condition including either an increase in frequency and/or intensity of the condition.<br>New conditions detected or diagnosed after study treatment administration even though it may have been present prior to the start of the study.<br>Signs, symptoms, or the clinical sequelae of a suspected interaction.<br>Signs, symptoms, or the clinical sequelae of a suspected overdose of either study treatment or a concomitant medication (overdose per se will not be reported as an AE/SAE unless this is an intentional overdose taken with possible suicidal/self-harming intent. This should be reported regardless of sequelae).<br>"Lack of efficacy" or "failure of expected pharmacological action" per se will not be reported as an AE or SAE. However, the signs and symptoms and/or clinical sequelae resulting from lack of efficacy will be reported if they fulfil the definition of an AE or SAE.

**Events <u>NOT</u> meeting definition of an AE include:**
Any clinically significant abnormal laboratory findings or other abnormal safety assessments which are associated with the underlying disease, unless judged by the investigator to be more severe than expected for the subject's condition.<br>The disease/disorder being studied or expected progression, signs, or symptoms of the disease/disorder being studied, unless more severe than expected for the subject's condition.<br>Medical or surgical procedure (e.g., endoscopy, appendectomy): the condition that leads to the procedure is an AE.<br>Situations where an untoward medical occurrence did not occur (social and/or convenience admission to a hospital).<br>Anticipated day-to-day fluctuations of pre-existing disease(s) or condition(s) present or detected at the start of the study that do not worsen.

12.4.2. Definition and Grading of CRS-related Adverse Events

In the study, symptoms potentially related to cytokine induction (headache, chills/rigors, fever, nausea, vomiting, diarrhoea, arthralgia, myalgia, hypotension) occurring within 24 hours of dosing will be individually reported as AEs and graded as described below:

	Severity Grade				
Symptom/Sign	0<br>None	1<br>Mild	2<br>Moderate	3<br>Severe	4<br>Disabling/Life-threatening
Headache	None	Mild pain not interfering with function	Moderate pain: pain or analgesics interfering with function, but not interfering with activities of daily living	Severe pain: pain or analgesics severely interfering with activities of daily living	Disabling
Fever	None	38.0 - 39.0°C (100.4 - 102.2°F); Grade 1 after antipyretics or resolves	39.1 - 40.0°C (102.3 - 104.0°F); remains Grade 2 with or without antipyretics	> 40.0°C (>104.0°F) for < 24hrs despite antipyretics	> 40.0°C (>104.0°F) for > 24hrs despite antipyretics
Chills/Rigors	None	Mild, transient, requiring no or symptomatic treatment (e.g., blanket); remains at Grade 1 or resolves after non-narcotic medication	Moderate, requiring non-narcotic medication; lasts several hours before resolution	Severe and/or prolonged, requiring narcotic medication	Not responsive to narcotic medication
Nausea	None	Mild, but able to eat	Mild but oral intake significantly decreased	Moderate; no significant intake, requiring IV fluids; <24 hrs duration	Severe; prolonged, requiring IV fluids; ≥ 24 hrs duration
Vomiting	None	1 episode in 24 hours; antiemetics resolve or keep at Grade 1	2-5 episodes in 24 hours despite antiemetics	≥6 episodes in 24 hours; despite antiemetics or need for IV fluids	Requiring parenteral nutrition; or physiologic consequences requiring intensive care; hemodynamic compromise
Diarrhea	None	Increase of < 4 stools/day over pre-treatment	Increase of 4-6 stools/day, or nocturnal stools	Increase of ≥7 stools/day or incontinence; or need for parenteral support for dehydration	Physiologic consequences requiring intensive care; or hemodynamic compromise

Cytokine Release Syndrome (CRS) AE grading (continued)

	Severity Grade				
Symptom/Sign	0<br>None	1<br>Mild	2<br>Moderate	3<br>Severe	4<br>Disabling/Life-threatening
Arthralgia (joint pain)	None	Mild pain not interfering with function	Moderate pain: pain or analgesics interfering with function, but not interfering with activities of daily living	Severe pain: pain or analgesics severely interfering with activities of daily living	Disabling
Myalgia (muscle pain)	None	Mild pain not interfering with function	Moderate pain: pain or analgesics interfering with function, but not interfering with activities of daily living	Severe pain: pain or analgesics severely interfering with activities of daily living	Disabling
Hypotension	None	BP changes, but not requiring therapy (including transient orthostatic hypotension)	Requiring brief fluid replacement or other therapy but not hospitalization; no physiologic consequences	Requiring therapy and sustained medical attention, but resolves without persisting physiologic consequences	Shock (associated with acidemia; impairing vital organ function due to tissue hypoperfusion)

12.4.3. Definition of Serious Adverse Events

If an event is not an AE per definition above, then it cannot be an SAE even if serious conditions are met (e.g., hospitalization for signs/symptoms of the disease under study, death due to progression of disease, etc).

**Serious Adverse Event (SAE) is defined as any untoward medical occurrence that, at any dose:**
**a. Results in death**
**b. Is life-threatening**<br>NOTE:<br>The term 'life-threatening' in the definition of 'serious' refers to an event in which the subject was at risk of death at the time of the event. It does not refer to an event, which hypothetically might have caused death, if it were more severe.
**c. Requires hospitalization or prolongation of existing hospitalization**<br>NOTE:<br>In general, hospitalization signifies that the subject has been detained (usually involving at least an overnight stay) at the hospital or emergency ward for observation and/or treatment that would not have been appropriate in the physician's office or out-patient

setting. Complications that occur during hospitalization are AEs. If a complication prolongs hospitalization or fulfills any other serious criteria, the event is serious. When in doubt as to whether “hospitalization” occurred or was necessary, the AE should be considered serious.

Hospitalization for elective treatment of a pre-existing condition that did not worsen from baseline is not considered an AE.

d. Results in disability/incapacity

NOTE:

The term disability means a substantial disruption of a person’s ability to conduct normal life functions.

This definition is not intended to include experiences of relatively minor medical significance such as uncomplicated headache, nausea, vomiting, diarrhea, influenza, and accidental trauma (e.g. sprained ankle) which may interfere or prevent everyday life functions but do not constitute a substantial disruption

e. Is a congenital anomaly/birth defect

f. Other situations:

Medical or scientific judgment should be exercised in deciding whether reporting is appropriate in other situations, such as important medical events that may not be immediately life-threatening or result in death or hospitalization but may jeopardize the subject or may require medical or surgical intervention to prevent one of the other outcomes listed in the above definition. These should also be considered serious.

Examples of such events are invasive or malignant cancers, intensive treatment in an emergency room or at home for allergic bronchospasm, blood dyscrasias or convulsions that do not result in hospitalization, or development of drug dependency or drug abuse

g. Is associated with liver injury and impaired liver function defined as:

ALT ≥ 3xULN and total bilirubin* ≥ 2xULN (>35% direct), **or**

ALT ≥ 3xULN and International Normalised Ratio (INR)** > 1.5.

* Serum bilirubin fractionation should be performed if testing is available; if unavailable, measure urinary bilirubin via dipstick. If fractionation is unavailable and ALT ≥ 3xULN and total bilirubin ≥ 2xULN, then the event is still to be reported as an SAE.

** INR testing not required per protocol and the threshold value does not apply to subjects receiving anticoagulants. If INR measurement is obtained, the value is to be recorded on the SAE form.

Refer to Appendix 2 for the required liver chemistry follow-up instructions

12.4.4. Definition of Cardiovascular Events

**Cardiovascular Events (CV) Definition:**
Investigators will be required to fill out the specific CV event page of the CRF for the following AEs and SAEs:
Myocardial infarction/unstable angina
Congestive heart failure
Arrhythmias
Valvulopathy
Pulmonary hypertension
Cerebrovascular events/stroke and transient ischemic attack
Peripheral arterial thromboembolism
Deep venous thrombosis/pulmonary embolism
Revascularization

12.4.5. Recording of AEs and SAEs

**AEs and SAE Recording:**
When an AE/SAE occurs, it is the responsibility of the investigator to review all documentation (e.g., hospital progress notes, laboratory, and diagnostics reports) relative to the event.
The investigator will then record all relevant information regarding an AE/SAE in the CRF
It is **not** acceptable for the investigator to send photocopies of the subject's medical records to GSK in lieu of completion of the GSK, AE/SAE CRF page.
There may be instances when copies of medical records for certain cases are requested by GSK. In this instance, all subject identifiers, with the exception of the subject number, will be blinded on the copies of the medical records prior to submission of to GSK.
The investigator will attempt to establish a diagnosis of the event based on signs, symptoms, and/or other clinical information. In such cases, the diagnosis will be documented as the AE/SAE and not the individual signs/symptoms.

12.4.6. Evaluating AEs and SAEs

Assessment of Intensity

The investigator will make an assessment of intensity for each AE and SAE reported during the study and will assign it to one of the following categories:

Mild: An event that is easily tolerated by the subject, causing minimal discomfort and not interfering with everyday activities.

Moderate: An event that is sufficiently discomforting to interfere with normal everyday activities

Severe: An event that prevents normal everyday activities. - an AE that is assessed as severe will not be confused with an SAE. Severity is a category utilized for rating the intensity of an event; and both AEs and SAEs can be assessed as severe.

An event is defined as 'serious' when it meets at least one of the pre-defined outcomes as described in the definition of an SAE.

Assessment of Causality

The investigator is obligated to assess the relationship between study treatment and the occurrence of each AE/SAE.

A "reasonable possibility" is meant to convey that there are facts/evidence or arguments to suggest a causal relationship, rather than a relationship cannot be ruled out.

The investigator will use clinical judgment to determine the relationship.

Alternative causes, such as natural history of the underlying diseases, concomitant therapy, other risk factors, and the temporal relationship of the event to the study treatment will be considered and investigated.

The investigator will also consult the Investigator Brochure (IB) and/or Product Information, for marketed products, in the determination of his/her assessment.

For each AE/SAE the investigator **must** document in the medical notes that he/she has reviewed the AE/SAE and has provided an assessment of causality.

There may be situations when an SAE has occurred and the investigator has minimal information to include in the initial report to GSK. However, **it is very important that the investigator always make an assessment of causality for every event prior to the initial transmission of the SAE data to GSK.**

The investigator may change his/her opinion of causality in light of follow-up information, amending the SAE data collection tool accordingly.

The causality assessment is one of the criteria used when determining regulatory reporting requirements.

Follow-up of AEs and SAEs

The investigator is obligated to perform or arrange for the conduct of supplemental measurements and/or evaluations as may be indicated or as requested by GSK to elucidate as fully as possible the nature and/or causality of the AE or SAE.

The investigator is obligated to assist. This may include additional laboratory tests or investigations, histopathological examinations or consultation with other health care professionals.

If a subject dies during participation in the study or during a recognized follow-up period, the investigator will provide GSK with a copy of any post-mortem findings, including histopathology.

New or updated information will be recorded in the originally completed CRF.

The investigator will submit any updated SAE data to GSK within the designated reporting time frames.

12.4.7. Reporting of SAEs to GSK

SAE reporting to GSK via electronic data collection tool

Primary mechanism for reporting SAEs to GSK will be the electronic data collection tool

If the electronic system is unavailable for greater than 24 hours, the site will use the paper SAE data collection tool and fax it to the GSK Medical Monitor and GSK Study Manager

Site will enter the serious adverse event data into the electronic system as soon as it becomes available.

The investigator will be required to confirm review of the SAE causality by ticking the ‘reviewed’ box at the bottom of the eCRF page within 72 hours of submission of the SAE.

After the study is completed at a given site, the electronic data collection tool (e.g., InForm system) will be taken off-line to prevent the entry of new data or changes to existing data

If a site receives a report of a new SAE from a study subject or receives updated data on a previously reported SAE after the electronic data collection tool has been taken off-line, the site can report this information on a paper SAE form or to the GSK Medical Monitor and GSK Study Manager by telephone.

Contacts for SAE receipt can be found at the beginning of this protocol on the Sponsor/Medical Monitor Contact Information page.

12.5. Appendix 5: Modified List of Highly Effective Methods for Avoiding Pregnancy and Collection of Pregnancy Information

12.5.1. Modified List of Highly Effective Methods for Avoiding Pregnancy

Contraceptive requirements for male subjects with female partners of reproductive potential (when applicable).

Male subjects with female partners of child bearing potential must comply with the following contraception requirements from the time of first dose of study medication until [at least five half-lives of study medication OR for a cycle of spermatogenesis following five terminal half-lives] after the last dose of study medication.

1. Vasectomy with documentation of azoospermia. The documentation on male sterility can come from the site personnel's: review of subject's medical records, medical examination and/or semen analysis, or medical history interview.
2. Male condom plus partner use of one of the contraceptive options below that meets the Standard Operating Procedure (SOP) effectiveness criteria including a <1% rate of failure per year, as stated in the product label:
   - Contraceptive subdermal implant
   - Intrauterine device or intrauterine system
   - Combined estrogen and progestogen oral contraceptive [Hatcher, 2011]
   - Injectable progestogen [Hatcher, 2011]
   - Contraceptive vaginal ring [Hatcher, 2011]
   - Percutaneous contraceptive patches [Hatcher, 2011]

These allowed methods of contraception are only effective when used consistently, correctly and in accordance with the product label. The investigator is responsible for ensuring that subjects understand how to properly use these methods of contraception.

12.5.2. Collection of Pregnancy Information

Investigator will collect pregnancy information on any female subject, who becomes pregnant while participating in this study (this may also be a female partner of a male subject in the study)

Information will be recorded on the appropriate form and submitted to GSK within 2 weeks of learning of a subject's pregnancy.

Subject will be followed to determine the outcome of the pregnancy. The investigator will collect follow up information on mother and infant, which will be forwarded to GSK. Generally, follow-up will not be required for longer than 6 to 8 weeks beyond the estimated delivery date.

Any termination of pregnancy will be reported, regardless of fetal status (presence or absence of anomalies) or indication for procedure.

While pregnancy itself is not considered to be an AE or SAE, any pregnancy complication or elective termination of a pregnancy will be reported as an AE or SAE.

A spontaneous abortion is always considered to be an SAE and will be reported as such.

Any SAE occurring as a result of a post-study pregnancy which is considered reasonably related to the study treatment by the investigator, will be reported to GSK as described in Appendix 4. While the investigator is not obligated to actively seek this information in former study participants, he or she may learn of an SAE through spontaneous reporting.

Any female subject who becomes pregnant while participatingwill discontinue study medication and will be withdrawn from the study

Investigator will attempt to collect pregnancy information on any female partner of a male study subject who becomes pregnant while participating in this study. This applies only to subjects who are randomized to receive study medication.

After obtaining the necessary signed informed consent from the female partner directly, the investigator will record pregnancy information on the appropriate form and submit it to GSK within 2 weeks of learning of the partner's pregnancy

Partner will also be followed to determine the outcome of the pregnancy. Information on the status of the mother and child will be forwarded to GSK.

Generally, follow-up will be no longer than 6 to 8 weeks following the estimated delivery date. Any termination of the pregnancy will be reported regardless of fetal status (presence or absence of anomalies) or indication for procedure.

12.5.3. References

CHMP, 2005 - EMA - Guideline on Adjuvants in Vaccines for Human Use, (CHMP/VEG/134716/2004), January 2005.

Cole LA, Khanlian SA, Sutton JM, Davies S, Rayburn WF. Accuracy of home pregnancy tests at the time of missed menses. Am J Ob Gyn 2004(190):100-5.

EMA/CHMP/ICH/449035/2009: General principles to address virus and vector shedding. http://www.ema.europa.eu/docs/en_GB/document_library/Scientific_guideline/2009/09/WC500002680.pdf Accessed 28 Nov 2014.

EMEA/CHMP/GTWP/125459/2006: Guideline on the Nonclinical Studies Required Before First Clinical Use of Gene Therapy Medicinal Products [2008]

FDA CBER Guidance for industry, "Considerations for Developmental Toxicity Studies for Preventive and Therapeutic Vaccines for Infectious Disease Indications. U.S. FDA; Feb. 2006.

FDA Medical Devices Safety Alerts and Notices, "Blood human chorionic gonadotropin (hCG) assays: What laboratorians should know about false-positive results", http://www.fda.gov/MedicalDevices/Safety/AlertsandNotices/TipsandArticlesonDeviceSafety/ucm109390.htm, accessed 17 Nov 2014.

Hatcher RA, Trussell J, Nelson AL, Cates W Jr, Stewart F, Kowal D, Policar MS, editors. Contraceptive Technology. 20th edition. Atlanta, Georgia: Ardent Media, Inc., 2011: 50. Table 3-2.

International Conference on Harmonization of Technical Requirements for Registration of Pharmaceuticals For Human Use. ICH Harmonized Tripartite Guideline. Detection of Toxicity to Reproduction for Medicinal Products & Toxicity to Male Fertility. Parent Guideline dated 24 June 1993 (Addendum dated 9 November 2000 incorporated in November 2005. ICH S5 (R2)

International Conference on Harmonization of Technical Requirements for Registration of Pharmaceuticals For Human Use. ICH Harmonized Tripartite Guideline. Nonclinical Evaluation for Anticancer Pharmaceuticals. ICH S9. 2009.

International Conference on Harmonization of Technical Requirements for Registration of Pharmaceuticals For Human Use. ICH Harmonized Tripartite Guideline. Nonclinical Safety Studies for the Conduct of Human Clinical Trials and Marketing Authorization for Pharmaceuticals. ICH M3 (R2). 2009.

International Conference on Harmonization of Technical Requirements for Registration of Pharmaceuticals For Human Use. ICH Harmonized Tripartite Guideline. Preclinical Safety Evaluation of Biotechnology-Derived Pharmaceuticals. ICH S6 and Addendum ICH S6 (R1). 2011.

Kronenberg HM, Melmed S, Polonsky KS, Larsen PR, editors. Williams Textbook of Endocrinology, 11th edition. Philadelphia: Saunders, 2008.

Strauss JF, Barbieri RL, editors. Yen and Jaffe’s Reproductive Endocrinology. 5th edition, Philadelphia, Elsevier/Saunders, 2004.

U.S. Dept of Health and Human Services, FDA, Center For Biologics Evaluation and Research: Guidance for Human Somatic Cell Therapy and Gene Therapy [1998]

WHO, 2013 - WHO - Guidelines on the Nonclinical Evaluation of Vaccine Adjuvants and Adjuvanted Vaccines, 2013

World Health Organization. WHO/CONRAD Technical Consultation on Nonoxynol-9. World Health Organization; Department of Reproductive Health and Research; Geneva; 9-10 October 2001. Summary Report. 2001. WHO/RHR/03.8.

12.6. Appendix 6 - Country Specific Requirements

No country-specific requirements exist.

12.7. Appendix 7: Protocol Amendment Changes

AMENDMENT [2]

Where the Amendment Applies

This amendment applies across any site that will be used in the study.

Summary of Amendment Changes with Rationale

The protocol title and text have been amended to clarify that while the investigator and subjects are blinded the sponsor-is unblinded.

Details of the procedures required at early withdrawal visits have been added.

Details around the Safety Review Team have been added to include a consultant external to GSK.

The inclusion and exclusion criteria have been updated in Section 5.1 and Section 5.2. and clarification around prohibited medications has been provided in Section 6.11.2. to align with updated exclusion criteria (Section 5.2).

Administrative changes have been made to the T&E table (Section 7.1) and text to ensure consistency throughout.

List of Specific Changes

Title: Updated to reflect that the study is sponsor-unblind.

PREVIOUS TEXT

A randomised, double-blind placebo-controlled, parallel group, 8-week treatment study to investigate the safety, pharmacodynamics, and effect of the TLR7 agonist, GSK2245035, on the allergen-induced asthmatic response in subjects with mild allergic asthma.

REVISED TEXT

A randomised, double-blind (sponsor open) placebo-controlled, parallel group, 8-week treatment study to investigate the safety, pharmacodynamics, and effect of the TLR7 agonist, GSK2245035, on the allergen-induced asthmatic response in subjects with mild allergic asthma.

This update has been made where necessary throughout the protocol.

Objectives/Endpoints: Updated to clarify what changes in FeNO measurement are being assessed; abbreviations for bronchial allergen challenge and nasal allergen challenge included. Nasal allergen administration changed to nasal allergen challenge for consistency.

To evaluate the induction of TLR7-associated pharmacodynamic (PD) biomarkers and exhaled nitric oxide (FeNO) following treatment with i.n. GSK2245035	TLR7-induced blood PD biomarkers.<br>TLR7 induced gene expression changes in blood<br>TLR7-induced nasal fluid PD biomarkers<br>FeNO levels
To evaluate the effect of treatment with i.n. GSK2245035 compared to placebo on Bronchial Allergen Challenge associated allergic biomarkers	Allergic biomarkers in induced sputum<br>Allergic biomarkers in blood<br>FeNO levels
To evaluate the effect of treatment with i.n. GSK2245035 compared to placebo on Nasal Allergen Administration associated allergic biomarkers	Allergic biomarkers in nasal fluids<br>Cellular profile of nasal mucosa

REVISED TEXT

To evaluate the induction of TLR7-associated pharmacodynamic (PD) biomarkers and exhaled nitric oxide (FeNO) following treatment with i.n. GSK2245035	TLR7-induced blood PD biomarkers.<br>TLR7 induced gene expression changes in blood<br>TLR7-induced nasal fluid PD biomarkers<br>Change in FeNO levels during the treatment phase
To evaluate the effect of treatment with i.n. GSK2245035 compared to placebo on Bronchial Allergen Challenge (BAC) associated allergic biomarkers	Allergic biomarkers in induced sputum<br>Allergic biomarkers in blood<br>Change in FeNO levels following BAC
To evaluate the effect of treatment with i.n. GSK2245035 compared to placebo on Nasal Allergen Challenge(NAC) associated allergic biomarkers	Allergic biomarkers in nasal fluids<br>Cellular profile of nasal mucosa

Bronchial Allergen Challenge has been replaced by BAC throughout the protocol and Nasal Allergen Challenge/Administration by NAC throughout the protocol.

Section 4.3. Type and Number of Subjects, Final Paragraph updated

PREVIOUS TEXT

Accounting for replacements and interim analysis recommendations, the total number of subjects to be enrolled in this study is not expected to exceed 60

REVISED TEXT

Accounting for replacements and interim analysis recommendations, the total number of subjects to be randomised in this study is not expected to exceed 60

Inclusion Criteria 5 updated to clarify timepoint of assessment

PREVIOUS TEXT

Pre-bronchodilator FEV_1 > 70 % predicted normal.

REVISED TEXT

Pre-bronchodilator FEV_1 > 70 % predicted normal at Screening Visit 1

Section 5.2. Exclusion Criteria

Two additional exclusion criteria have been added for clarification:

- Use of long-acting antihistamines within 7 days' or short-acting antihistamines within 72 hours prior to the screening skin prick test.
- Patient known to be intolerant to salbutamol or albuterol

Section 5.4 Withdrawal/Stopping Criteria

PREVIOUS TEXT

Subjects withdrawn from the study while already exposed to IP will be asked to complete all safety assessments and the follow-up procedures. Relevant pages of the case report form (CRF) should be completed by the investigator.

REVISED TEXT

Subjects withdrawn from the study while already exposed to IP will be asked to complete all safety assessments as outlined in the Time and Events Table (Section 7.1.2). Relevant pages of the case report form (CRF) should be completed by the investigator.

Section 6.3 Blinding: An additional paragraph has been inserted between paragraphs 2 and 3.

PREVIOUS TEXT

This will be a double-blind study (investigators and subjects are blinded, and sponsor will be unblinded).

During the study, there will be an unblinded interim analysis. Some key GSK study team members may be unblinded as part of this analysis and review. Initially, only the study statistician(s) (and supporting programmers), project physician lead, and medical monitor

will be unblinded, and if further data reviews are required then details of who were un-blinded to what data and when will be included in the clinical study report.

If the GSK medical monitor deems it necessary to unblind data in order to enhance the evaluation of safety and tolerability this will be permitted but also documented and discussed in the clinical study report. The GSK medical monitor will also decide whether to un-blind the investigator(s)/site(s) at that time, or just the sponsor study staff. Details of who were un-blinded to what data and when would then be included in the clinical study report.

REVISED TEXT

During the study, there will be an unblinded interim analysis. Some key GSK study team members may be unblinded as part of this analysis and review. Initially, only the study statistician(s) (and supporting programmers), project physician lead, and medical monitor will be unblinded, and if further data reviews are required then details of who were un-blinded to what data and when will be included in the clinical study report.

On-going safety reviews will be conducted during the course of the study in an unblinded manner. Key study team members may be unblinded as part of this review. An external consultant, who is not a GSK employee, may also be unblinded to enable independent review of emerging safety data.

If the GSK medical monitor deems it necessary to unblind data in order to enhance the evaluation of safety and tolerability this will be permitted but also documented and discussed in the clinical study report. The GSK medical monitor will also decide whether to un-blind the investigator(s)/site(s) at that time, or just the sponsor study staff. Details of who were un-blinded to what data and when would then be included in the clinical study report.

Section 6.10.2. Caffeine, Alcohol, and Tobacco: A third bullet point has been added for clarification.

PREVIOUS TEXT

Subjects will abstain from ingesting excessive amounts (more than one cup) of caffeine- or xanthine-containing products (e.g. coffee, tea, cola drinks, chocolate) for 6 hours prior to each dosing.

During each visit in the clinic, subjects will abstain from alcohol for 24 hours prior to entry to the clinic until collection of all assessment is completed.

REVISED TEXT

Subjects will abstain from ingesting excessive amounts (more than one cup) of caffeine- or xanthine-containing products (e.g. coffee, tea, cola drinks, chocolate) for 6 hours prior to each dosing.

During each visit in the clinic, subjects will abstain from alcohol for 24 hours prior to entry to the clinic until collection of all assessment is completed.

Subjects will be reminded that all forms of smoking should be avoided during the course of the study

Section 6.11.2 Permitted Medications and Non-Drug Therapies: Additional wording has been added to clarify which medications are permitted.

PREVIOUS TEXT

Any concomitant medications not specifically permitted will be considered on a case by case basis by the investigator and GSK medical monitor.

Subjects are permitted to take inhaled short-acting β2-agonists (SABA) as rescue treatment, but must withhold them for at least 6 hours prior to study visits with BAC (screening and follow-up visits 1-3).

REVISED TEXT

Any concomitant medications not specifically permitted will be considered on a case by case basis by the investigator and GSK medical monitor.

Subjects are permitted to take inhaled short-acting β2-agonists (SABA) as rescue treatment, but must withhold them for at least 6 hours prior to any study visits with lung function assessment or BAC (screening and follow-up visits 1-3).

Medication to control hypertension is permitted, providing it is anticipated to remain stable from screening to follow up. Beta blockers are not permitted.

Section 6.11.2 Prohibited Medications and Non-Drug Therapies. Clarification around the time period for which drugs are not permitted is provided.

PREVIOUS TEXT

Subjects may not receive the following treatments within 4 weeks prior to screening:

- Intranasal steroids
- Inhaled corticosteroid (ICS) with or without a long acting beta-2-agonist (LABA)
- Non-ICS controller asthma medications (i.e. leukotriene modifier, methylxanthines)

Subjects are prohibited from taking antihistamines within 72 hours prior to the screening and follow-up bronchial allergen challenges.

Subjects are prohibited from taking systemic corticosteroids within 6 weeks prior to screening

REVISED TEXT

Subjects may not receive the following treatments from 4 weeks prior to screening until the final follow-up visit:

- Intranasal steroids
- Inhaled corticosteroid (ICS) with or without a long acting beta-2-agonist (LABA)

- Non-ICS controller asthma medications (i.e. leukotriene modifier, methylxanthines).

Use of long-acting antihistamines within 7 days' or short-acting antihistamines within 72 hours prior to BAC, NAC or ID challenge is prohibited.

Subjects are prohibited from taking systemic corticosteroids within 6 weeks prior to screening.

Section 7.1 Time and Events Tables: These have been updated to provide consistency and clarification for the sites:
PREVIOUS TEXT

Section 7.1.1. Time and Events Table – Screening Visit(s)

Procedure	Screening Visit 1 (Day -28 to Day -22)	Screening Visit 2 (Day -21 to Day -3)	24 hours after Screening Visit 2 (Day -20 to Day -2)	Notes
Informed consent signing	X			Includes genetics consent
Inclusion and exclusion criteria/eligibility review	X	X		
Demographics	X			
Medical history (including substance usage and family history of premature CV disease)	X			
Past and current medical conditions including cardiovascular medical history	X			
Concomitant medication review	X			
Full physical exam including height and weight	X			
Urine drug/alcohol screen	X			
Urine or serum pregnancy test (all females)	X			No women of childbearing potential are being enrolled. However this test is needed to confirm pregnancy status at screening.
Serum FSH and estradiol for post-menopausal females	X			
HIV, Hep B and Hep C screen	X			
Laboratory assessments (including liver chemistries, haematology, clinical chemistry and urinalysis)	X			
Screening Spirometry assessment	X			To confirm FEV_1 > 70% predicted
12-lead ECG	X			
Vital signs	X			
Skin prick test	X			To confirm eligibility
FeNO measurement	X		X	Collected prior to sputum induction
Saline and incremental BAC challenge plus associated spirometry		X		Including baseline FEV_1 assessment and repeat FEV_1

Procedure	Screening Visit 1 (Day -28 to Day -22)	Screening Visit 2 (Day -21 to Day -3)	24 hours after Screening Visit 2 (Day -20 to Day -2)	Notes
measurements				measurements up to 10 hours post BAC
Sputum induction and processing for BAC associated sample collection	X		X	Sputum induction pre-BAC and 24 hours after BAC: (must be separated by at least 3 days).
Blood sample for assessment of allergic biomarkers in blood		X[1,2]	X[2]	[1] Collect blood samples for PBMC preparations (pre-BAC and 6 hours after BAC)<br>[2] Collect blood sample for haematology (WBC count with differential) pre-BAC and 24 hours after BAC
Provide diary card, PEF meters, rescue medication and thermometers to subjects		X		Only given out to subjects who are eligible following the BAC. Thermometers are to be given to record temperature as part of capturing AE information.
Nasal examination		X		
NAC)			X	Commence after the 24-hour BAC sputum and blood biomarker samples have been taken ..<br>Only to be done once subjects have completed a successful BAC.
Collect sample for nasal baiomarker assessment			X	Collect samples pre-NAC, 5 minutes and at 6 hours after NAC using nasal filters..
Nasal lavage			X	Collect nasal lavage pre-NAC, 5 minutes and at least 6 hours after NAC.<br>Collect nasal mucosa sample at least 6 hours after NAC.
ID allergy testing			X	Intradermal challenge to be administered after NAC. Measurements to be made 15 minutes and 6 hours post intradermal challenge

Section 7.1.2. Time and Events Table – Dosing visit (DV) 1 (Day 1) – DV8 (Week 8)

Procedure	Pre-dose	Dose	Post-dose				Notes
		0 hour	20 mins	1 hour	12 hours	24 hours	
Outpatient visit	X					X	Subjects get discharged 1-2 hours after dosing.<br>For DV1, 4, and 8, subjects will return to the unit 24 hours after dosing.
Inclusion and exclusion criteria/eligibility review	X						Only on DV1
12-lead ECG	X			X		X	Perform on DV1 and DV8 only
Vital signs (including temperature)	X[1]			X[1]		X[2]	[1] Perform on DV1-8<br>[2] Perform on DV1, 4 and DV8 only
Laboratory assessments (include liver chemistries, haematology, clinical chemistry and urinalysis)	X					X	Collect samples for laboratory assessments on DV1 and DV8
Blood sample for PK analysis	X		X	X			Collect samples on DV1, DV4 and DV8
Nasal examination	X					X	Conducted pre-dose and 24 hours post-dose on DV1 and DV8
Blood sample for biomarker analysis	X					X	Collect blood for serum samples and PBMC preparations pre-dose and 24 hrs post-dose on DV1 and DV8
Nasal lavage	X					X	Collect nasal lavage samples pre-dose and 24hrs post-dose on DV1, DV4 and DV8
Blood PAX collection	X[(3)]					X[(4)]	PAX tubes for TLR7/AFFY chip gene expression in blood collected pre-dose on DV1[(3)] and 24 hrs after DV8[(4)]
Randomisation via IRT		X					Randomisation is on DV1 only
Study drug administration		X					All doses are administered in the unit.
Dispensing of study medication		X					One device will be assigned on DV1. The second device will be assigned on DV5. All devices remain in the unit.
AE/SAE review	X						
Concomitant medication review	X						
TNSS score (diary card) review	X						Captured daily AM and PM from DV1 to FUV2
PEF (diary card) review	X						Captured daily AM and PM from DV1 to FUV2
FeNO measurement	X						Perform FeNO measurements pre-dose on all dosing visits 1-8
Sputum induction and processing for BAC associated						X	Sputum sample is conditional on having obtained a sputum sample from the subject at screening. Perform sputum induction after

Procedure	Pre-dose	Dose	Post-dose				Notes
		0 hour	20 mins	1 hour	12 hours	24 hours	
sample collection							collection of 24 hour post-dose DV8 blood and nasal samples (as pre BAC sample for FUV1).
Genetic sample	X						Take one sample on any of the Dosing Visits after genetics consent.
Diary card review	X						Review diary card at every dosing visit
Follow up phone call					X[5]	X[6]	[5] Following at least the first two doses of study treatment to assess tolerability<br>[6] On DV 2, 3, and 5-7, a follow-up phone call will be made approximately 24 hours post-dose to check if any CRS events have occurred, and if they have, that they have been accurately recorded in the diary card and they will then be transcribed into the eCRF.<br>For DV1, 4 and 8, subjects will be in the unit 24 hours after dosing, and any CRS events can be assessed and recorded directly by the site staff.

Section 7.1.3. Time and Events Table – Follow up visits (FUV1 to 3) (Weeks 9 to 20)

Procedure	Follow up Visit 1 (FUV1)		Follow-up Visit 2 (FUV2)			2 month follow-up phone call (Week 16)	Follow up Visit 3 (FUV3)			Notes
	Week 9 (1 week after DV8)	24 hours later	Week 11 (3 weeks after DV8)	Week 12 (4 weeks after DV8)	24 hours later		Week 19 (11 weeks after DV8)	Week 20 (12 weeks after DV8)	24 hours later	
AE/SAE review	←===→					X[3]	←========================→			
Concomitant medication review	←===→					X[3]	←========================→			[3] Phone call made to the subject to check AEs and concomitant medications and confirm availability for FUV3
TNSS scores (diary card)	←===→									Captured daily AM and PM up until the end of FUV 2

Procedure	Follow up Visit 1 (FUV1)		Follow-up Visit 2 (FUV2)			2 month follow-up phone call (Week 16)	Follow up Visit 3 (FUV3)			Notes
	Week 9 (1 week after DV8)	24 hours later	Week 11 (3 weeks after DV8)	Week 12 (4 weeks after DV8)	24 hours later		Week 19 (11 weeks after DV8)	Week 20 (12 weeks after DV8)	24 hours later	
PEF (diary card)	←======================================→							X		Captured daily AM and PM up until the end of FUV2, and then checked again in the unit following the final BAC at FUV3
Diary card review	X									Review diary card at every visit until the end of FUV 2.
Blood PAX collection				X						PAX tubes for /AFFY chip gene expression in blood. Collected prior to the bronchial allergen challenge at FUV2
Saline and bolus BAC challenge plus associated spirometry measurements	X			X				X		Including baseline FEV_1 assessment and repeat FEV_1 measurements up to 10 hours post BAC
FeNO measurement	X	X		X	X			X	X	Collect prior to sputum induction
Sputum induction and processing for BAC associated sample collection of allergic biomarkers		X	X		X		X		X	Sputum induction pre-BAC and 24 hours after BAC.
BAC associated sample collection of allergic biomarkers in blood	X[1,2]	X[2]		X[1,2]	X[2]			X[1,2]	X[2]	[1]Blood samples for PBMC preparations (pre-BAC and 6 hours after BAC)<br>[2] Blood sample for haematology (WBC count with differential) pre-BAC and 24 hours after BAC

Procedure	Follow up Visit 1 (FUV1)		Follow-up Visit 2 (FUV2)			2 month follow-up phone call (Week 16)	Follow up Visit 3 (FUV3)			
	Week 9 (1 week after DV8)	24 hours later	Week 11 (3 weeks after DV8)	Week 12 (4 weeks after DV8)	24 hours later		Week 19 (11 weeks after DV8)	Week 20 (12 weeks after DV8)	24 hours later	Notes
Nasal allergen challenge/administration		X			X					Commence after the 24-hour BAC sputum and blood biomarker samples have been taken at FUV1 and FUV2
Nasal filter biomarker collection		X			X					Collect pre-NAC, 5 minutes and 6 hours after NAC.
Nasal fluid biomarker collection		X			X					Collect nasal lavage pre-NAC, 5 minutes and at least 6 hours after NAC.<br>Collect nasal mucosa sample at least 6 hours after NAC.
Intradermal allergy testing		X			X					Intradermal challenge to be administered after NAC.<br>Measurements to be done at 15 minutes and 6 hours post intradermal challenge.
Return diary card, PEF meters, rescue medication and thermometers					X					

REVISED TEXT

Section 7.1.1. Time and Events Table – Screening Visit(s)

Procedure	Screening Visit 1 (Day -28 to Day -22)	Screening Visit 2 (Day -21 to Day -3)	24 hours after Screening Visit 2 (Day -20 to Day -2)	Notes
Informed consent signing	X			
Inclusion and exclusion criteria/eligibility review	X	X		
Demographics	X			
Medical history (including substance usage and family history of premature CV disease)	X			
Past and current medical conditions including cardiovascular medical history	X			
Concomitant medication review	X			
Full physical exam including height and weight	X			
Urine drug/alcohol screen	X			
Urine or serum pregnancy test (all females)	X			No women of childbearing potential are being enrolled. However this test is needed to confirm pregnancy status at screening.
Serum FSH and estradiol for post-menopausal females	X			
HIV, Hep B and Hep C screen	X			
Laboratory assessments (including liver chemistries, haematology, clinical chemistry and urinalysis)	X			
Screening Spirometry assessment	X			To confirm FEV_1 > 70% predicted
SAE Collection	X	X	X	
12-lead ECG	X			
Vital signs	X			
Skin prick test	X			To confirm eligibility
FeNO measurement	X		X	Collected prior to sputum induction
Saline and incremental BAC challenge plus associated spirometry measurements		X		Including pre-procedure FEV_1 assessment and repeat FEV_1 measurements up to 10 hours post BAC
Sputum induction and processing for BAC associated sample collection	X		X	Sputum inductions must be separated by at least 3 days
Laboratory assessments (haematology only)		X	X	Blood samples should be taken pre-BAC on SV2 and on the following day at 24hrs post BAC
Blood samples for PBMC preparations		X		Blood samples for PBMC preparations (pre-BAC and 6 hours after BAC at screening visit 2)

Procedure	Screening Visit 1 (Day -28 to Day -22)	Screening Visit 2 (Day -21 to Day -3)	24 hours after Screening Visit 2 (Day -20 to Day -2)	Notes
Provide diary card, PEF meters, rescue medication and thermometers to subjects		X		Only given out to subjects who are eligible following the BAC. Thermometers are to be given to record temperature as part of capturing AE information.
NAC			X	Commence after the 24-hour BAC sputum and blood biomarker samples have been taken.<br>Only to be done once subjects have completed a successful BAC.
Nasal lavage			X	Collect nasal lavage to provide a background sample. Repeat nasal lavage pre-NAC, then again 5 minutes and at least 6 hours post-NAC.
Nasal filter collection			X	Collect nasal filter sample to provide a background pre-wash sample, after nasal wash repeat nasal filter sampling pre-NAC, then again 5 minutes and at least 6 hours post-NAC.
Nasal scrape			X	Nasal mucosa sample to be collected at least 6 hours post-NAC.
ID challenge			X	ID challenge to be administered after NAC.<br>Measurements to be made 15 minutes and 6 hours post ID challenge

Section 7.1.2. Time and Events Table – Dosing visit (DV) 1 (Day 1) – DV8 (Week 8) and Early Withdrawal Visit (EW)

Procedure	Pre-dose	Dose	Post-dose				Early-Withdrawal	Notes
		0 hour	20 mins	1 hour	12 hours	24 hours		
Outpatient visit	X					X	X	Subjects get discharged 1-2 hours after dosing.
Inclusion and exclusion criteria/eligibility review	X							Only on DV1
Diary card collection and review	X						X	Collect and review diary card at every dosing visit
AE/SAE review	X						X	
Concomitant medication review	X						X	
12-lead ECG	X			X		X	X	Perform on DV1 and DV8 only
Vital signs (including temperature)	X[1]			X[1]		X[2]	X	[1] Perform on DV1-8<br>[2] Perform on DV1, 4 and DV8 only
Laboratory assessments (include liver chemistries, haematology, clinical chemistry and urinalysis)	X					X	X	Collect samples for laboratory assessments on DV1 and DV8<br>Not required at EW if this occurs during FU
Blood sample for PK analysis	X		X	X				Collect samples on DV1, DV4 and DV8.<br>Not required at EW if this occurs during FU
Genetic sample	X							Take one sample on any of the dosing visits after genetics consent.<br>Not required at EW if this occurs during FU
FeNO measurement	X							Perform FeNO measurements pre-dose on all dosing visits 1-8
Nasal examination	X					X		Conducted pre-dose at all study visits and 24 hours post-dose on DV1, DV4 and DV8
Nasal lavage	X					X		Collect nasal lavage samples pre-dose and 24hrs post-dose on DV1, DV4 and DV8

Procedure	Pre-dose	Dose	Post-dose				Early-Withdrawal	Notes
		0 hour	20 mins	1 hour	12 hours	24 hours		
Blood sample for biomarker analysis	X					X		Collect blood for serum samples and PBMC preparations pre-dose and 24 hrs post-dose on DV1 and DV8
Blood sample for RNA analysis	X(3)					X(4)		PAX-gene tubes for TLR7/AFFY chip gene expression in blood collected pre-dose on DV1(3) and 24 hrs after DV8(4)
Call RAMOS	X							Randomisation will occur is at Visit 1 via the IRT. Sites should also call RAMOS at DV5.
Dispensing of study medication	X							One device will be assigned on DV1. The second device will be assigned on DV5. All devices remain in the unit.
Study drug administration		X						All doses are administered in the unit.
Sputum induction and processing for BAC associated sample collection						X		Sputum sample is conditional on having obtained a sputum sample from the subject at screening. Perform sputum induction after collection of 24 hour post-dose DV8 blood and nasal samples (as pre BAC sample for FUV1).
Follow up phone call					X[5]	X[6]		[5] Following at least the first two doses of study treatment to assess tolerability<br>[6] On DV 2, 3, and 5-7, a follow-up phone call will be made approximately 24 hours post-dose to check if any CRS events have occurred, and if they have, that they have been accurately recorded in the diary card and they will then be transcribed into the eCRF. For DV1, 4 and 8 the subjects shall be assessed on their return to the unit the following day.

Section 7.1.3. Time and Events Table – Follow up visits (FUV1 to 3) (Weeks 9 to 20)

Procedure	Follow up Visit 1 (FUV1)		Follow-up Visit 2 (FUV2)			2 month follow-up phone call (Week 16)	Follow up Visit 3 (FUV3)			
	Week 9 (1 week after DV8)	24 hours later	Week 11 (3 weeks after DV8)	Week 12 (4 weeks after DV8)	24 hours later		Week 19 (11 weeks after DV8)	Week 20 (12 weeks after DV8)	24 hours later	Notes
AE/SAE review	←==→					X[3]	←========================→			
Concomitant medication review	←==→					X[3]	←========================→			[3] Phone call made to the subject to check AEs and concomitant medications and confirm availability for FUV3
Diarycard collection, review and dispensing	X									Review diary card at every visit until the end of FUV 2
FeNO measurement	X	X		X	X			X	X	Collect prior to sputum induction
Blood sample for RNA analysis				X						PAX tubes for /AFFY chip gene expression in blood. Collected prior to the bronchial allergen challenge at FUV2
Saline and bolus BAC challenge plus associated spirometry measurements	X			X				X		Including pre-procedure FEV_1 assessment and repeat FEV_1 measurements up to 10 hours post BAC
Sputum induction and processing for BAC associated sample collection		X	X		X		X		X	Sputum induction pre-BAC and 24 hours after BAC
Laboratory assessments (haematology only)	X	X		X	X			X[1]	X	Blood samples should be taken pre-BAC and on the following day at 24hrs post BAC
Blood samples for PBMC preparations	X			X				X		Blood samples for PBMC preparations (pre-BAC and 6 hours after BAC)

Procedure	Follow up Visit 1 (FUV1)		Follow-up Visit 2 (FUV2)			2 month follow-up phone call (Week 16)	Follow up Visit 3 (FUV3)			
	Week 9 (1 week after DV8)	24 hours later	Week 11 (3 weeks after DV8)	Week 12 (4 weeks after DV8)	24 hours later		Week 19 (11 weeks after DV8)	Week 20 (12 weeks after DV8)	24 hours later	Notes
Nasal lavage		X			X					Collect nasal lavage to provide a background sample. Repeat nasal lavage pre-NAC, then again 5 minutes and at least 6 hours post-NAC.
Nasal filter collection		X			X					Collect nasal filter sample to provide a background pre-wash sample, after nasal wash repeat nasal filter sampling pre-NAC, then again 5 minutes and at least 6 hours post-NA.
NAC		X			X					Commence after the 24-hour BAC sputum and blood biomarker samples have been taken at FUV1 and FUV2
ID challenge		X			X					ID challenge to be administered after NAC. Measurements to be made at 15 minutes and 6 hours post ID challenge.
Nasal scrape		X			X					Nasal mucosa sample to be collected at least 6 hours post-NAC
Return diary card and thermometers					X					
Return PEF meters and rescue medication									X	

Section 7.3.1. Bronchial allergen challenges and spirometry: This section has been simplified and the detail moved to the SRM

PREVIOUS TEXT

Bronchial allergen challenges will be conducted at screening, and at three follow-up visits (see Time and Events Tables in Section 7.1).

Baseline FEV_1 measurements for the challenge will first be taken using saline. The highest of the measurements will be taken as the saline baseline value and recorded in the eCRF. This value will be the baseline for the allergen challenge. If a fall in FEV_1 of >10% is observed after saline, the subject will not proceed to allergen inhalation. At screening, subjects will inhale increasing concentrations of allergen during the bronchial allergen challenge. FEV_1 will be measured after each inhalation. The final concentration of allergen will be determined when a fall in FEV_1 of ≥ 20 % from the saline baseline value is achieved. For the subject to be eligible, the screening bronchial allergen challenge must demonstrate both an early and late asthmatic response. The early asthmatic response must include a fall in FEV_1 of ≥ 20% from the saline value, on at least one occasion, between 5 and 30 minutes after the final concentration of allergen. The late asthmatic response must include a fall in FEV_1 of ≥ 15% from the saline value, on at least three occasions, two of which must be consecutive, between 4 and 10 hours after the final concentration of allergen. FEV_1 measurements will be taken at specified time intervals after the final inhalation of allergen up to 10 hours.

Following the final FEV_1 measurement, the subject will receive rescue salbutamol until the FEV_1 returns to within 10% of the pre-saline value. If a subject experiences serious discomfort the test will be discontinued and rescue medication administered.

The allergen dose eliciting a fall in FEV_1 of ≥ 20 % from the saline baseline value at screening will be used for bronchial allergen challenges administered at the follow-up visits and FEV_1 measurements will be taken at specified time intervals up to 10 hours.

Detailed description of the bronchial allergen challenge technique and assessments can be found in the SRM

REVISED TEXT

BAC will be conducted at screening, and at three follow-up visits (see Time and Events Tables in Section 7.1). At the Screening Visit incremental doses of allergen will be administered and the total dose inducing an EAR and LAR used to calculate a bolus dose for use at the 3 FU visits. A detailed description of the BAC technique and assessments can be found in the SRM. All sites participating in the study will be trained in the same methodology to ensure consistency.

Following the final FEV_1 measurement, subjects will receive rescue salbutamol until the FEV_1 returns to within 10% of the pre-saline value. If a subject experiences serious discomfort the test will be discontinued and rescue medication administered.

Section 7.3.2. Intranasal and intradermal allergen challenge

PREVIOUS TEXT

Intranasal and intradermal allergen challenges will be conducted approximately 24 hours after the bronchial allergen challenge at screening and at follow-up visits 1 and 2. These challenges will only be conducted after subjects have completed successful bronchial allergen challenges.

A detailed description of the intranasal and intradermal allergen challenges can be found in the SRM.

In some regions/countries specific challenges agents may not be approved or available for use in ID challenge, in which case this assessment shall be omitted.

REVISED TEXT

NAC and ID challenge will be conducted approximately 24 hours after the bronchial allergen challenge at screening and at follow-up visits 1 and 2.

A detailed description of the NAC and ID challenges can be found in the SRM.

Section 7.3.4. TNSS: Updated as transcription to CRF does not necessarily have to be done by site staff.

PREVIOUS TEXT

All data will be transcribed from the diary card into the CRF by the site staff at each clinic visit

REVISED TEXT

All data will be transcribed from the diary booklet into the CRF.

Section 7.3.6. Fractional Exhaled Nitric Oxide (FeNO): Updated to reflect that equipment will not be provided centrally.

PREVIOUS TEXT

FeNO will be measured using a handheld electronic device. Measurements will be obtained in accordance with the [ATS/ERS Recommendations for Standardized Procedures for the Online and Offline Measurement of Exhaled Lower Respiratory Nitric Oxide and Nasal Nitric Oxide, 2005]. All sites will use standardised equipment provided by a central vendor. FeNO measurements will be interpreted in accordance with the [Official ATS Clinical Practice Guideline: Interpretation of Exhaled Nitric Oxide Levels (FeNO) for Clinical Applications, 2011].

REVISED TEXT

FeNO will be measured using a handheld electronic device. Measurements will be obtained in accordance with the [ATS/ERS Recommendations for Standardized Procedures for the Online and Offline Measurement of Exhaled Lower Respiratory Nitric Oxide and Nasal Nitric Oxide, 2005]. FeNO measurements will be interpreted in accordance with the [Official ATS Clinical Practice Guideline: Interpretation of Exhaled Nitric Oxide Levels (FeNO) for Clinical Applications, 2011].

Section 7.4. Safety: Updated to describe the role of the SRT and to reflect the inclusion of an external consultant.

PREVIOUS TEXT

Planned time points for all safety assessments are listed in the Time and Events Table (Section 7.1). Additional time points for safety tests (such as vital signs, physical exams and laboratory safety tests) may be added during the course of the study based on newly available data to ensure appropriate safety monitoring

REVISED TEXT

Planned time points for all safety assessments are listed in the Time and Events Table (Section 7.1). Additional time points for safety tests (such as vital signs, physical exams and laboratory safety tests) may be added during the course of the study based on newly available data to ensure appropriate safety monitoring.

The Safety Review Team (SRT) is a GSK cross-functional team reviewing all available safety data related to the project, including in-stream data from this study, in an ongoing manner. The SRT is an internal GSK requirement put in place to ensure holistic evaluation of the safety profile of an investigational product with systematic, periodic and documented reviews of available safety data, with the appropriate communication and escalation of new findings that have the potential to impact patient safety.

The SRT for this project will review data from this study in collaboration with an independent external (to GSK) expert reviewer.

Section 7.4.7. PEF monitoring: Final Paragraph updated as transcription to CRF does not necessarily have to be done by site staff.

PREVIOUS TEXT

All information from the diary card will be transcribed into the eCRF by the site staff at each clinic visit

REVISED TEXT

All information from the diary card will be transcribed into the eCRF.

Section 7.4.7.: PEF monitoring: Text updated to clarify that these are being used for safety monitoring at home. Assessment in clinic at FV3 will not be required as subject will be attending the clinic the day after their final BAC.

PREVIOUS TEXT

Subjects will be given a PEF peak flow meter at screening, along with a diary card. Subjects will record their PEF readings each morning and evening onto the diary card every day from the start of study treatment on Dosing Visit 1 until the 4 week follow-up visit. Subjects will also have their PEF levels checked in the unit after administration of the final bronchial allergen challenge at the 12 week follow-up visit.

REVISED TEXT

Subjects will be given a PEF peak flow meter at screening, along with a diary card. This will be used for safety monitoring after the BACS. Subjects will record their PEF readings each morning and evening onto the diary card every day from the start of study treatment on DV 1 until the 4 week follow-up visit.

The subject will be instructed to contact the investigator if at any time during the study the PEF measurements go outside the normal range for the subject. These instructions will be clearly printed on the diary card.

Section 7.6.1.1. PD blood samples

PREVIOUS TEXT

Blood samples will be collected before and 24 hours post treatment on Dosing Visits 1 and 8 for serum to measure levels of TLR-7-induced blood PD biomarkers.

Blood will be collected before and 24 hours after treatment on Dosing Visits 1 and 8 and prior to bronchial allergen challenges for preparation of peripheral blood mononuclear cells (PBMC). These samples may be used for CHIP cytometry for cellular subset phenotyping. If this biomarker analysis is performed it may be reported separately.

REVISED TEXT

Blood samples will be collected before and 24 hours post treatment on DV 1 and 8 for serum to measure blood PD biomarkers.

Blood will be collected before and 24 hours after treatment on DV 1 and 8 and prior to bronchial allergen challenges for preparation of peripheral blood mononuclear cells (PBMC). These samples may be used for CHIP cytometry for cellular subset phenotyping. If this biomarker analysis is performed it may be reported separately.

Section 7.6.1.2. Blood PAX collection

PREVIOUS TEXT

Blood PAX samples for AFFYCHIP gene expression (mRNA transcriptome) will be collected before dosing on Dosing Visit 1, 24 hours after Dosing Visit 8 and prior to BAC on FUV2.

REVISED TEXT

Blood PAX samples for AFFYCHIP gene expression (mRNA transcriptome) will be collected before dosing on DV 1, 24 hours after DV 8 and prior to BAC on FUV2.

Section 7.6.1.3. PD nasal lavage samples

PREVIOUS TEXT

Nasal lavage samples to measure TLR-7-induced nasal fluid PD biomarkers will be collected before and 24 hours post treatment on Dosing Visits 1, 4 and 8.

REVISED TEXT

Nasal lavage samples to measure PD biomarkers will be collected before and 24 hours post treatment on DV1, DV4 and DV8.

Section 7.6.1.6. :Nasal allergen challenge associated biomarkers in nasal fluid samples

PREVIOUS TEXT

Nasal filter and nasal lavage samples to measure exploratory allergic biomarkers will be collected at baseline, then after a nasal wash, samples will be collected before the nasal allergen administration (NAC), and at 5 minutes and 6 hours after NAC (at screening and at FUV1 and FUV2).

REVISED TEXT

Nasal filter and nasal lavage samples to measure exploratory biomarkers will be collected at the timepoints indicated in the Time and Events Tables Section 7.1.

Section 7.6.1.7. Cellular profile of nasal mucosa

PREVIOUS TEXT

Nasal scrapes will be collected after NAC following the completion of the collection of the 6 hour nasal lavage and nasal filter samples (at screening and at FUV1 and FUV2). Samples of suitable quality may be used for cellular analysis by CHIP cytometry or RNA-sequencing of single cells (if subjects have given informed consent for genetic analysis techniques). If this biomarker analysis is performed it may be reported separately.

REVISED TEXT

Nasal scrapes may be collected after NAC following the completion of the collection of the 6 hour nasal lavage and nasal filter samples (at screening and at FUV1 and FUV2). Samples of suitable quality may be used for cellular analysis for example by CHIP cytometry or RNA-sequencing of single cells (if subjects have given informed consent for genetic analysis techniques). If this biomarker analysis is performed it may be reported separately.

Section 7.7.1.5.:Bronchial allergen challenge (BAC) associated biomarkers in blood samples – Updated to reflect that RNA analysis of single cells will not be performed.

PREVIOUS TEXT

Blood samples will be collected before, 6 hours and 24 hours after each BAC:

For haematology, (WBC with differential) for blood eosinophil counts (before and 24 hours after BAC)

To prepare PBMC that may be used for CHIP cytometry or RNA sequencing of single cells (if patients give informed consent for genetic analysis techniques) before and 6 hours after BAC. If this biomarker analysis is performed it may be reported separately.

REVISED TEXT

Blood samples will be collected before, 6 hours and 24 hours after each BAC:

For haematology, (WBC with differential) for blood eosinophil counts (before and 24 hours after BAC)

To prepare PBMC that may be used for CHIP cytometry (before and 6 hours after BAC). If this biomarker analysis is performed it may be reported separately.

AMENDMENT [1]

Where the Amendment Applies

This amendment applies across any site that will be used in the study.

Summary of Amendment Changes with Rationale

Section 4.6.1 and Section 7.1.1 have been adjusted slightly to make it clearer that subjects will be provided with a thermometer to be able to record temperature as part of capturing adverse event (AE) information.

In Section 5.2, exclusion criterion 13 has been updated to reflect the revised recommended alcohol consumption guidelines.

List of Specific Changes

Section 4.6.1, Risk assessment table, first paragraph

PREVIOUS TEXT

Each subject will receive a phone call approximately 12 hours following administration of at least the first two doses to assess tolerability. They will be provided with a thermometer to measure body temperature **as needed**.

REVISED TEXT

Each subject will receive a phone call approximately 12 hours following administration of at least the first two doses to assess tolerability. They will be provided with a thermometer to measure body temperature.

Section 5.2, Exclusion criteria, point 13

PREVIOUS TEXT

RELEVANT HABITS
13. History of regular alcohol consumption within 6 months of the study defined as:<br>An average weekly intake of >21 units for males or >14 units for females. One unit is equivalent to 8 g of alcohol: a half-pint (~240 ml) of beer, 1 glass (125 ml) of wine or 1 (25 ml) measure of spirits.

REVISED TEXT

RELEVANT HABITS
13. History of regular alcohol consumption within 6 months of the study defined as:<br>An average weekly intake **of >14 units for males and females**. One unit is equivalent to 8 g of alcohol: a half-pint (~240 ml) of beer, 1 glass (125 ml) of wine or 1 (25 ml) measure of spirits.

Section 7.1.1, Time and Events table, Screening Visit(s)

PREVIOUS TEXT

Procedure	Screening Visit 1 (Day -28 to Day -22)	Screening Visit 2 (Day -21 to Day -3)	24 hours after Screening Visit 2 (Day -20 to Day -2)	Notes
Informed consent signing	X			
Inclusion and exclusion criteria/eligibility review	X	X		
Demographics	X			
Medical history (including substance usage and family history of premature CV disease)	X			
Past and current medical conditions including cardiovascular medical history	X			
Concomitant.medication review	X			
Full physical exam including height and weight	X			
Urine drug/alcohol screen	X			
Urine or serum pregnancy test (all females)	X			No women of childbearing potential are being enrolled. However this test is needed to confirm pregnancy status at screening.
Serum FSH and estradiol for post-menopausal females	X			
HIV, Hep B and Hep C screen	X			
Laboratory assessments (including liver chemistries, haematology, clinical chemistry and urinalysis)	X			
Spirometry	X			To confirm FEV_1 > 70% predicted
12-lead ECG	X			
Vital signs	X			
Skin prick test	X			To confirm eligibility
FeNO measurement	X		X	Collected prior to sputum induction
BAC and spirometry		X		Including repeat FEV_1 measurements up to 10 hours post BAC
Sputum induction and processing for BAC associated sample collection	X		X	Sputum induction pre-BAC and 24 hours after BAC
BAC associated sample collection of allergic biomarkers in blood		X[1,2]	X[2]	[1] Collect blood samples for PBMC preparations (pre-BAC and 6 hours after BAC)<br>[2] Collect blood sample for haematology (WBC count with differential) pre-BAC and 24 hours after BAC
Provide diary card, PEF meters, rescue medication and thermometers to subjects		X		Only given out to subjects who are eligible following the BAC. Thermometers are to be given to

Procedure	Screening Visit 1 (Day -28 to Day -22)	Screening Visit 2 (Day -21 to Day -3)	24 hours after Screening Visit 2 (Day -20 to Day -2)	Notes
				record temperature as part of capturing AE information, **if needed.**
Nasal allergen challenge administration (NAC)			X	Commence after the 24-hour BAC sputum and blood biomarker samples have been taken .. Only to be done once subjects have completed a successful BAC.
Nasal filter biomarker collection			X	Collect pre-NAC, 5 minutes and at 6 hours after NAC.
Nasal fluid biomarker collection			X	Collect nasal lavage pre-NAC, 5 minutes and at least 6 hours after NAC. Collect nasal mucosa sample at least 6 hours after NAC.
Intradermal allergy testing			X	Intradermal challenge to be administered after NAC. Measurements to be made 15 minutes and 6 hours post intradermal challenge

REVISED TEXT

Procedure	Screening Visit 1 (Day -28 to Day -22)	Screening Visit 2 (Day -21 to Day -3)	24 hours after Screening Visit 2 (Day -20 to Day -2)	Notes
Informed consent signing	X			
Inclusion and exclusion criteria/eligibility review	X	X		
Demographics	X			
Medical history (including substance usage and family history of premature CV disease)	X			
Past and current medical conditions including cardiovascular medical history	X			
Concomitant.medication review	X			
Full physical exam including height and weight	X			
Urine drug/alcohol screen	X			
Urine or serum pregnancy test (all females)	X			No women of childbearing potential are being enrolled. However this test is needed to confirm pregnancy status at

Procedure	Screening Visit 1 (Day -28 to Day -22)	Screening Visit 2 (Day -21 to Day -3)	24 hours after Screening Visit 2 (Day -20 to Day -2)	Notes
				screening.
Serum FSH and estradiol for post-menopausal females	X			
HIV, Hep B and Hep C screen	X			
Laboratory assessments (including liver chemistries, haematology, clinical chemistry and urinalysis)	X			
Spirometry	X			To confirm FEV_1 > 70% predicted
12-lead ECG	X			
Vital signs	X			
Skin prick test	X			To confirm eligibility
FeNO measurement	X		X	Collected prior to sputum induction
BAC and spirometry		X		Including repeat FEV_1 measurements up to 10 hours post BAC
Sputum induction and processing for BAC associated sample collection	X		X	Sputum induction pre-BAC and 24 hours after BAC
BAC associated sample collection of allergic biomarkers in blood		X[1,2]	X[2]	[1] Collect blood samples for PBMC preparations (pre-BAC and 6 hours after BAC)<br>[2] Collect blood sample for haematology (WBC count with differential) pre-BAC and 24 hours after BAC
Provide diary card, PEF meters, rescue medication and thermometers to subjects		X		Only given out to subjects who are eligible following the BAC. Thermometers are to be given to record temperature as part of capturing AE information.
Nasal allergen challenge administration (NAC)			X	Commence after the 24-hour BAC sputum and blood biomarker samples have been taken ..<br>Only to be done once subjects have completed a successful BAC.
Nasal filter biomarker collection			X	Collect pre-NAC, 5 minutes and at 6 hours after NAC.
Nasal fluid biomarker collection			X	Collect nasal lavage pre-NAC, 5 minutes and at least 6 hours after NAC.<br>Collect nasal mucosa sample at least 6 hours after NAC.
Intradermal allergy testing			X	Intradermal challenge to be administered after NAC. Measurements to be made 15 minutes and 6 hours post intradermal challenge
