# Supplementary material for: Intranasal GSK2245035, a Toll-like receptor 7 agonist, does not attenuate the allergen-induced asthmatic response in a randomized, double-blind, placebo-controlled experimental medicine study
Source: PLoS One. 2020 Nov 9;15(11):e0240964. doi: 10.1371/journal.pone.0240964 (PMC7652256; doi:10.1371/journal.pone.0240964)
Supplement: S6 File — (DOCX) [file pone.0240964.s010.docx]

## S1 Ethics committees. Details of Independent Ethics Committees/Institutional Review Boards.

| Study site location | Name of Independent Ethics Committee/Institutional Review Boards |
| --- | --- |
| UK | Reference number: 16/WA/0213 Wales REC 3, Castlebridge 4 15 – 19 Cowbridge Road East Health and Care Research Wales CF11 9AB, United Kingdom |
| Germany | Reference number: 7197M Ethikkommission der Medizinischen Hochschule Hannover, Carl-Neuberg-Strasse 1, Hannover, Niedersachsen, 30625, Germany |
